# Implications of oral streptococcal bacteriophages in autism spectrum disorder

**DOI:** 10.1038/s41522-022-00355-3

**Published:** 2022-11-18

**Authors:** Zhan Tong, Xin Zhou, Yanan Chu, Tianxu Zhang, Jie Zhang, Xiaoyan Zhao, Zuqun Wang, Rui Ding, Qingren Meng, Jun Yu, Juan Wang, Yu Kang

**Affiliations:** 1 Beijing Institute of Genomics, Chinese Academy of Sciences/China National Center for Bioinformation, Beijing, 100101 China; 2 Department of Biomedical Informatics, School of Basic Medical Sciences, Peking University, Beijing, 100191 China; 3 Department of stomatology, Peking University Third Hospital, Beijing, 100191 China; 4 Autism research center of Peking University Health Science Center, Beijing, 100191 China; 5 Department of child healthcare, Xi’an Children’s Hospital, Xi’an, 710003 China; 6 School of Medicine, Southern University of Science and Technology, Shenzhen, 518055 China; 7 University of Chinese Academy of Sciences, Beijing, 100190 China

**Keywords:** Metagenomics, Microbiome

## Abstract

Growing evidence suggests altered oral and gut microbiota in autism spectrum disorder (ASD), but little is known about the alterations and roles of phages, especially within the oral microbiota in ASD subjects. We enrolled ASD (*n* = 26) and neurotypical subjects (*n* = 26) with their oral hygiene controlled, and the metagenomes of both oral and fecal samples (*n* = 104) are shotgun-sequenced and compared. We observe extensive and diverse oral phageome comparable to that of the gut, and clear signals of mouth-to-gut phage strain transfer within individuals. However, the overall phageomes of the two sites are widely different and show even less similarity in the oral communities between ASD and control subjects. The ASD oral phageome exhibits significantly reduced abundance and alpha diversity, but the Streptococcal phages there are atypically enriched, often dominating the community. The over-representation of Streptococcal phages is accompanied by enriched oral Streptococcal virulence factors and *Streptococcus* bacteria, all exhibiting a positive correlation with the severity of ASD clinical manifestations. These changes are not observed in the parallel sampling of the gut flora, suggesting a previously unknown oral-specific association between the excessive Streptococcal phage enrichment and ASD pathogenesis. The findings provide new evidence for the independent microbiome-mouth-brain connection, deepen our understanding of how the growth dynamics of bacteriophages and oral microbiota contribute to ASD, and point to novel effective therapeutics.

## Introduction

Autism spectrum disorder (ASD) is a complex neurological and developmental illness that appears in the first years of life and leads to life-long disability^1^. The incidence and prevalence of ASD have remarkably increased in recent years^2^, but effective medical interventions are limited as both the etiology and pathogenesis of ASD are not yet fully understood^3–5^. One of the major pathophysiology features of ASD is chronic inflammation in both brain and intestinal mucosa^6^, and growing evidence has indicated the association of human gut microbiota to inflammation development in ASD^7–10^.

Oral microbiota, residing at the entry of the digestive tract, harbors distinct profiles of microbial species differing from those of the guts. Although very limited in biomass and complexity, altered oral microbiota has been described in some human diseases^11–13^, as well as in many brain disorders, including ASD^14–16^, Alzheimer’s Disease^17^, dementia^18^, and bipolar disorders^19^, and such a diverse disease profile suggests potential oral-brain crosstalk. However, more evidence is needed for the full understanding of the exact mechanisms of how oral microbiota contributes to ASD and whether its dynamics correlate to that of the gut microbiota.

Within the human microbial flora, bacteriophages (or phages) constitute a major constituent as an important driving force for microbiota diversity by facilitating nutrient turnover^20^ and horizontal gene transfer^21^. Metagenomic sequencing has revealed extensive, diverse, and dynamic populations of phages in the human intestinal microbiota^22–24^. Although highly diverse among individuals^25^, the dynamics of the gut phage communities have been reported to be correlated with distinct disease states^26–28^. In addition to directly modulating bacterial propagation, phages also interact with mucosal epithelia and immune cells, having impacts on the inflammatory status and microbial colonization^29,30^. Despite these observations and potential implications for the pathogenesis of neurological diseases, such as ASD, in-depth molecular evaluation of phages, especially in human oral microbial flora, in the context of neurological and mental disorders, has yet to be described.

In this study, we recruit a cohort of ASD children that are readily differentiated from the typically-developed control in their oral and gut metagenomes and whose phage communities of both sites are extensively explored based on deep shotgun sequencing. Having analyzed high-quality phage genomes from the metagenomic assemblies, we observe extensive and diversified phage communities in the oral flora comparable to those of the gut. Although a few signals of phages transferring from the oral to the gut flora have been observed, within-individual connections between the two sites are not prominent. The oral phage communities of ASD are distinguishable from the control in significantly reduced diversity but remarkably enriched Streptococcal phages that often dominate the community. The abundance of Streptococcal virulence factors and *Streptococcus* bacteria are also elevated, together with the phage abundance that correlates closely to the severity of ASD clinical manifestations. These alterations are not observed in the parallel ASD gut phage communities, suggesting the oral-specific association of the Streptococcal phages to ASD pathogenesis and an oral-brain cross-talk that is independent of phages in the gut.

## Results

### The oral and gut phage communities in the studied subjects

We enrolled 52 participants for this study (Table 1), including 26 ASD children and 26 age- and gender-matched typically developed healthy control (HC) subjects. Dental plaques, after standard examination and hygiene cleaning, are used to represent the oral microbial community, together with the corresponding fecal samples as parallel. The case-and-control subjects are similar in the number of cavities and filled teeth, tooth scale index, and calculus index, showing no significant differences in oral hygiene.

**Table 1:** Summary of subject characteristics for the samples included in the study.

	ASD (*n* = 26)	HC (*n* = 26)	*P* value
*Demographics*			
Age	4.13 (0.95)	4.04 (0.89)	0.8
Gender			1^#^
Male	23	23	
Female	3	3	
Body mass index	18.81 (6.08)	16.41 (2.74)	0.035^*^
*Oral/gut factor*			
Number of checked teeth	20.23 (0.86)	20.08 (0.4)	0.58
Number of checked tooth surface	101.15 (4.31)	100.4 (2)	0.58
Number of cavities	1.73 (2.78)	1.52 (2.08)	0.98
Number of caries tooth surface	3.08 (6.29)	1.88 (2.71)	0.96
Number of filling teeth	0.12 (0.59)	2.08 (0.68)	0.086
Number of filling tooth surface	0.15 (0.78)	1 (3.3)	0.083
Number of missing teeth due to caries	0.038 (0.20)	0 (0)	0.35
Trauma			1^#^
Yes	1	0	
No	24	25	
Tooth scale index	2.23 (0.71)	1.92 (0.81)	0.19
Calculus index	0.23 (0.65)	0.12 (0.33)	0.7
Dentition development			1^#^
Deciduous dentition	23	22	
Mixed dentition	3	3	
Permanent dentition	0	0	
Degree of cooperation	1.04 (0.2)	2.04 (0.89)	8.74E-06^*^
GSI	2.85 (2.17)	NA	NA
*Neuropsychiatric characteristics*			
ABC	55.31 (19.73)	NA	NA
RBS-R	16.65 (11.33)	NA	NA
CARS	32.88 (8.56)	NA	NA
ADOS	23.88 (4.93)	NA	NA

^#^Chi-square test, *p-value < 0.05.

**Fig. 1:**
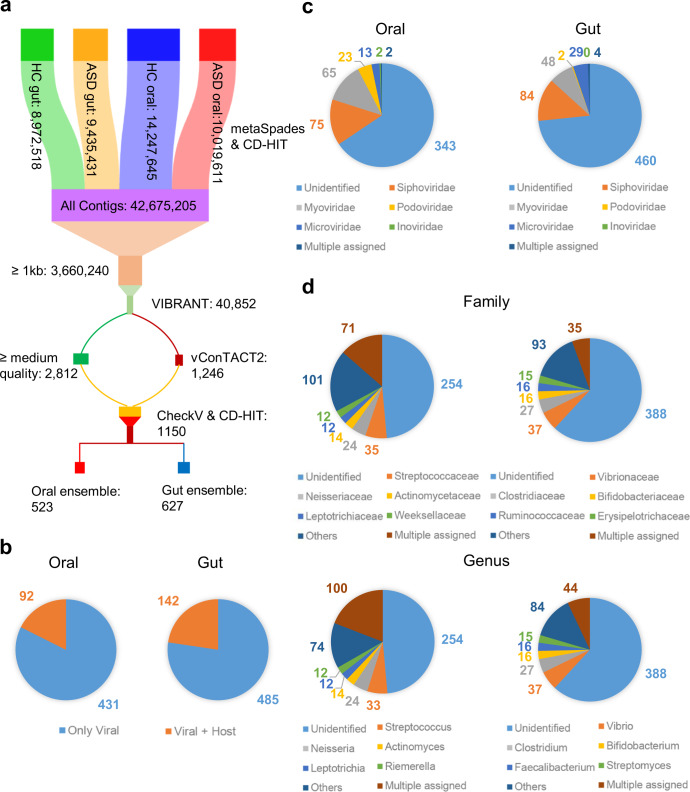
Overview of phage genomes identified in this study. **a** The Sankey diagram depicted the procedure of phage genome identification. The procedure began with de novo-assembled contigs per sample, which were pooled together based on the disease status and the body site and then clustered to obtain non-redundant sequences. After phage genome discovery and quality control, the procedure finally resulted in a total of 1150 high-quality phage genomes from the oral and gut ensembles, which included 523 and 627 phage genomes, respectively. **b** CheckV contamination analysis of phage genomes in the oral and gut ensembles. **c** Pie chart of phage genomes in the oral and gut ensembles assigned to the major phage taxa annotated by using vConTACT2, the number of phages was labeled alongside. **d** Pie chart of phage genomes in the oral and gut ensembles assigned to the major taxa of the bacterial host in the family (upper) or genus (lower) levels annotated by either vConTACT2 or CRISPR spacer matching.

Total genomic DNA was isolated from both oral and fecal samples of each subject for shotgun metagenomic sequencing. Clean reads (Supplementary Table 1) from each sample were assembled into contigs. From ~42 million total and 3.6 million >1 kb contigs, we obtained 40,852 potential phage-origin sequences, using VIBRANT, a virus identification software by iterative annotation^31^. For higher confidence on phage identity, we only use contigs of high genome quality or clustered with known viruses by vConTACT2^32^, which are 523 and 627 contigs from the oral and the fecal samples that constitute the final phage contig ensembles (Fig. 1a).

We use CheckV^33^ to re-evaluate the quality of the near-complete phage genomes (>90% genome length). Specifically, 92 (18%) and 142 (23%) phage genomes, from oral and gut samples, respectively, show signs of bacterial genomes contaminated, which are contigs containing prophage genomes (Fig. 1b). After removing the genome regions of the bacterial host, we obtain the finally purified phage genomes (Supplementary Table 2, Supplementary Table 3 listed the gut and oral phage genomes, respectively), and the genome length of oral phages is relatively shorter than that of gut phages (N50 lengths 42.7 kb vs. 55.6 kb) (Supplementary Fig. 1).

Our annotation efforts on phage genomes, using vConTACT2, identify 35% and 27% phage genomes in the oral and gut samples, respectively; the former contains more phage strains of the families of *Myoviridae* and *Podoviridae* and the latter has more *Microviridae* strains (Fig. 1c). Host information of the phages is the integration of two approaches, i.e., sequencing-based clustering by using vConTACT2 on phages of known hosts (Supplementary Fig. 2a) and CRISPR (clustered regularly interspaced short palindromic repeats) by using species-associated spacer sequences (Supplementary Fig. 2b). Taken together, the two approaches recognize 52% and 40% bacterial hosts for the oral and the gut phages, respectively, which are from a wide range of bacterial taxa, and the host of the phages are quite taxonomically diverse between the two sites (Fig. 1d).

### Inter-individual diversity and between-community correlation of oral and gut phages

**Fig. 2:**
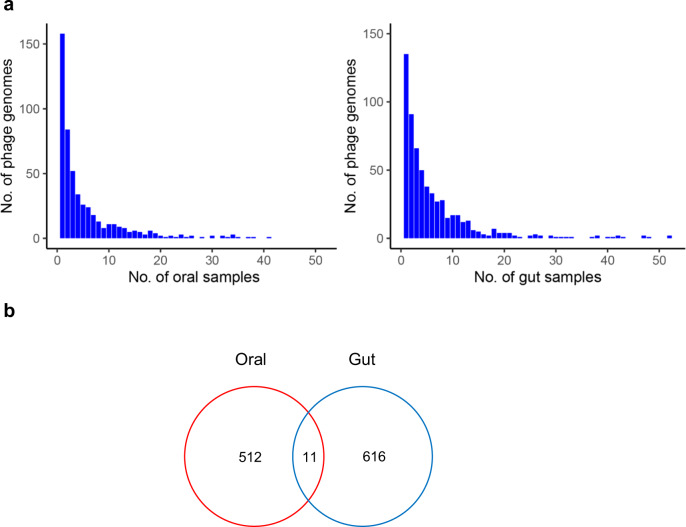
Inter-individual diversity and intra-individual connections of the oral and gut phages communities. **a** The prevalence of phage genomes (>10% genome content identity) in oral (left) and gut (right) samples from different individuals. **b** A Venn diagram displaying the shared phages between the gut and oral phage ensembles on the threshold of sequence identity >95%.

To explore the diversity of the oral phage community, we further investigate the presence of every phage among all oral samples as compared to that of the gut samples. For a given phage genome, we cluster all its relevant reads from a sample and subsequently estimate the coverage of the reads over its genome length in the sample. The procedure yields an overview of each phage genome discovered in the two communities. When we use the threshold of >80% genome length, only 63 oral and 79 gut phage genomes, i.e., 12.0% and 12.6% of the corresponding total phage genome counts, are discovered in more than one sample (Supplementary Fig. 3a), and the magnitude of community-centric diversity is rather similar. When we lose the cutoff to 50% of the genome, only 125 oral and 185 gut phage genomes are discovered in more than one sample (Supplementary Fig. 3b). When we reduce the cutoff for the length coverage to 10% of the genome, more singleton phages (158/523, 30.2%, Supplementary Fig. 3c) can be identified in the oral community than in the gut (135/627, 21.5%), suggesting greater inter-individual diversity for the oral phages (Fig. 2a).

To investigate whether there is an extensive mouth-to-gut transfer of phages similar to that of bacterial strains, such as the well-known pathogenic *Porphyromonas gingivalis* and *Actinobacillus actinomycetemcomitans*^11,34^, we first identify the shared phages between our oral and gut phage genome ensembles in a pairwise fashion, using a sequence identity cutoff of >95% of the shorter genome length. Of all 1150 phage genomes, 11 are shared by both phage communities (Fig. 2b, Supplementary Table 4), most of which are Escherichia-associated. Among them, three phages with genome length >30 kb and one phage of 5 kb are respectively assembled from the oral and gut samples of the same individual, suggesting recent mouth-to-gut transfer, whereas the other seven phages, all ~5 kb in length, assembled from samples of different subjects, possibly phages of high prevalence.

**Fig. 3:**
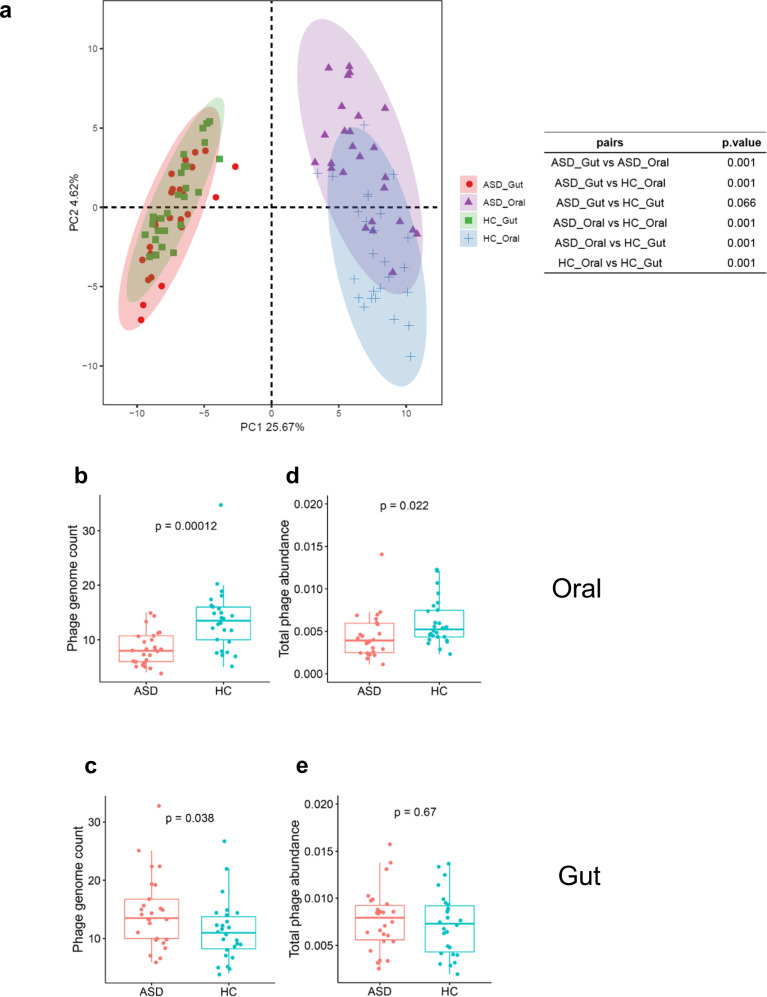
The ASD vs. HC differences in their oral and gut phage communities. **a** PCA plot of all samples based on their phage gene contents. The distances within habits are tested by PERMANOVA. **b**, **c** The difference between ASD and HC subjects in the richness of phages in oral (**b**) and fecal (**c**) samples tested by Mann–Whitney *U* test. **d**, **e** The difference between ASD and HC subjects in the total relative abundance of phages in oral (**d**) and gut (**e**) microbiota tested by Mann–Whitney *U* test. In Box–Whisker plots, the middle horizontal line refers to the median value, and the width of the box is the interquartile range (IQR) with a 1.5 IQR whisker length.

Then we test for similarity of gene content between the oral and gut phage communities by performing PCA analysis. The PCA plot shows that the oral and gut samples are significantly separated, indicating great differences between habitats (oral vs. gut) (Fig. 3a). The distances within habits are different as tested by PERMANOVA: the oral phage communities are significantly diverse between ASD and HC samples in contrast to that of the gut, implying a wider deviation of the ASD oral phage community from normality and potential of independent association to ASD pathogenesis.

We then compare the alpha diversity of the phage communities between ASD and control samples. On average, the number of phage genomes discovered in the oral samples of ASD children has always been significantly lower than that of the control (8 ± 3 vs. 14 ± 6, Mann–Whitney *U* test, *p* = 0.00012) (Fig. 3b), whereas the situation is on the opposite side in the gut phage community, where the average number of phage genomes in the ASD group has been a little higher than that of the control group (14 ± 6 vs. 11 ± 5, Mann–Whitney *U* test, *p* = 0.038) (Fig. 3c). We also compare the total abundance of the phage community in the entire microbial flora, which is represented by the proportion of reads mapped to phage genomes in all quality-filtered reads. This result shows that the total phage abundance in oral samples of the ASD subjects is still significantly lower than that of the control (Fig. 3d), but the difference is not significant in the gut (Fig. 3e), indicating reduced abundance and diversity of the oral phage community in the ASD subjects. Interestingly, the alterations in the alpha- and beta-diversity of the ASD phage community are similar to that of the bacterial community (Supplementary Fig. 4).

### Enrichment of Streptococcal phages in the oral flora of children with autism

**Fig. 4:**
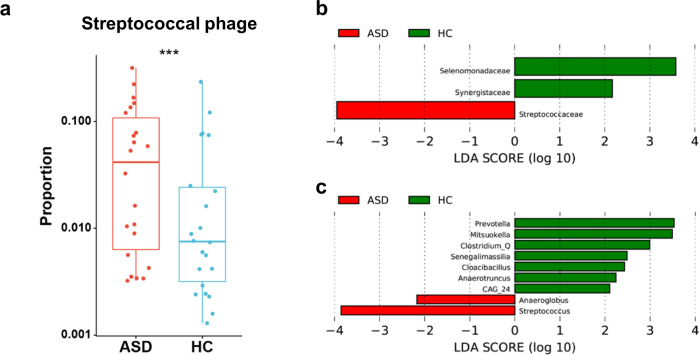
Enrichment of Streptococcal phages in the oral microbiota of ASD subjects. **a** The difference between ASD and HC subjects in their Streptococcal phage proportion in the oral phage communities, tested by the Wilcoxon signed-rank test for the quasi-paired cohort with FDR < 0.001(***). In box-whisker plots, the middle horizontal line refers to the median value, and the width of the box is the interquartile range (IQR) with 1.5 IQR whisker length. **b**, **c** LEfSe analysis is based on the relative abundance of phage groups hosted by each family (**b**) and genus (**c**) level in the oral. Only groups of effect size >2 and *p* < 0.05 (Kruskal–Wallis test) are presented with red and green bars indicating the effective size of phage groups as pro-ASD and pro-HC samples, respectively.

Next, we look for phage-related differential features distinguishing ASD and HC samples. Since the phage strains are highly diverse among individuals, we have to group phages according to the bacterial family that harbors them and compares the proportion of each group in the phage community instead of the entire metagenome. In the first comparison, we identified no phage group that is significantly different between the ASD and control samples in both oral and gut communities, possibly due to the small sample size (*U*-test, FDR < 0.05, Supplementary Fig. 5a, b). To solve this problem, we applied a recently developed analytical approach—a quasi-paired cohort, which is specific to identifying differential abundance from high-dimension data for a limited sample size and successfully applied it in various studies^7,35–37^. From the original samples, we reconstruct a quasi-paired cohort that includes 22 ASD vs. 22 control samples based on the pairwise distance of samples’ vial community profile, and eight high prevalent (present in >90% samples) differential phage groups are finally identified (Wilcoxon signed-rank test, FDR < 0.05, Supplementary Table 5). Among them, Streptococcal phages show the highest significance (FDR = 3.25 × 10^5^) and fold-change (log_2_(FC) = 1.45) that is elevated in the ASD oral phage community (Fig. 4a). For gut samples, we also reanalyzed the difference between ASD and HC samples with the quasi-paired cohort approach. Although five highly prevalent (present in >90% samples) differential phage groups are identified (Wilcoxon signed-rank test, FDR < 0.05, Supplementary Table 6), the Streptococcaceae-associated phages show no difference between the two groups of subjects. We also noticed the enrichment of Bifidobacteriaceae-associated phage in the gut of ASD subjects (Supplementary Table 6). It is possibly due to the previous supplementation of probiotic Bifidobacterium species for some ASD subjects, though they ceased at least three months before the enrollment.

To further confirm the differential abundance phage features identified above, we apply the linear discriminant analysis based on phage abundance recalculated by mapping to the most updated human gut phage database (GPD)^38^. In the oral samples, the *Streptococcaceae*-associated phage is one of the top effective size in the pro-ASD groups, reaching 3.9, as the only group over the effect size cutoff (>2) (Fig. 4b). At the genus level, phages of *Streptococcus*, the dominant genus of the *Streptococcaceae*, also has the largest effect size (Fig. 4c). These findings confirm the significant enrichment of Streptococcal phages in the oral flora of the ASD subjects, implying a potential correlation of this high-abundance phage to the pathogenesis of ASD.

### Increased Streptococcal virulence factors in the oral microbiota of children with autism

**Fig. 5:**
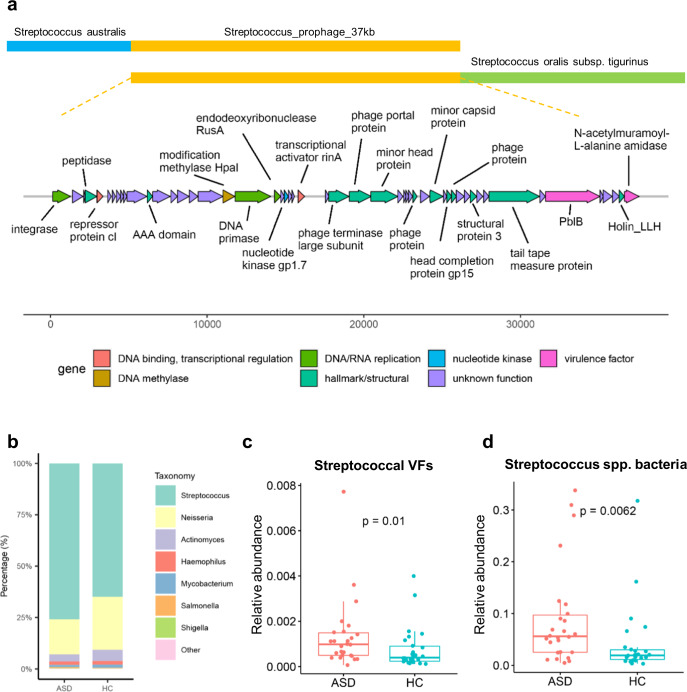
Enrichment of Streptococcal virulence factors (VFs) in the oral microbiota of ASD subjects. **a** The genome of a 37-kb Streptococcal prophage. The upper panel shows the flanking sequences of the prophage that come from two Streptococcus species. The lower panel is the genome structure of the prophage, where each gene is colored by its function categories, and the VFs are pink-colored. **b** The proportion of VFs hosted by each bacterial genus in total VF abundance between ASD and HC oral microbiota. **c** The relative abundance of Streptococcal VFs in the oral microbiota of the two groups (Mann–Whitney *U* test). **d** The relative abundance of Streptococcus bacteria in the oral microbiota of the two groups (Mann–Whitney *U* test). In Box–Whisker plots, the middle horizontal line refers to the median value, and the width of the box is the interquartile range (IQR) with 1.5 IQR Whisker length.

From one of the oral ASD samples, there is a complete 37-kb genome of a lysogenic Streptococcal prophage that carries two VFs, i.e., PblB and LytA (N-acetylmuramoyl-l-alanine amidase) (Fig. 5a). PblB is the tail protein of phage, which associates with adherence to epithelial cells and increased persistence^39^; LytA is involved in several important pathogenic processes, including fratricidal lysis during DNA competence, immune evasion, facilitation of the release of pneumolysin, and contribution to biofilm formation through the release of extracellular DNA and other structural biofilm components^40^. The flanking regions of this prophage, i.e., the upstream and downstream sequences, are from *Streptococcus australis* and *Streptococcus oralis subsp. tigurinus*, respectively (Fig. 5a), whereas the host annotation of this prophage is *Streptococcus vestibularis* or *Streptococcus pasteurianus* according to the GPD database. The broad host range of this prophage is concordant with the previously reported experiments where Streptococcal phages are often able to infect a wide range of *Streptococcus* species^41^.

As bacterial virulence factors (VFs) are often carried and disseminated by phages^42^ and critical in facilitating the invasion of bacteria and aggregating local inflammation, we systemically annotate all VFs from the assembled metagenomes of each sample by searching the most updated VF database (VFDB) and infer their abundance by summing up the reads mapped to each VF gene. In our study, 530 ± 290 VF genes have been predicted in each sample. Comparison of the VF abundance with detailed annotation of their host bacteria between the ASD and control subjects shows that VFs carried by species of *Streptococcus* spp. and *Neisseria* spp. take up the majority in oral samples, and average fractions of VFs carried by *Streptococcus* spp. in the ASD samples are substantially increased from 65% to 76% as compared to controls (Fig. 5b). Concordantly, the relative abundance of Streptococcal VFs also shows a significant increase in the ASD oral samples (Fig. 5c), as well as the overrepresentation of *Streptococcus* spp. bacteria, the host of both Streptococcal phages and VFs, in the ASD oral samples (Fig. 5d), which is consistent with previous reports based on 16S target-sequencing^15,41,43–45^.

In contrast, the situation in the gut phage community is not relevant to the alterations in the mouth, and the intestinal abundance of Streptococcal phages, Streptococcal VFs, and *Streptococcus* spp. bacteria show no difference between the two groups (Supplementary Fig. 6). Therefore, the enrichment of oral *Streptococcus* and the phages and VFs they carry in ASD is independent of alterations in the gut, and not associated with poor oral hygiene as previously supposed^46^, implying a specific connection of the oral flora to ASD pathogenesis in different mechanisms from those of the gut.

### Correlations between Streptococcal phages and clinical manifestations of autism

**Fig. 6:**
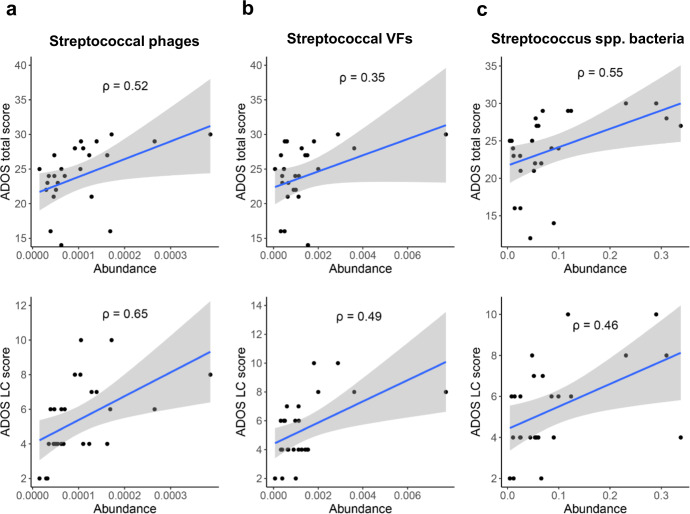
Correlations between oral Streptococcal features and clinical manifestations of ASD. **a**–**c** The correlations of the oral abundance of Streptococcal phages (**a**), Streptococcal VFs (**b**), Streptococcus spp. bacteria (**c**) to the total and LC (Language and Communication domain) score in the ADOS evaluations. All correlations are tested using the Spearman rank test with the rho values labeled on each scatterplot.

To further confirm the association of oral *Streptococcus* and their phages and VFs to ASD pathogenesis, we look into scores of autism diagnostic observation schedule (ADOS) of our cohort. The total score of ADOS is composed of scores of the four domains: (1) language and communication (LC); (2) reciprocal social interactions (RSI); (3) stereotyped behaviors and restricted interests (SBRI); and (4) play, where a higher score indicates more severity in clinical manifestations^47^. First, the abundance of Streptococcal phages shows strong positive correlations to total scores (rho = 0.52) and LC scores (rho = 0.61) in ADOS evaluation (Fig. 6a, Supplementary Table 7). Second, the abundance of Streptococcal VFs also exhibits positive correlations to total ADOS scores (rho = 0.38) and LC scores (rho = 0.45) (Fig. 6b, Supplementary Table 7), as well as the total abundance of *Streptococcus* spp. bacteria to total ADOS scores (rho = 0.54) and LC scores (rho = 0.42) (Fig. 6c, Supplementary Table 7). The consistently strong correlations support the proposal for a potential contribution of oral *Streptococcus* and their phages and VFs in ASD pathogenesis and evident effects on aggravating severity of the disorder. The correlations to other scores of ADOS domains and other clinical manifestations are comparatively weaker, implying a more prominent impairment of language communication from the oral *Streptococcus* (Supplementary Fig. 7, Supplementary Table 7).

Interestingly, whether *Streptococcus* species and their phages, as well as VFs in the gut microbiota, are associated with ASD appears uncertain. The intestinal abundance of Streptococcal phages, VFs, and *Streptococcus* spp. bacteria are weakly or even negatively correlated to the ADOS total score or scores of each domain (Supplementary Fig. 8). The irrelevance of the gut *Streptococcus* species, together with phages and VFs, to the clinical manifestations of ASD, suggests that the association, if any, is confined to the oral microbial community.

We further construct a random forest-based diagnostic model to distinguish ASD from control subjects and utilize the ROC curve to evaluate the accuracy of the models. When using the oral abundance of Streptococcal phages as well as 23 *Streptococcus* species in oral flora as input, the AUC (area under the curve) of the ROC curve reaches 0.913 ± 0.074 (Supplementary Fig. 9a). Among all the 24 features, the abundance of Streptococcal phages is one of the five top features with importance >1, indicating its contribution to the diagnostic model (Supplementary Fig. 9b). These results further support the above observations that the overrepresentation of Streptococcus and its prophages in oral flora is among the most evident features of ASD children deviating from a healthy state.

## Discussions

Our metagenome-wide scrutiny on oral and gut phage communities provides sketches of oral phageome in children, which is previously largely unknown, especially in comparison to that of gut. Streptococcal phages, usually being prophages, are one of the outstanding genetic features of *Streptococcus* species to the extent that almost every strain carries at least one prophage or phage-like chromosomal island integrated into multiple sites, with three to four elements as the most common cases^41^. A considerable range of virulence-associated genes is carried by these prophages and prophage-like elements, and the great number and diversity of the prophage-associated virulence genes indicate that prophages are critical for the survival, propagation, and pathogenesis of *Streptococcus*. Streptococcal phages are also characterized by their broad host range that often covers most of the species and serotypes of *Streptococcus*^41^, serving as the major vector responsible for the rapid dissemination of VFs among the *Streptococcus* populations and a major force shaping the virulence of this bacterial pathogen.

Recently, evidence has emerged that bacterial invasions often profoundly affect brain functions^48^, and virulence factors they carry are also associated with certain brain diseases^49–52^. Interestingly, neurotransmitters can, in turn, modulate the virulence of bacteria^53,54^, forming mutual interplays between the body-residing microbes and the brain. Although these findings are mainly discovered in intestinal microbiota, *Streptococcus spp*., well-known for carrying a wide variety of VFs^43^ and the major oral pathogens that accounted for gingivitis and other chronic periodontal infections^44^ also has the potential of releasing inflammation signals to the brain due to the wide range and the great number of virulence factors they encode. Armed with Streptococcal prophages and VFs, the *Streptococcus* spp. seems to have achieved success and flourishment in the oral flora among ASD subjects, implying an in situ inflammatory status. A recent study has reported increased salivary tumor necrosis factor-alpha levels in ASD children^45^, confirming the aberrant inflammatory status in the oral cavity of ASD. The microbial components and inflammatory signals caused by the over-representation of Streptococcal phages and VFs might be transferred to the brain through the microbiome-mouth-brain axis, similar to the microbiome-gut-brain axis, where the abundant cranial nerves terminals under the oral mucosa may function as an “express line” similar to the vagus nerve^55–59^.

The underlying reasons for the enrichment of *Streptococcus* and their phages in the ASD oral community are still unknown. Many factors, such as genetic background, narrow range of food due to allergies and intolerances, toxicant exposures, and poor oral hygiene, may contribute to the alterations and defects in the digestive system, including oral environment, which change the nutrients and immunity of the oral environment for the microbial inhabitants^60,61^. This intriguing mechanism of how oral microbiome and phages participate in the pathogenesis of ASD deserves further investigation as it may lead to novel diagnostic and therapeutic strategies for the control of the disorders.

The limitation of this study is the small sample size and short-read sequencing platform we used. The small sample size (*n* = 26 in each group) leads to weak statistical power, and we have to be careful with false discoveries. Here, we applied the approach of the quasi-paired cohort for a small sample size and identify the differential features based on multiple lines of evidence. The short-reads sequencing platform we used impedes efficient identification of phage sequences from our metagenome data because effective discovery and accurate abundance evaluation of phage communities rely on the continuity of the assembled contigs. The highly enriched Streptococcal phages in the oral microbiota of ASD subjects and the intriguing Streptococcal phage-associated mechanism of ASD pathogenesis deserve further investigations and validation. By utilizing long-read sequencing techniques, such as the ONT (Oxford Nanopore Technology Ltd.) nanopore platform^62^ or SMRT (single molecular real-time) sequencing with HiFi reads^63^, and larger cohorts, further studies on the oral phage community and the potential mouth-brain axis should provide further information about the role played by oral microbiota and its phages in the development of ASD, paving the way for the prevention and management strategies of this devastating disorders.

We noticed the enrichment of Bifidobacteriaceae-associated phage in the gut of ASD subjects, which is against the common view of the beneficial roles of Bifidobacteriaceae in ASD. Careful investigation reveals that many ASD subjects in our cohort have a history of taking various bifidobacterium species as a supplement, although they ceased at least three months before the enrollment. We are unsure if this may alter the abundance of the Bifidobacteriaceae-associated phage in our ASD subjects. Further studies with careful subject enrollment and detailed history-taking will help clarify the role of gut Bifidobacteriaceae-associated phage in ASD. We expect multiple phages may play roles in the pathogenesis of ASD in addition to Streptococcal phage, and further studies with a larger cohort and long-read sequencing data might help to improve the annotation of phage sequence in the metagenome and discover them all.

Our research has also led to a strong association between oral Streptococcal phages and ASD pathogenesis, providing new evidence for direct microbiome-mouth-brain connections and highlighting the role of bacteriophages in it. The Streptococcal phage constitutes a considerable part of the oral phageome, actively propagating and disseminating VFs within the *Streptococcus* population. The unique feature qualifies this group of phages as an abundant resource of inflammation signals in the oral cavity, which can be transmitted to the brain in a similar manner as the gut-brain axis. The intriguing Streptococcal phage-associated mechanism of ASD pathogenesis deserves further investigations and validation, which might provide valuable clues and open a new avenue for the prevention and management strategies of the disorders.

## Methods

### Sample collection and DNA extraction

We recruited 26 ASD children and 26 gender- and age-matched neurotypical controls from the Autism Research Center of Peking University Health Science Center and surrounding communities. The diagnosis of ASD according to the Diagnostic and Statistical Manual of Mental Disorders (Fifth Edition) criteria was confirmed with the ADOS. Rating or scoring instruments of the Childhood Autism Rating Scale (CARS), Repetitive Behavior Scale-Revised (RBS-R), Autism Behavior Checklist (ABC), and Gastrointestinal Severity Index (GSI) were also performed for ASD children. For the control subjects, medical examinations and parent interviews were performed to exclude anyone with psychiatric disorders and complaints of gastrointestinal symptoms, therefore, Gastrointestinal Severity Index in the control group was not collected. This study has been approved by the Institutional Review Board of Peking University (Ethical Review Document No. IRB00001052-18070). This study group gained written informed consent from the parents/guardians for the collection of oral and stool samples and trial information. We confirmed that all methods were performed in accordance with IRB guidelines and relevant regulations.

Subjects were excluded if they experienced an infection or took psychoactive medications, antibiotics, probiotics, prebiotics, or a special diet three months before enrollment. Professional oral examination and questionnaire were conducted to evaluate the oral condition of each participant. For each subject, we collected at least ~0.5 g of fecal samples and dental plaque samples of more than three sites, usually from the tongue side of the lower anterior teeth, the cheek side of the left and right maxillary molars, the tongue side of the left and right mandibular molars. In most cases, we took the superior gingival plaques to avoid bleeding and contamination of host DNA. Fecal and oral plaque specimens of all participants were collected in parallel for each subject and immediately frozen at −20 °C in sample tubes and stored at −80 °C for subsequent metagenome sequencing. The total DNA of each fecal sample was extracted by using the QIAamp PowerFecal Pro DNA Kit (QIAGEN), while oral specimens were by using the PowerSoil Pro Kit (QIAGEN) for microbial samples of trace amount.

### Metagenome sequencing and annotation

For the total DNA of each oral/fecal sample, paired-end sequencing was performed on the Illumina HiSeq ×10 platform (150 bp ×2). Kneaddata (https://huttenhower.sph.harvard.edu/kneaddata), which integrated the procedure of quality control and decontamination, was used for raw reads to remove ambiguous sequences and adapters, low-quality bases and reads, and reads of human origin. The final clean reads were applied to microbial classification and genome assembly. MetaPhlAn2 (https://huttenhower.sph.harvard.edu/metaphlan2) was used to annotate the microbial taxonomy and calculate the relative abundance of each species with the Chocophlan database. SPAdes (http://cab.spbu.ru/software/spades/) was used for metagenomic assembly. CD-HIT (http://weizhongli-lab.org/cd-hit/) was used to establish the non-redundant reference of contigs based on the threshold of 98% sequence identity over the full length of the shorter sequence for each sample.

### Construction of phage genome database, taxonomy, and host assignment

VIBRANT (https://github.com/AnantharamanLab/VIBRANT) was used to search for the phage and prophage sequences from the non-redundant metagenomic contigs assembled from each sample. The identified phages were then applied to virus taxonomy annotation using vConTACT2 (https://bitbucket.org/MAVERICLab/vcontact2). Only viral contigs with medium or high quality in VIBRANT analysis or clustered with known viruses in vConTACT2 results were retained. CheckV (https://bitbucket.org/berkeleylab/checkv/) was used to further assess the quality and completeness of phage genomes, only phage sequences estimated as high-quality in Checkv_quality/MIUVIG_quality (i.e., >90% genome completeness) and without warning signs were used for the following analysis. For prophages, CheckV was also used for the removal of host contamination to purify the genome sequences of phages. CD-HIT was then used to obtain the non-redundant phage references for the oral/gut phage ensembles as well as the overlapping genome sequences between the two ensembles based on the threshold of sequence identity >95%. The similarity in the gene content of phage communities between the oral and gut samples was calculated as the proportion of shared genes in the total phage genes annotated by VIBRANT.

The host of phages was predicted by two complementary approaches, i.e., clustered with phages of the known host using vConTACT2 or containing CRISPR spacer sequences. For the latter approach, a specific CRISPR spacer reference database was constructed for bacterial reference genomes deposited in NCBI (National Center for Biotechnology Information) using PILER-CR (http://www.drive5.com/pilercr/) and custom python scripts. A bacterial host was assigned to a given phage if its genome contains sequences matching the CRISPR spacer of a bacterium with sequence identity > 93% of the entire spacer length or ≤2 mismatches by using BLAST+ (https://blast.ncbi.nlm.nih.gov/Blast.cgi).

### Quantifying the abundance of virulence factors and phages

To identify the VF genes, we first downloaded all the VF nucleotide sequences from the VF database (VFDB, http://www.mgc.ac.cn/VFs/). Bowtie2 (http://bowtie-bio.sourceforge.net/bowtie2) was used to map clean reads to nucleotide sequences of VF genes for each sample and the relative abundance of each VF was calculated as the proportion of mapped reads in total clean reads. Based on the detailed annotation of VFs, we further grouped them according to their host taxonomy.

To calculate the abundance of phages in each sample, we mapped clean reads to the non-redundant phage genome sequences, and the abundance of each phage was defined as the proportion of mapped reads in the total clean reads of the sample. Specifically, we filtered the mapped reads of phage genomes based on the threshold of MapQ > 30, to reduce the effect of random mapping and the sample sources of phage genomes. The abundance of phages hosted by a specific bacterial taxon was the sum of the abundance of each individual phage whose host is solely assigned to the bacteria. The abundance of phages was also inferred based on the phage genome references deposited in the updated GPD (http://ftp.ebi.ac.uk/pub/databases/metagenomics/genome_sets/gut_phage_database/) by directly mapping clean reads to them using Bowtie2, and the abundance was calculated as the proportion of mapped reads for each phage. The host information of each phage was retrieved from the GPD_metadata.tsv file, then summarized the total abundance of phages hosted by given bacterial taxa. LEfSe was used to identify the differently abundant phages in oral and gut microbiota based on phage abundance profiles in the host-associated genus and family levels.

### Construction of quasi-paired cohort

The quasi-paired cohort was constructed as previously described^7^. Briefly, a high-dimensional space was first constructed where the proportion of each phage represented a dimension, and all subjects were positioned in the space according to their phage profiles. Thus, the samples formed a Euclidean distance-based similarity network. The next step was to find boundary samples, which are critical in defining the boundaries between phenotype groups. Boundary samples are those exhibiting a higher phage profile similarity to samples of an opposite group than to samples in the same group, i.e., longer distance to neighbors than members of an opposite group in the high-dimensional space. To find boundary samples, the similarity of each sample to its nearest k neighbors was first calculated as KNN (the average Euclidean distance to k nearest neighbors), where k was the square root of the sample size. Then, outliers that were too far (KNN > mean KNN + SD) and redundancies that were too near (KNN < mean KNN − SD) to their neighbors were removed to avoid stochastic impacts. Finally, samples of intragroup KNN intergroup KNN were selected as boundary samples for both groups. Each boundary sample was used to construct k sample pairs with its k nearest neighbors of the opposite side, and these pairs were used to constitute a cohort of paired samples where samples of each pair bear a similar species profile (nearest neighbors in the high-dimensional space) but opposite phenotypes. After removing redundant pairs, the quasi-paired cohort was finally reconstructed.

### Random forest model and ROC curve

Random forest model with the abundance of Streptococcal phage and the 23 *Streptococcus* species as input was constructed with the caret package (https://cran.r-project.org/web/packages/caret/) and the randomForest R package (https://cran.r-project.org/web/packages/randomForest/index.html). Then the diagnostic capacity of the Streptococcal phage and the panel of all Streptococcal features were evaluated with AUC and accuracy through the R packages pROC (https://cran.r-project.org/web/packages/pROC/index.html) and ROCR (https://cran.r-project.org/web/packages/ROCR/index.html) in discriminating the ASD from control subjects. The model was trained by 50% of samples and tested with all samples with 1000 times bootstrap replicates.

### Statistics and data visualizations

All statistical analysis was performed by using R software v4.0.3. The Mann–Whitney *U* test (Wilcoxon rank-sum test) was used to compare the average between the ASD and the control groups from the original group cohort in abundance/proportion of species, VF genes, and phages, with FDR < 0.05 as significant. For paired samples in the “quasi-paired cohort”, statistical significance in proportion was tested with a Wilcoxon signed-rank test, with FDR < 0.05 as significant. The correlations of the abundance of the Streptococcal phages, VFs, and the *Streptococcus* bacteria to ASD rating scores were evaluated with Spearman’s rank correlation coefficient. Finally, several R packages were used for data visualization, including ggplot2, ggpubr, gggenes and RColorBrewer. The gene arrow map was drawn using gggenes (https://cran.r-project.org/web/packages/gggenes/index.html), which is an R package based on ggplot2.

## Supplementary information

Supplementary Material

## Data Availability

All data needed to evaluate the conclusions in the paper are present in the paper and/or the Supplementary Materials. The raw metagenome sequencing data reported in this paper have been deposited in the Genome Sequence Archive in BIG Data Center, Beijing Institute of Genomics (BIG), Chinese Academy of Sciences, under accession numbers CRA004105 at http://bigd.big.ac.cn/gsa. Additional data related to this paper may be requested from the authors.
