# Supplementary material for: Implications of oral streptococcal bacteriophages in autism spectrum disorder
Source: NPJ Biofilms Microbiomes. 2022 Nov 18;8:91. doi: 10.1038/s41522-022-00355-3 (PMC9674646; doi:10.1038/s41522-022-00355-3)
Supplement: Supplementary file 1 — Supplementary Material [file 41522_2022_355_MOESM1_ESM.pdf]

Supplementary Materials:

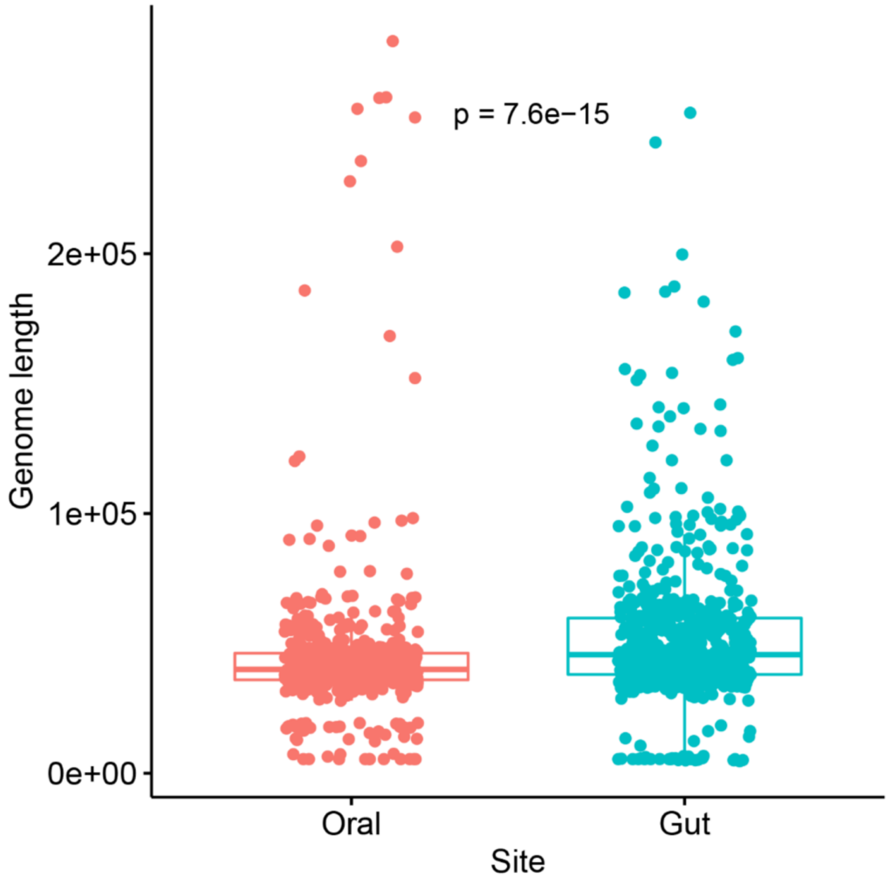

Supplementary Figure 1. Comparison of the genome length of oral and gut phages. Statistical significance is tested with Mann-Whitney U-test.

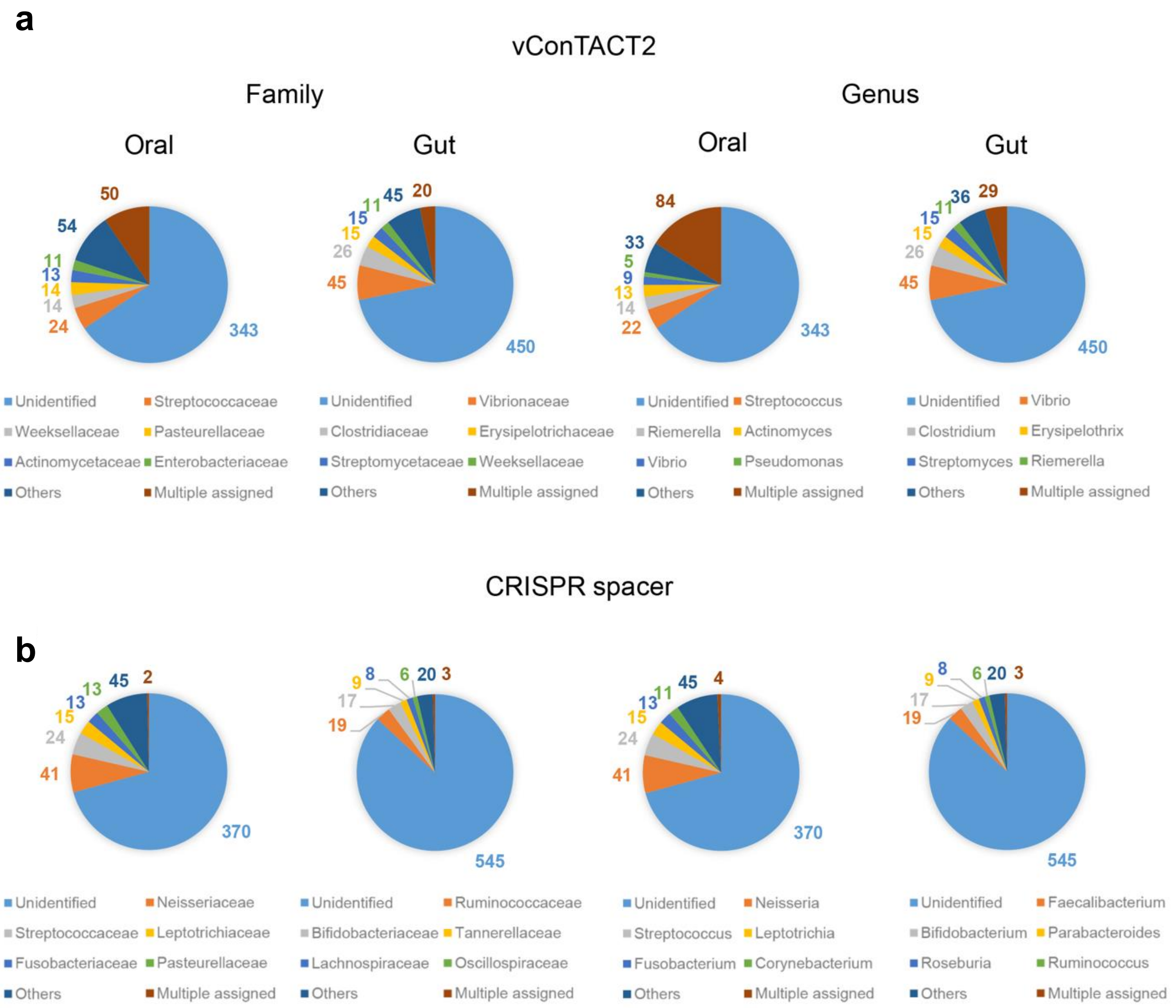

Supplementary Figure 2. Host prediction for phages using complementary approaches. (**a**) Host prediction by vConTACT2 clustering to phages with known host in the family (left) and genus (right) levels. (**b**) Host prediction by CRISPR spacer sequences matching in the family (left) and genus (right) levels.

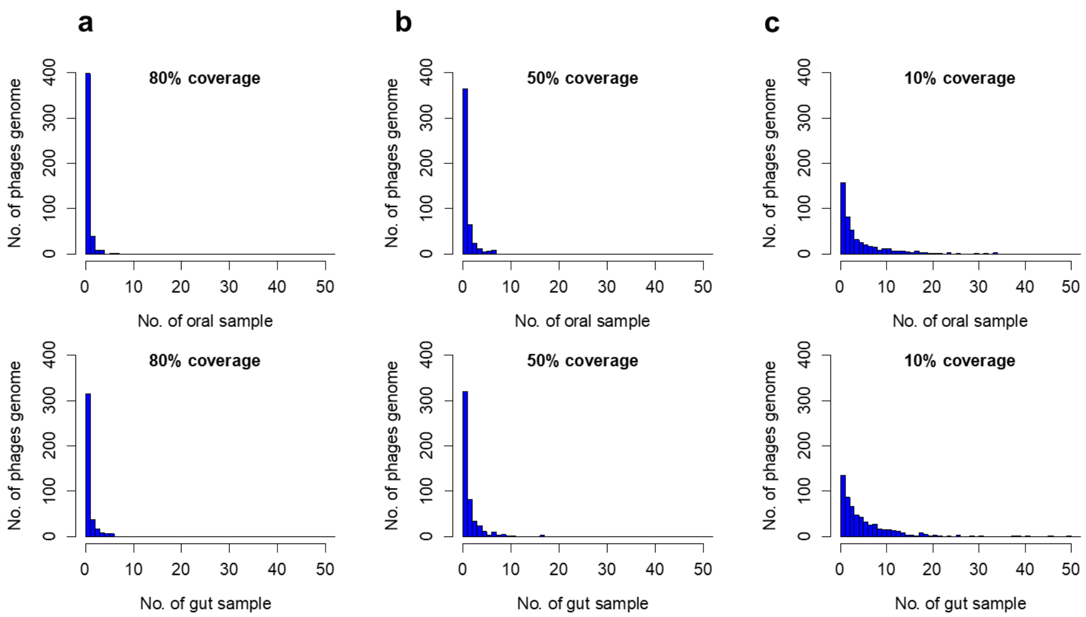

Supplementary Figure 3. The number of phages shared among individuals using cutoffs of >80%(A), >50%(B), and >10%(C) genome coverage in oral (top) and gut (bottom) samples.

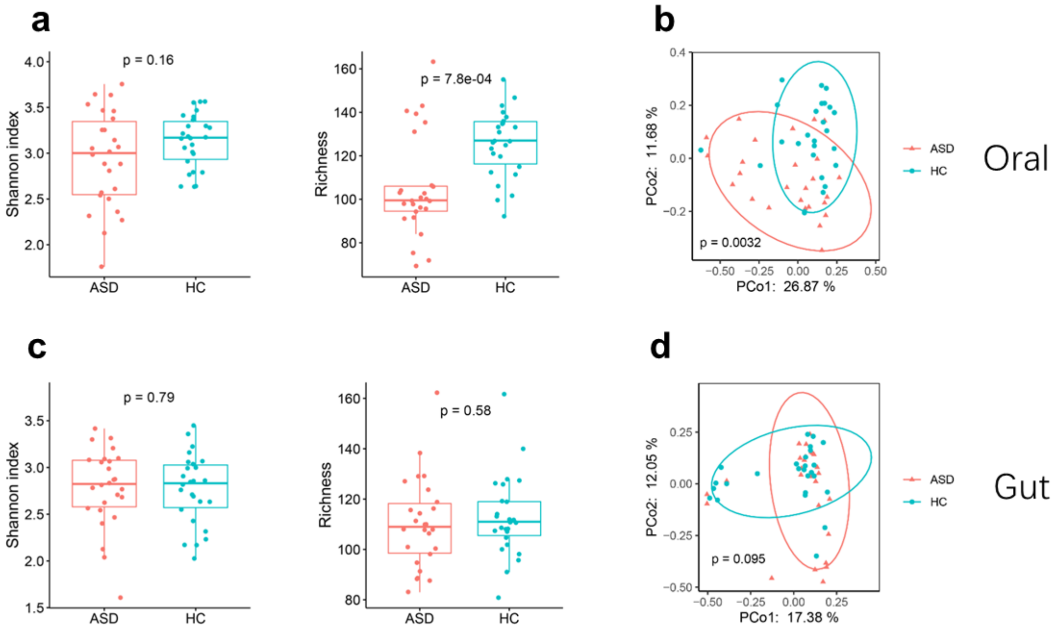

Supplementary Figure 4. Comparison of diversity of bacterial microbiome between ASD and HC groups. **(a)** Shannon index and richness of oral microbiota in the two groups tested by Mann-Whitney U test. **(b)** The principal coordinate analysis of species profile of the oral samples from both groups. The distances are tested by PERMANOVA. **(c)** Shannon index and richness of gut microbiota in the two groups tested by Mann-Whitney U test. **(d)** The principal coordinate analysis of species profile of the gut samples from both groups. The distances are tested by PERMANOVA. In box-whisker plots, the middle horizontal line refers to median value, and width of box is interquartile range (IQR) with 1.5IQR whisker length.

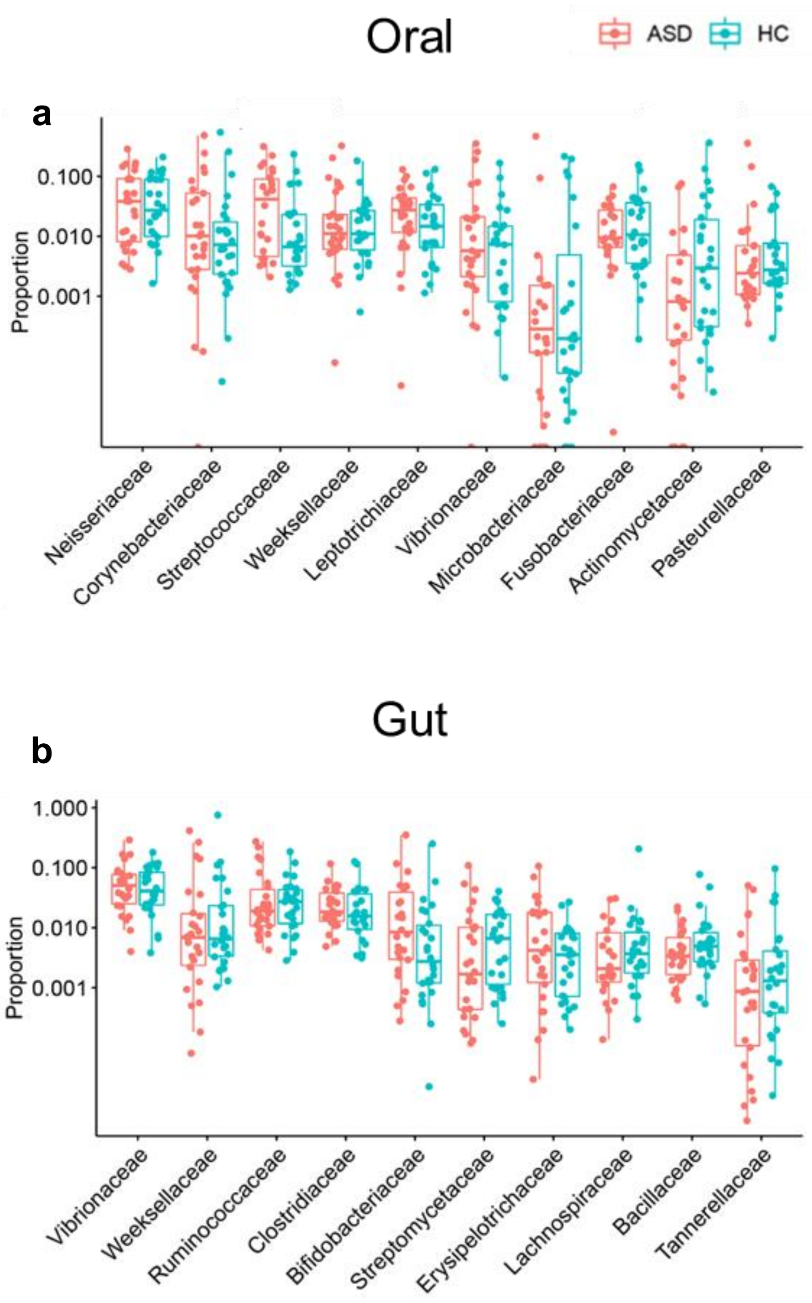

Supplementary Figure 5. The proportion of phages hosted by the major bacterial families. (**a**) The proportion of oral phages hosted by the major bacterial families compared between ASD and control (HC) groups. (**b**) The proportion of gut phages hosted by the major bacterial families compared between ASD and control (HC) groups. The Mann-Whitney U-test was used to detect significant differences, and FDR< 0.05 was taken as statistically significant. In box-whisker plots, the middle horizontal line refers to median value, and width of box is interquartile range (IQR) with 1.5IQR whisker length.

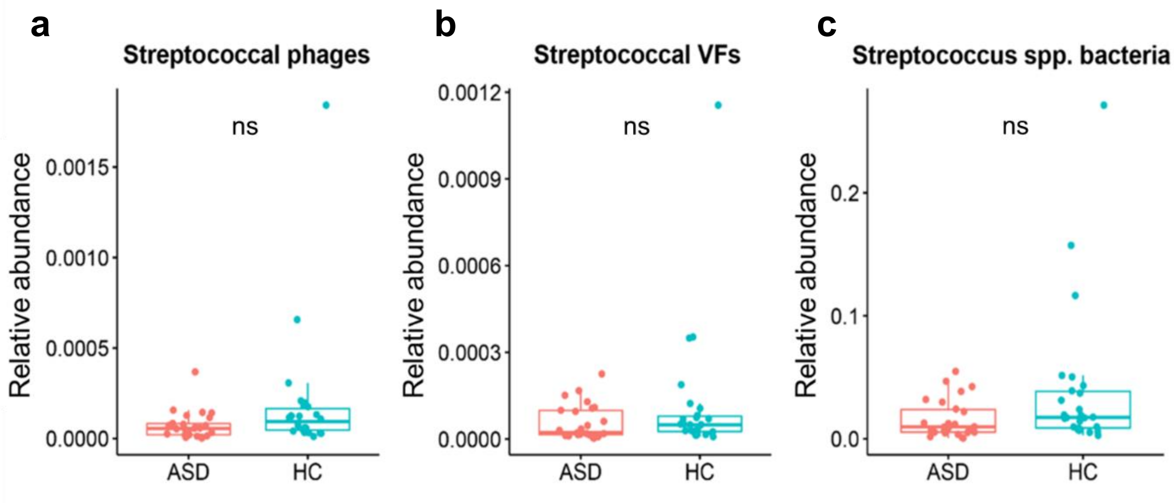

Supplementary Figure 6. The relative abundance of Streptococcal phages, VFs, and Streptococcus spp. bacterial in the gut microbiota. The abundance of Streptococcal phages (**a**), VFs (**b**), and Streptococcus spp. bacteria (**c**) in the gut microbiota compared between the ASD and HC groups. The Mann-Whitney U-test was used to detect significant differences, and FDR < 0.05 was taken as statistically significant. In box-whisker plots, the middle horizontal line refers to median value, and width of box is interquartile range (IQR) with 1.5IQR whisker length.

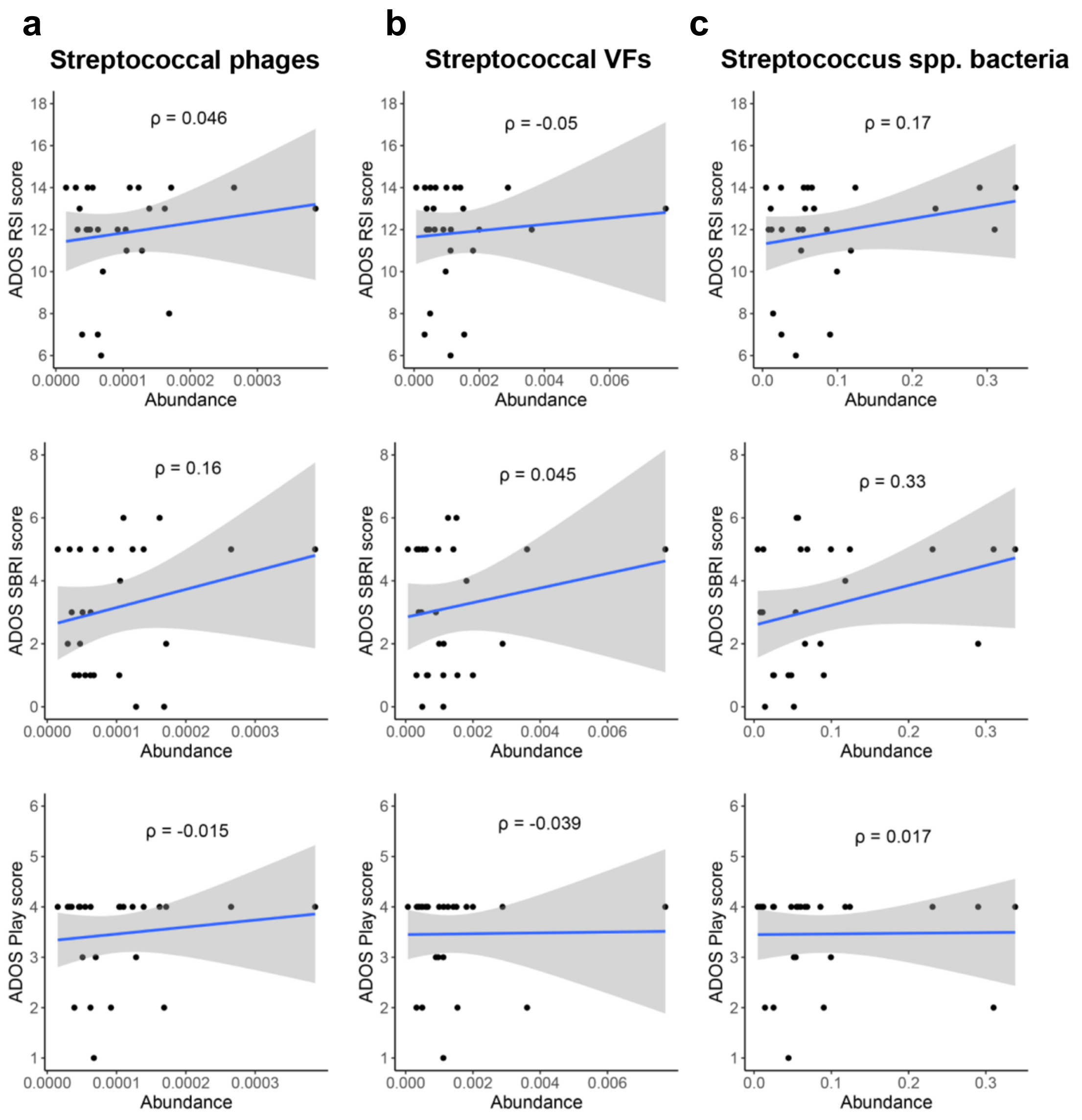

Supplementary Figure 7. The correlation of oral abundance of Streptococcal features to the scores in ADOS evaluation. The correlation of the abundance of Streptococcal phages (**a**), Streptococcal VFs (**b**), and Streptococcus spp. bacteria (**c**) to the RSI (Reciprocal Social Interaction, top), SBRI (Stereotyped Behaviors and Restricted Interests, medium), and Play scores (bottom) in ADOS evaluation. The Rho values of the Spearman rank test of each correlation are labeled in corresponding scatter plots.

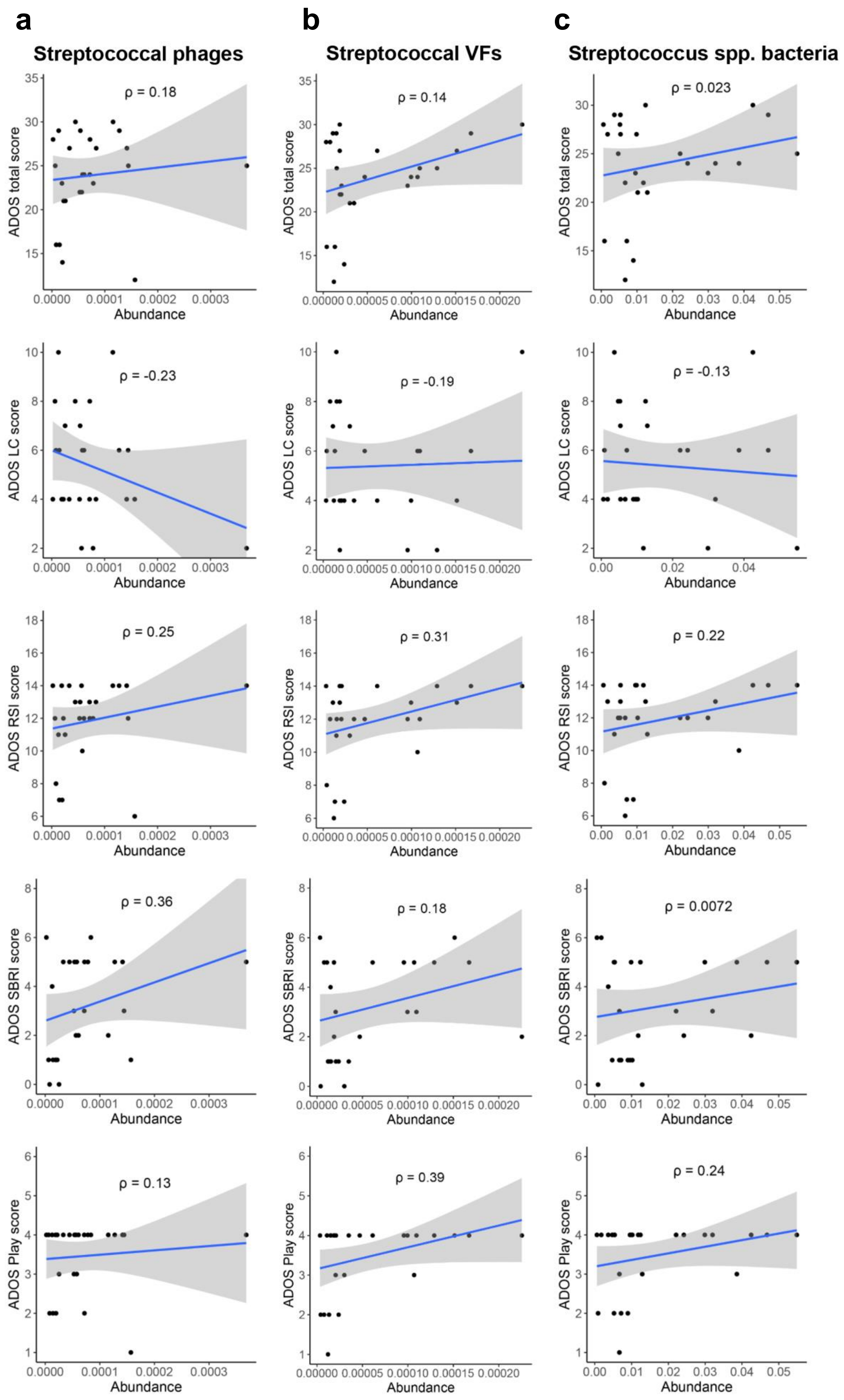

a
Streptococcal phages
b
Streptococcal VFs
c
Streptococcus spp. bacteria
ρ = 0.18
ρ = 0.14
ρ = 0.023
ρ = -0.23
ρ = -0.19
ρ = -0.13
ρ = 0.25
ρ = 0.31
ρ = 0.22
ρ = 0.36
ρ = 0.18
ρ = 0.0072
ρ = 0.13
ρ = 0.39
ρ = 0.24
ADOS total score
ADOS LC score
ADOS RSI score
ADOS SBRI score
ADOS Play score
Abundance

Supplementary Figure 8. The correlation of gut abundance of Streptococcal features to the clinical manifestations of ASD. The correlation of the abundance of Streptococcal phages (**a**), Streptococcal VFs (**b**), and Streptococcus spp. bacteria (**c**) to the scores of each domain and their sum. Scatter plots from top to bottom exhibit correlations to total score, scores of LC (Language and Communication), RSI (Reciprocal Social Interaction), SBRI (Stereotyped Behaviors and Restricted Interests), and Play domains in ADOS evaluation. The Rho values of the Spearman rank test for each correlation were labeled in each plot.

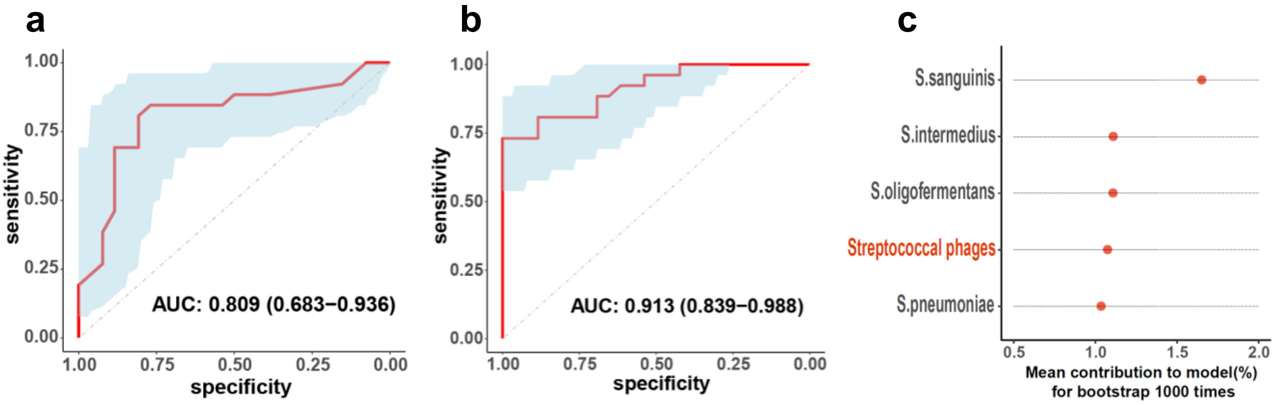

Supplementary Figure 9. The ROC curve based on the oral Streptococcal phage abundance **(a)** and adding the abundance of each of 23 Streptococcus species in oral flora **(b)** in distinguishing ASD from control subjects. **(c)**. The contribution of features to the diagnostic model with importance >1.

Supplementary Tables:

Supplementary Table 1. Sequencing data information

		ASD-gut	ASD-oral	HC-gut	HC-oral
raw reads count	mean	42,769,163	30,393,125	53,266,618	50,395,240
	sd	19,083,796	7,662,757	13,380,959	11,276,080
clean reads count	mean	39,029,978	26,434,926	48,300,487	42,504,252
	sd	17,070,529	6,822,952	12,061,013	9,872,256

Supplementary Table 2. Phage genome annotation information in gut phage ensemble

Phage ID	Genome length	Provirus	Taxonomy	Host bacteria (genus)	Host bacteria (family)
NODE_429_length_46997_cov_28.121405	46997	No	NA	NA	NA
NODE_286_length_61232_cov_13.873416	61232	No	Siphoviridae	Riemerella	Weeksellaceae
NODE_151_length_89206_cov_50.561822_fragment_1_1	48223	Yes	NA	Bacteroides	Bacteroidaceae
NODE_577_length_38286_cov_18.312809	38286	No	Siphoviridae	Erysipelothrix	Erysipelotrichaceae
NODE_74_length_121384_cov_41.006849_fragment_3	37501	Yes	NA	NA	NA
NODE_157_length_87335_cov_12.711068	87335	No	NA	NA	NA
NODE_491_length_42595_cov_18.463235	42595	No	NA	NA	NA
NODE_4413_length_5441_cov_750.485704	5441	No	Microviridae	Enterobacteria,Escherichia	Enterobacteriaceae
NODE_205_length_74161_cov_14.440072_1	71036	Yes	NA	NA	NA
NODE_834_length_29002_cov_38.220679	29002	No	NA	NA	NA
NODE_88_length_181531_cov_11.221065	181531	No	NA	NA	NA
NODE_647_length_67545_cov_47.761876_fragment_1_1	33582	Yes	NA	NA	NA
NODE_518_length_77331_cov_24.501902_fragment_1_1	63374	Yes	NA	NA	NA
NODE_367_length_96321_cov_9.240635_fragment_1	44273	Yes	Myoviridae	Roseburia,Vibrio	Lachnospiraceae,Vibrionaceae
NODE_1153_length_43341_cov_1941.745876	43341	No	NA	NA	NA
NODE_247_length_117474_cov_43.539282_fragment_1	54230	Yes	NA	NA	NA
NODE_67_length_203296_cov_28.945356_fragment_2_1	32363	Yes	Siphoviridae	Ruminococcus,Streptococcus	Oscillospiraceae,Streptococcaceae
NODE_98_length_176989_cov_20.959878_fragment_1	159133	Yes	NA	Roseburia	Lachnospiraceae
NODE_1458_length_35398_cov_6.591461	35398	No	NA	NA	NA
NODE_3650_length_14099_cov_6.921224	14099	No	NA	NA	NA
NODE_1087_length_45791_cov_13.936418	45791	No	NA	NA	NA
NODE_176_length_139267_cov_15.926472_1	137361	Yes	NA	NA	NA

NODE_1102_length_45224_cov_8.634993	45224	No	NA	NA	NA
NODE_826_length_57131_cov_48.423313_1	45324	Yes	NA	NA	NA
NODE_10079_length_4994_cov_8.518324	4994	No	Microviridae	Bdellovibrio,Chlamydia	Bdellovibrionaceae,Chlamydiaceae
NODE_111_length_170278_cov_25.805314_fragment_2_1	53826	Yes	NA	NA	NA
NODE_360_length_97793_cov_8.819098_fragment_1	46588	Yes	NA	NA	NA
NODE_696_length_64637_cov_141.450033	64637	No	NA	NA	NA
NODE_1136_length_43988_cov_15.196185	43988	No	NA	NA	NA
NODE_1361_length_37424_cov_8.400171	37424	No	NA	NA	NA
NODE_794_length_58717_cov_7.695544	58717	No	NA	NA	NA
NODE_1300_length_38929_cov_10.013196	38929	No	NA	Roseburia	Lachnospiraceae
NODE_81_length_187383_cov_13.919377	187383	No	Unassigned	uncultured~crAssphage	uncultured~crAssphage
NODE_1245_length_40666_cov_6.376770	40666	No	NA	NA	NA
NODE_408_length_90049_cov_36.917561_fragment_1	49422	Yes	NA	NA	NA
NODE_1030_length_48004_cov_35.284719	48004	No	NA	NA	NA
NODE_1112_length_45024_cov_6.711801	45024	No	NA	NA	NA
NODE_1071_length_46471_cov_8.814654	46471	No	Myoviridae	Roseburia,Vibrio	Lachnospiraceae,Vibrionaceae
NODE_48_length_227109_cov_10.140341_fragment_3	38186	Yes	NA	NA	NA
NODE_901_length_53846_cov_75.755294_fragment_1	37142	Yes	NA	NA	NA
NODE_602_length_54996_cov_8.866912	54996	No	NA	NA	NA
NODE_183_length_116462_cov_20.672966_fragment_1_1	63072	Yes	NA	NA	NA
NODE_670_length_50159_cov_19.786105	50159	No	NA	Clostridium	Clostridiaceae
NODE_833_length_41374_cov_9.926281	41374	No	NA	NA	NA
NODE_203_length_110700_cov_19.542591_fragment_1_1	42744	Yes	NA	NA	NA
NODE_313_length_85942_cov_12.110948	85942	No	NA	NA	NA
NODE_788_length_43394_cov_783.910150	43394	No	NA	NA	NA

NODE_519_length_62892_cov_47.076611	62892	No	NA	NA	NA
NODE_23_length_265651_cov_9.783144_fragment_2_1	40183	Yes	NA	NA	NA
NODE_268_length_95719_cov_80.994962_fragment_1	55231	Yes	Myoviridae	Vibrio	Vibrionaceae
NODE_963_length_36789_cov_10.195269	36789	No	NA	Bifidobacterium	Bifidobacteriaceae
NODE_165_length_120491_cov_33.916686	120491	No	NA	NA	NA
NODE_100_length_147677_cov_36.452636_fragment_1	49324	Yes	NA	NA	NA
NODE_866_length_40013_cov_7.391886	40013	No	NA	NA	NA
NODE_94_length_153384_cov_52.495373_fragment_1_1	35593	Yes	NA	Ruminococcus	Oscillospiraceae
NODE_647_length_51467_cov_83.322823_fragment_1	32470	Yes	NA	NA	NA
NODE_570_length_57406_cov_12.299698	57406	No	Siphoviridae	Streptomyces	Streptomycetaceae
NODE_842_length_41145_cov_18.183767	41145	No	Myoviridae,Unassigned	Clostridium,Streptococcus,Thermus	Clostridiaceae,Streptococcaceae,Thermaceae
NODE_14_length_300208_cov_36.445993_fragment_3	54450	Yes	NA	Parabacteroides	Tannerellaceae
NODE_255_length_98263_cov_268.638400	98263	No	Unassigned	uncultured~crAssphage	uncultured~crAssphage
NODE_822_length_41709_cov_37.237624	41709	No	NA	NA	NA
NODE_477_length_66394_cov_21.892235_fragment_1_1	49764	Yes	NA	NA	NA
NODE_604_length_54969_cov_45.579288	54969	No	NA	NA	NA
NODE_110_length_141230_cov_10.816271_fragment_2_1	55343	Yes	NA	NA	NA
NODE_262_length_80786_cov_92.077987_fragment_1_1	66960	Yes	NA	NA	NA
NODE_1534_length_18411_cov_18.114210	18411	No	Podoviridae	Bifidobacterium,Actinomyces	Bifidobacteriaceae,Actinomycetaceae
NODE_683_length_39845_cov_15.653179	39845	No	NA	NA	NA
NODE_506_length_50968_cov_16.518021_fragment_2	28741	Yes	NA	NA	NA
NODE_6755_length_4660_cov_419.464278	4660	No	Microviridae	Parabacteroides	Tannerellaceae
NODE_229_length_86461_cov_15.189385	86461	No	NA	NA	NA
NODE_401_length_61041_cov_18.034057_fragment_1	49871	Yes	NA	NA	NA

NODE_25_length_213345_cov_10.100070_fragment_1	199712	Yes	NA	NA	NA
NODE_392_length_41560_cov_14.522901	41560	No	NA	NA	NA
NODE_331_length_48039_cov_28.568981	48039	No	Myoviridae	Vibrio	Vibrionaceae
NODE_415_length_40025_cov_12.945159	40025	No	NA	NA	NA
NODE_240_length_61073_cov_8.834442	61073	No	NA	Faecalibacterium	Ruminococcaceae
NODE_257_length_58449_cov_10.720862	58449	No	NA	NA	NA
NODE_297_length_52315_cov_97.692652_1	49361	Yes	Myoviridae	Vibrio	Vibrionaceae
NODE_252_length_59378_cov_58.347133_fragment_1	37762	Yes	NA	Bifidobacterium	Bifidobacteriaceae
NODE_387_length_41653_cov_14.480889	41653	No	NA	NA	NA
NODE_44_length_176417_cov_12.879844_1	170086	Yes	NA	NA	NA
NODE_188_length_73409_cov_312.365733	73409	No	NA	Faecalibacterium	Ruminococcaceae
NODE_512_length_50458_cov_214.304783	50458	No	NA	NA	NA
NODE_252_length_87189_cov_19.231310_1	71039	Yes	NA	NA	NA
NODE_646_length_42992_cov_12.642686	42992	No	NA	NA	NA
NODE_3_length_412210_cov_106.228023_fragment_3	155578	Yes	NA	NA	NA
NODE_61_length_169704_cov_55.510460_fragment_1	37916	Yes	Siphoviridae	Clostridium	Clostridiaceae
NODE_330_length_72380_cov_110.705247_fragment_2	40095	Yes	Myoviridae,Unassigned	Clostridium,Streptococcus,Thermus	Clostridiaceae,Streptococcaceae,Thermaceae
NODE_462_length_53864_cov_6.036146_1	51571	Yes	NA	NA	NA
NODE_3943_length_5441_cov_143.533049	5441	No	Microviridae	Enterobacteria,Escherichia	Enterobacteriaceae
NODE_235_length_91922_cov_307.424037_fragment_1	60343	Yes	Siphoviridae	Riemerella	Weeksellaceae
NODE_87_length_144285_cov_27.554982_fragment_1	41025	Yes	Siphoviridae	Bacillus,Enterococcus	Bacillaceae,Enterococcaceae
NODE_39_length_207780_cov_290.277805_fragment_1_1	65791	Yes	NA	NA	NA
NODE_43_length_202159_cov_18.844654_fragment_1	58588	Yes	Siphoviridae	Streptomyces	Streptomycetaceae
NODE_1_length_661537_cov_357.787184_fragment_10_1	61937	Yes	Siphoviridae	Riemerella	Weeksellaceae

NODE_306_length_77101_cov_10.303520_fragment_1_1	41953	Yes	NA	NA	NA
NODE_453_length_55097_cov_19.252298	55097	No	NA	NA	NA
NODE_594_length_34950_cov_11.008798	34950	No	Siphoviridae	Clostridium	Clostridiaceae
NODE_246_length_67414_cov_6.709764	67414	No	Siphoviridae	Arthrobacter	Micrococcaceae
NODE_274_length_63858_cov_7.787298_fragment_1_1	41433	Yes	NA	Neisseria	Neisseriaceae
NODE_564_length_37071_cov_9.906014	37071	No	NA	NA	NA
NODE_361_length_52531_cov_8.477609_1	50208	Yes	NA	NA	NA
NODE_540_length_38654_cov_112.766859	38654	No	NA	NA	NA
NODE_341_length_55677_cov_5.776941_1	36779	Yes	Siphoviridae	Clostridium	Clostridiaceae
NODE_81_length_137785_cov_41.691999_fragment_1	30826	Yes	Myoviridae	Edwardsiella,Burkholderia,Enterobacteria,Erwinia,Escherichia,Mannheimia,Pseudomonas,Ralstonia,Salmonella,Stenotrophomonas,Yersinia	Enterobacteriaceae,Burkholderiaceae,Erwiniaceae,Pasteurellaceae,Pseudomonadaceae,Xanthomonadaceae,Yersiniaceae
NODE_467_length_43077_cov_17.554832	43077	No	NA	NA	NA
NODE_296_length_61264_cov_93.288013_fragment_1_1	43251	Yes	Myoviridae	Vibrio	Vibrionaceae
NODE_167_length_90789_cov_97.330571_fragment_2	34522	Yes	NA	NA	NA
NODE_496_length_41453_cov_28.980023_fragment_1	32907	Yes	NA	NA	NA
NODE_947_length_36133_cov_12.775514	36133	No	NA	Bifidobacterium	Bifidobacteriaceae
NODE_327_length_79241_cov_13.144394_fragment_1_1	64149	Yes	NA	Faecalibacterium	Ruminococcaceae
NODE_408_length_68217_cov_11.192585	68217	No	NA	NA	NA
NODE_280_length_86610_cov_31.262065	86610	No	NA	NA	NA
NODE_299_length_83893_cov_8.928326_fragment_2	60086	Yes	NA	NA	NA
NODE_884_length_37930_cov_16.898376	37930	No	NA	NA	NA
NODE_324_length_80049_cov_27.115434_fragment_1	54142	Yes	Myoviridae	Vibrio	Vibrionaceae
NODE_144_length_126813_cov_31.341359_fragment_2	66468	Yes	NA	NA	NA

NODE_261_length_90394_cov_11.319961	90394	No	Myoviridae	Bacillus	Bacillaceae
NODE_237_length_95502_cov_16.261056_fragment_2_1	32864	Yes	NA	NA	NA
NODE_1009_length_34138_cov_11.094358	34138	No	NA	NA	NA
NODE_201_length_105472_cov_13.926445_1	95706	Yes	NA	NA	NA
NODE_622_length_49850_cov_11.870409	49850	No	Myoviridae	Vibrio	Vibrionaceae
NODE_679_length_46107_cov_22.470685	46107	No	Myoviridae	Vibrio	Vibrionaceae
NODE_1716_length_16250_cov_295.546581	16250	No	NA	NA	NA
NODE_420_length_40798_cov_18.268905_fragment_1	31342	Yes	Siphoviridae	Paenibacillus	Paenibacillaceae
NODE_95_length_85306_cov_135.716156	85306	No	NA	NA	NA
NODE_11_length_185012_cov_15.072471	185012	No	NA	NA	NA
NODE_495_length_37270_cov_16.876394	37270	No	NA	NA	NA
NODE_384_length_42989_cov_10.650184_1	40565	Yes	NA	NA	NA
NODE_73_length_95140_cov_14.415155	95140	No	Unassigned	uncultured~crAssphage	uncultured~crAssphage
NODE_113_length_81237_cov_471.754662	81237	No	NA	NA	NA
NODE_127_length_118849_cov_26.252067_fragment_2_1	56136	Yes	Siphoviridae	Parabacteroides,Riemerella	Tannerellaceae,Weeksellaceae
NODE_307_length_66166_cov_8.514710_1	61674	Yes	NA	NA	NA
NODE_584_length_37085_cov_21.373508	37085	No	NA	NA	NA
NODE_348_length_59240_cov_19.875543	59240	No	NA	Faecalibacterium	Ruminococcaceae
NODE_193_length_92708_cov_10.154080_fragment_1	52508	Yes	NA	NA	NA
NODE_581_length_37334_cov_19.563964	37334	No	NA	NA	NA
NODE_88_length_148139_cov_16.821041_fragment_1_1	38053	Yes	NA	NA	NA
NODE_5_length_476085_cov_103.627261_fragment_9	33016	Yes	NA	NA	NA
NODE_528_length_41394_cov_8.880428	41394	No	Siphoviridae	Clostridioides,Peptoclostridium,Clostridium	Peptostreptococcaceae,Clostridiaceae
NODE_52_length_183433_cov_35.087715_fragment_2	33412	Yes	NA	NA	NA

NODE_501_length_43178_cov_7.158013	43178	No	NA	NA	NA
NODE_524_length_41570_cov_11.551584_fragment_1	38265	Yes	NA	Bifidobacterium	Bifidobacteriaceae
NODE_150_length_84430_cov_8.191716_fragment_1_1	53312	Yes	NA	NA	NA
NODE_4508_length_5441_cov_1028.350910	5441	No	Microviridae	Enterobacteria,Escherichia	Enterobacteriaceae
NODE_495_length_36016_cov_14.875115	36016	No	NA	NA	NA
NODE_123_length_92686_cov_41.114972_fragment_1_1	33448	Yes	NA	NA	NA
NODE_427_length_40054_cov_10.068777	40054	No	NA	NA	NA
NODE_531_length_34197_cov_6.956124	34197	No	NA	Bifidobacterium	Bifidobacteriaceae
NODE_146_length_85103_cov_14.115676_1	60666	Yes	Siphoviridae	Streptomyces	Streptomycetaceae
NODE_178_length_82593_cov_18.717573_fragment_1	48245	Yes	Myoviridae	Eubacterium,Vibrio	Eubacteriaceae,Vibrionaceae
NODE_5736_length_6701_cov_7.029040	6701	No	Microviridae	NA	NA
NODE_2547_length_13458_cov_20.390510	13458	No	NA	NA	NA
NODE_217_length_73264_cov_11.463372_1	58408	Yes	Myoviridae	Vibrio	Vibrionaceae
NODE_2_length_568600_cov_28.234694_fragment_1	43266	Yes	Myoviridae	Vibrio	Vibrionaceae
NODE_330_length_58578_cov_210.192933	58578	No	NA	NA	NA
NODE_3_length_391199_cov_11.802694_fragment_2	38039	Yes	NA	NA	NA
NODE_194_length_78008_cov_26.741254_fragment_1	43345	Yes	Siphoviridae	Clostridium	Clostridiaceae
NODE_149_length_88913_cov_15.656857_fragment_1	70954	Yes	NA	NA	NA
NODE_1_length_657160_cov_25.633005_fragment_2_1	57674	Yes	NA	NA	NA
NODE_363_length_56283_cov_22.997386	56283	No	NA	Clostridium	Clostridiaceae
NODE_379_length_54482_cov_10.252081_fragment_1_1	41380	Yes	Myoviridae	Vibrio	Vibrionaceae
NODE_117_length_101772_cov_87.589449	101772	No	Unassigned	Cellulophaga	Flavobacteriaceae
NODE_491_length_45308_cov_192.530042	45308	No	NA	NA	NA
NODE_419_length_50989_cov_17.359387_1	45093	Yes	Myoviridae	Vibrio	Vibrionaceae
NODE_563_length_41407_cov_25.538523	41407	No	NA	NA	NA

NODE_296_length_61747_cov_16.199750	61747	No	NA	NA	NA
NODE_123_length_98667_cov_56.559678	98667	No	Unassigned	uncultured~crAssphage	uncultured~crAssphage
NODE_812_length_32298_cov_9.312502	32298	No	NA	NA	NA
NODE_4_length_349514_cov_18.774483_fragment_3	38927	Yes	NA	Ruminococcus	Oscillospiraceae
NODE_544_length_46374_cov_6.919493	46374	No	NA	NA	NA
NODE_603_length_43126_cov_10.426226	43126	No	NA	Roseburia	Lachnospiraceae
NODE_62_length_139915_cov_12.952124_fragment_3_1	36368	Yes	NA	Ruminococcus	Oscillospiraceae
NODE_4140_length_5819_cov_19.310548	5819	No	Microviridae	NA	NA
NODE_154_length_98980_cov_10.639626_fragment_1	38602	Yes	NA	Bifidobacterium	Bifidobacteriaceae
NODE_4807_length_5096_cov_21.785558	5096	No	Microviridae	Bdellovibrio,Chlamydia	Bdellovibrionaceae,Chlamydiaceae
NODE_204_length_85418_cov_8.292621	85418	No	NA	NA	NA
NODE_327_length_64872_cov_14.053551_fragment_1	49128	Yes	NA	NA	NA
NODE_4_length_340877_cov_14.287883_fragment_2	56839	Yes	NA	NA	NA
NODE_717_length_37264_cov_80.995028	37264	No	Siphoviridae	Erysipelothrix	Erysipelotrichaceae
NODE_253_length_78113_cov_11.378296_fragment_1	35538	Yes	NA	Bifidobacterium	Bifidobacteriaceae
NODE_55_length_145830_cov_13.484562_fragment_1	63345	Yes	Siphoviridae	Riemerella	Weeksellaceae
NODE_632_length_41292_cov_9.507918	41292	No	NA	NA	NA
NODE_13_length_235137_cov_135.558733_fragment_4_1	70309	Yes	NA	NA	NA
NODE_242_length_87089_cov_36.794506	87089	No	NA	NA	NA
NODE_259_length_84827_cov_73.192693	84827	No	NA	NA	NA
NODE_7_length_371390_cov_18.552164_fragment_2	86974	Yes	NA	NA	NA
NODE_24_length_259061_cov_42.253998_fragment_2	43072	Yes	NA	NA	NA
NODE_756_length_35875_cov_12.864405	35875	No	Siphoviridae	Bacillus,Croceibacter,Enterococcus	Bacillaceae,Flavobacteriaceae,Enterococcaceae
NODE_4686_length_5441_cov_627.662273	5441	No	Microviridae	Enterobacteria,Escherichia	Enterobacteriaceae

NODE_501_length_51370_cov_5.869921_fragment_1	47326	Yes	Podoviridae	Bordetella,Burkholderia,Pseudomonas,Xanthomonas	Alcaligenaceae,Burkholderiaceae,Pseudomonadaceae,Xanthomonadaceae
NODE_211_length_93467_cov_6.614739_fragment_1_1	38506	Yes	Siphoviridae	Bacillus,Croceibacter,Enterococcus	Bacillaceae,Flavobacteriaceae,Enterococcaceae
NODE_144_length_121115_cov_114.021023_fragment_2	77288	Yes	NA	NA	NA
NODE_216_length_92315_cov_134.169792_fragment_1	81779	Yes	NA	NA	NA
NODE_139_length_102567_cov_21.279333_fragment_1	71933	Yes	NA	NA	NA
NODE_298_length_68858_cov_28.664012_fragment_1	45497	Yes	NA	NA	NA
NODE_805_length_33666_cov_8.044837	33666	No	Siphoviridae	Clostridium	Clostridiaceae
NODE_347_length_61654_cov_12.763551_fragment_1	35472	Yes	NA	Bifidobacterium	Bifidobacteriaceae
NODE_179_length_92443_cov_6.772146_fragment_1_1	39544	Yes	NA	NA	NA
NODE_617_length_41407_cov_12.871421	41407	No	NA	NA	NA
NODE_422_length_54512_cov_77.636833	54512	No	NA	Parabacteroides	Tannerellaceae
NODE_315_length_65956_cov_5.031654_fragment_1_1	37684	Yes	NA	NA	NA
NODE_246_length_76716_cov_109.551284_fragment_1	63209	Yes	Siphoviridae	Riemerella	Weeksellaceae
NODE_103_length_112851_cov_27.504335_fragment_1_1	64884	Yes	NA	NA	NA
NODE_370_length_59336_cov_23.117592	59336	No	Siphoviridae	Streptomyces	Streptomycetaceae
NODE_320_length_65344_cov_74.012805	65344	No	NA	NA	NA
NODE_474_length_49472_cov_7.178522_fragment_1	38024	Yes	Siphoviridae	Erysipelothrix	Erysipelotrichaceae
NODE_414_length_55640_cov_19.156661_1	46235	Yes	NA	NA	NA
NODE_324_length_64762_cov_49.434404	64762	No	NA	NA	NA
NODE_7_length_283655_cov_14.839291_fragment_1_1	44883	Yes	NA	NA	NA
NODE_273_length_59339_cov_21.844056	59339	No	Siphoviridae	Streptomyces	Streptomycetaceae
NODE_13_length_284397_cov_320.309986_fragment_2	76707	Yes	NA	NA	NA
NODE_357_length_50543_cov_9.654968_1	44366	Yes	NA	NA	NA

NODE_405_length_45108_cov_14.641666	45108	No	NA	NA	NA
NODE_345_length_51751_cov_8.790371	51751	No	NA	NA	NA
NODE_50_length_168767_cov_35.523916_fragment_1	140886	Yes	NA	NA	NA
NODE_497_length_36823_cov_44.917319	36823	No	NA	NA	NA
NODE_303_length_56014_cov_97.169017	56014	No	NA	Faecalibacterium	Ruminococcaceae
NODE_671_length_54937_cov_11.305200_fragment_1	34042	Yes	Siphoviridae	Clostridium	Clostridiaceae
NODE_516_length_65769_cov_62.461195	65769	No	NA	NA	NA
NODE_341_length_82837_cov_16.883223_fragment_1	62201	Yes	NA	NA	NA
NODE_216_length_104884_cov_32.643934_fragment_1_1	64486	Yes	NA	NA	NA
NODE_299_length_90095_cov_8.693259_fragment_1_1	35003	Yes	Siphoviridae	Clostridium	Clostridiaceae
NODE_207_length_108240_cov_16.416749_fragment_1_1	33711	Yes	NA	NA	NA
NODE_122_length_135918_cov_31.964700_fragment_3	62346	Yes	NA	NA	NA
NODE_723_length_51525_cov_10.219468_fragment_1	37844	Yes	NA	NA	NA
NODE_117_length_137109_cov_241.405570_fragment_2	73863	Yes	NA	NA	NA
NODE_969_length_40852_cov_48.138981	40852	No	NA	NA	NA
NODE_828_length_46364_cov_7.028677_fragment_1	36876	Yes	NA	NA	NA
NODE_34_length_222664_cov_23.755940_fragment_2	48205	Yes	NA	Eubacterium	Eubacteriaceae
NODE_30_length_226376_cov_34.070670_fragment_2	57219	Yes	Siphoviridae	Clostridium	Clostridiaceae
NODE_936_length_42055_cov_44.533024	42055	No	Siphoviridae	Clostridium	Clostridiaceae
NODE_303_length_89573_cov_14.823209_fragment_1_1	57741	Yes	NA	NA	NA
NODE_295_length_71366_cov_20.263900_fragment_1	55536	Yes	NA	Faecalibacterium	Ruminococcaceae
NODE_159_length_99161_cov_15.941204	99161	No	Unassigned	uncultured~crAssphage	uncultured~crAssphage
NODE_723_length_36726_cov_15.256551	36726	No	NA	NA	NA
NODE_242_length_81007_cov_30.506634_1	61669	Yes	Siphoviridae	Streptomyces	Streptomycetaceae
NODE_151_length_103184_cov_8.956792_fragment_2	50079	Yes	NA	NA	NA

NODE_6347_length_5816_cov_8.815831	5816	No	Microviridae	NA	NA
NODE_1003_length_39510_cov_11.650463	39510	No	Siphoviridae	Clostridium	Clostridiaceae
NODE_149_length_113717_cov_22.301499	113717	No	NA	NA	NA
NODE_1092_length_36880_cov_41.267101	36880	No	NA	NA	NA
NODE_449_length_67897_cov_8.644895_fragment_1	44646	Yes	NA	NA	NA
NODE_940_length_41775_cov_34.196860	41775	No	Myoviridae,Unassigned	Clostridium,Streptococcus,Thermus	Clostridiaceae,Streptococcaceae,Thermaceae
NODE_153_length_112773_cov_9.456892_fragment_1_1	38055	Yes	NA	NA	NA
NODE_60_length_161947_cov_131.188095_fragment_1_1	47885	Yes	NA	NA	NA
NODE_1033_length_38588_cov_8.631044	38588	No	NA	Bifidobacterium	Bifidobacteriaceae
NODE_31_length_214657_cov_11.384251_fragment_1	39143	Yes	NA	NA	NA
NODE_619_length_56578_cov_9.593458_fragment_1_1	44948	Yes	Myoviridae	Vibrio	Vibrionaceae
NODE_501_length_63518_cov_55.749413	63518	No	NA	NA	NA
NODE_644_length_54697_cov_9.203433_fragment_1	46275	Yes	Myoviridae	Vibrio	Vibrionaceae
NODE_5783_length_6437_cov_39.399561	6437	No	Microviridae	Parabacteroides	Tannerellaceae
NODE_17_length_250608_cov_14.422737_fragment_4	54952	Yes	NA	Parabacteroides	Tannerellaceae
NODE_844_length_45651_cov_324.299325	45651	No	Myoviridae	Vibrio	Vibrionaceae
NODE_124_length_121447_cov_14.821380_fragment_1	54970	Yes	Myoviridae	Vibrio	Vibrionaceae
NODE_36_length_202311_cov_15.065135_fragment_2_1	27999	Yes	NA	NA	NA
NODE_680_length_52792_cov_9.256689_1	45948	Yes	NA	NA	NA
NODE_599_length_57638_cov_12.325426_fragment_1	53422	Yes	NA	Faecalibacterium	Ruminococcaceae
NODE_475_length_65457_cov_12.325372_fragment_1	47700	Yes	NA	NA	NA
NODE_6827_length_5389_cov_11.722910	5389	No	Microviridae	NA	NA

NODE_791_length_31365_cov_10.204919	31365	No	Myoviridae	Serratia,Citrobacter,Burkholderia,Enterobacteria,Erwinia,Escherichia,Mannheimia,Pseudomonas,Ralstonia,Salmonella,Stenotrophomonas,Yersinia	Yersiniaceae,Enterobacteriaceae,Burkholderiaceae,Erwiniaceae,Pasteurellaceae,Pseudomonadaceae,Xanthomonadaceae
NODE_627_length_38672_cov_36.053992	38672	No	NA	NA	NA
NODE_594_length_40929_cov_21.387899	40929	No	NA	NA	NA
NODE_76_length_153211_cov_13.422798_fragment_1	51539	Yes	NA	Longibaculum	Erysipelotrichaceae
NODE_190_length_93830_cov_295.299206_fragment_1	80461	Yes	Siphoviridae	Riemerella	Weeksellaceae
NODE_36_length_193731_cov_67.333846_fragment_4	63984	Yes	NA	NA	NA
NODE_245_length_81023_cov_73.473026_fragment_1	55728	Yes	NA	NA	NA
NODE_584_length_41616_cov_11.227738	41616	No	Siphoviridae	Odoribacter,Bacteroides,Croceibacter	Marinifilaceae,Bacteroidaceae,Flavobacteriaceae
NODE_117_length_127184_cov_46.403016_fragment_2	62223	Yes	Myoviridae	Vibrio	Vibrionaceae
NODE_240_length_82040_cov_311.142014_fragment_1_1	62512	Yes	NA	NA	NA
NODE_58_length_138076_cov_27.537454_fragment_1	41983	Yes	NA	NA	NA
NODE_559_length_35643_cov_22.688800	35643	No	Siphoviridae	Microbacterium	Microbacteriaceae
NODE_46_length_149974_cov_10.328357_fragment_2_1	35758	Yes	Siphoviridae	Clostridium	Clostridiaceae
NODE_196_length_74574_cov_17.879225_fragment_1	32884	Yes	NA	NA	NA
NODE_352_length_51399_cov_10.225674_fragment_1	35599	Yes	Siphoviridae	Erysipelothrix	Erysipelotrichaceae
NODE_64_length_134578_cov_12.930956	134578	No	NA	NA	NA
NODE_110_length_106464_cov_23.011409_fragment_1	48166	Yes	NA	NA	NA
NODE_131_length_93476_cov_130.491752_fragment_1	61721	Yes	NA	Faecalibacterium	Ruminococcaceae
NODE_3887_length_5441_cov_866.652247	5441	No	Microviridae	Enterobacteria,Escherichia	Enterobacteriaceae
NODE_4_length_362764_cov_30.528327_fragment_7	108046	Yes	NA	NA	NA
NODE_504_length_39821_cov_68.576271	39821	No	NA	Bifidobacterium	Bifidobacteriaceae

NODE_450_length_42768_cov_14.032613	42768	No	NA	NA	NA
NODE_1019_length_36504_cov_17.010590_1	32981	Yes	NA	NA	NA
NODE_44_length_238795_cov_97.239482_fragment_2	76097	Yes	NA	NA	NA
NODE_726_length_46449_cov_141.263806	46449	No	NA	NA	NA
NODE_137_length_127923_cov_74.358018_fragment_2	61049	Yes	NA	NA	NA
NODE_175_length_108363_cov_15.794540_fragment_3	61958	Yes	NA	NA	NA
NODE_114_length_139538_cov_14.064223_fragment_1	36999	Yes	NA	NA	NA
NODE_91_length_155022_cov_15.206134_fragment_1_1	45754	Yes	NA	NA	NA
NODE_92_length_152852_cov_9.782168_fragment_2	43054	Yes	NA	NA	NA
NODE_488_length_60955_cov_41.624023_fragment_1_1	38856	Yes	NA	NA	NA
NODE_314_length_78602_cov_16.513489_fragment_1	52751	Yes	Myoviridae	Vibrio	Vibrionaceae
NODE_561_length_55332_cov_35.132315	55332	No	NA	NA	NA
NODE_6488_length_5863_cov_9.097624	5863	No	Microviridae	NA	NA
NODE_82_length_164445_cov_12.085595_fragment_3_1	33432	Yes	NA	NA	NA
NODE_1067_length_35225_cov_7.199090	35225	No	NA	NA	NA
NODE_252_length_89562_cov_9.708794_fragment_1	38800	Yes	Siphoviridae	Clostridium	Clostridiaceae
NODE_324_length_77953_cov_18.009987_fragment_1	38429	Yes	Siphoviridae	Clostridium	Clostridiaceae
NODE_165_length_130555_cov_35.203119_fragment_1	66824	Yes	NA	NA	NA
NODE_1147_length_36163_cov_8.300986_1	32841	Yes	Siphoviridae	Erysipelothrix	Erysipelotrichaceae
NODE_649_length_59546_cov_6.584206_fragment_1_1	35088	Yes	Siphoviridae	Erysipelothrix	Erysipelotrichaceae
NODE_33_length_236524_cov_42.598260_fragment_2_1	55637	Yes	Myoviridae	Faecalibacterium,Vibrio	Ruminococcaceae,Vibrionaceae
NODE_6_length_436617_cov_48.518123_fragment_3	42714	Yes	Myoviridae	Vibrio	Vibrionaceae
NODE_1106_length_37765_cov_164.656961	37765	No	NA	NA	NA
NODE_333_length_91772_cov_32.139211_fragment_1	39457	Yes	Siphoviridae	Clostridium	Clostridiaceae
NODE_927_length_44357_cov_9.030924	44357	No	NA	NA	NA

NODE_228_length_112530_cov_80.972812_fragment_1	62106	Yes	NA	NA	NA
NODE_5993_length_5441_cov_1513.124954	5441	No	Microviridae	Enterobacteria,Escherichia	Enterobacteriaceae
NODE_56_length_197880_cov_31.687830_fragment_2_1	38601	Yes	Siphoviridae	Clostridium	Clostridiaceae
NODE_217_length_115487_cov_12.163282_fragment_1	33470	Yes	NA	NA	NA
NODE_660_length_58995_cov_6.270224_fragment_1_1	41166	Yes	NA	NA	NA
NODE_281_length_100991_cov_19.589215_fragment_1	53158	Yes	NA	NA	NA
NODE_647_length_59803_cov_111.466777	59803	No	NA	NA	NA
NODE_77_length_179001_cov_18.978161_fragment_1_1	154124	Yes	NA	NA	NA
NODE_519_length_69308_cov_13.098566_1	55104	Yes	NA	NA	NA
NODE_961_length_43206_cov_278.383815	43206	No	NA	NA	NA
NODE_910_length_45560_cov_44.335392	45560	No	NA	NA	NA
NODE_405_length_58745_cov_70.779673_fragment_1	35746	Yes	Siphoviridae	Erysipelothrix	Erysipelotrichaceae
NODE_627_length_41501_cov_17.877165	41501	No	NA	NA	NA
NODE_438_length_55344_cov_286.490315	55344	No	NA	Faecalibacterium	Ruminococcaceae
NODE_575_length_45044_cov_24.374203	45044	No	NA	NA	NA
NODE_346_length_66391_cov_40.862835_fragment_1	58483	Yes	NA	NA	NA
NODE_684_length_38820_cov_19.506875	38820	No	Siphoviridae	Erysipelothrix	Erysipelotrichaceae
NODE_14_length_288476_cov_30.364381_fragment_1_1	153295	Yes	NA	NA	NA
NODE_456_length_53137_cov_62.110565_fragment_1	37811	Yes	Siphoviridae	Clostridium	Clostridiaceae
NODE_115_length_129856_cov_190.336076_fragment_4_1	62785	Yes	NA	NA	NA
NODE_303_length_73065_cov_73.952883_fragment_1	38132	Yes	NA	NA	NA
NODE_669_length_39764_cov_10.465587	39764	No	NA	NA	NA
NODE_272_length_78454_cov_64.153140	78454	No	NA	NA	NA
NODE_683_length_38823_cov_11.792870	38823	No	Siphoviridae	Clostridium	Clostridiaceae
NODE_34_length_214519_cov_198.166000_fragment_1	60105	Yes	Siphoviridae	Riemerella	Weeksellaceae

NODE_99_length_136438_cov_90.505540_fragment_1_1	34762	Yes	NA	Bifidobacterium	Bifidobacteriaceae
NODE_444_length_45364_cov_16.513761	45364	No	NA	NA	NA
NODE_173_length_76032_cov_10.899075	76032	No	NA	NA	NA
NODE_676_length_34274_cov_10.835442	34274	No	NA	Ruminococcus	Oscillospiraceae
NODE_1761_length_16233_cov_69.750798	16233	No	NA	NA	NA
NODE_237_length_65468_cov_14.139835	65468	No	NA	NA	NA
NODE_5253_length_6220_cov_79.792052	6220	No	Microviridae	Parabacteroides	Tannerellaceae
NODE_508_length_41407_cov_11.504038	41407	No	NA	NA	NA
NODE_98_length_97946_cov_32.458020	97946	No	Unassigned	uncultured~crAssphage	uncultured~crAssphage
NODE_5701_length_5811_cov_7.734538	5811	No	Microviridae	NA	NA
NODE_120_length_88900_cov_19.587259_fragment_2	40021	Yes	NA	NA	NA
NODE_53_length_120798_cov_48.820031_fragment_3	36195	Yes	NA	NA	NA
NODE_699_length_33255_cov_32.959036	33255	No	NA	NA	NA
NODE_285_length_58371_cov_11.397730	58371	No	NA	NA	NA
NODE_59_length_143882_cov_11.189498_fragment_2_1	65843	Yes	NA	NA	NA
NODE_111_length_106580_cov_33.628378_fragment_1	46787	Yes	NA	NA	NA
NODE_545_length_33197_cov_5.459538	33197	No	NA	NA	NA
NODE_6_length_296260_cov_82.330271_fragment_3	44972	Yes	Siphoviridae	Ruminococcus,Erysipelothrix	Oscillospiraceae,Erysipelotrichaceae
NODE_305_length_52841_cov_10.708445	52841	No	Myoviridae	Vibrio	Vibrionaceae
NODE_82_length_121471_cov_52.935527_fragment_1_1	48636	Yes	Myoviridae	Vibrio	Vibrionaceae
NODE_4003_length_5441_cov_782.383773	5441	No	Microviridae	Enterobacteria,Escherichia	Enterobacteriaceae
NODE_130_length_97829_cov_31.194264_fragment_1	50643	Yes	NA	NA	NA
NODE_3_length_372139_cov_63.847174_fragment_2_1	141926	Yes	NA	NA	NA
NODE_331_length_49956_cov_12.552334_fragment_1	38471	Yes	NA	NA	NA
NODE_363_length_46069_cov_8.116769	46069	No	NA	NA	NA

NODE_58_length_143896_cov_63.352765_fragment_1_1	69403	Yes	NA	NA	NA
NODE_48_length_158897_cov_51.026920_fragment_1_1	60277	Yes	Siphoviridae	Streptomyces	Streptomycetaceae
NODE_444_length_42194_cov_7.674553	42194	No	NA	NA	NA
NODE_4334_length_5818_cov_8.888947	5818	No	Microviridae	NA	NA
NODE_614_length_33416_cov_16.086508	33416	No	NA	NA	NA
NODE_4996_length_5058_cov_16.991005	5058	No	Microviridae	Bdellovibrio,Chlamydia	Bdellovibrionaceae,Chlamydiaceae
NODE_490_length_39244_cov_8.296410	39244	No	Siphoviridae	Clostridium	Clostridiaceae
NODE_202_length_70675_cov_27.287114_fragment_1	51252	Yes	Unassigned	Bacillus	Bacillaceae
NODE_5_length_274934_cov_23.636262_fragment_1	132559	Yes	NA	NA	NA
NODE_243_length_62886_cov_23.096736_1	59127	Yes	NA	NA	NA
NODE_429_length_43167_cov_76.533262	43167	No	NA	NA	NA
NODE_369_length_64042_cov_10.651101_fragment_1	47405	Yes	NA	NA	NA
NODE_187_length_97594_cov_29.379243	97594	No	Unassigned	uncultured~crAssphage	uncultured~crAssphage
NODE_51_length_177138_cov_40.677005_fragment_3_1	51239	Yes	NA	NA	NA
NODE_971_length_29273_cov_9.372202	29273	No	NA	NA	NA
NODE_280_length_76791_cov_106.203334_1	72756	Yes	NA	NA	NA
NODE_3438_length_5477_cov_24.367208	5477	No	Microviridae	NA	NA
NODE_444_length_57353_cov_25.062969_1	53008	Yes	NA	Parabacteroides	Tannerellaceae
NODE_861_length_32916_cov_12.249901	32916	No	NA	NA	NA
NODE_395_length_61505_cov_14.505110_fragment_1	42784	Yes	Siphoviridae	Clostridium	Clostridiaceae
NODE_193_length_96887_cov_103.136887_fragment_1	58190	Yes	NA	NA	NA
NODE_90_length_141357_cov_38.153536_fragment_1	31855	Yes	NA	NA	NA
NODE_99_length_137408_cov_62.230974_fragment_2	51929	Yes	NA	NA	NA
NODE_533_length_54935_cov_27.532106_1	39194	Yes	Myoviridae,Unassigned	Clostridium,Streptococcus,Thermus	Clostridiaceae,Streptococcaceae,Thermaceae

NODE_101_length_119284_cov_53.246651_fragment_1	38647	Yes	NA	NA	NA
NODE_1146_length_34302_cov_121.546763	34302	No	NA	NA	NA
NODE_33_length_185082_cov_26.372362_fragment_1	131770	Yes	NA	NA	NA
NODE_17_length_233795_cov_19.694905_fragment_1	40369	Yes	Siphoviridae	Erysipelothrix	Erysipelotrichaceae
NODE_820_length_42589_cov_7.631777	42589	No	NA	NA	NA
NODE_167_length_96019_cov_111.608020	96019	No	NA	NA	NA
NODE_324_length_70113_cov_50.813854_1	64962	Yes	NA	NA	NA
NODE_7842_length_5441_cov_846.019309	5441	No	Microviridae	Enterobacteria,Escherichia	Enterobacteriaceae
NODE_184_length_92019_cov_42.192586	92019	No	Unassigned	uncultured~crAssphage	uncultured~crAssphage
NODE_209_length_86114_cov_18.249875_fragment_1	52228	Yes	NA	NA	NA
NODE_194_length_88909_cov_8.119229_fragment_1_1	35100	Yes	Siphoviridae	Erysipelothrix	Erysipelotrichaceae
NODE_285_length_74430_cov_56.907200_fragment_1	36318	Yes	NA	NA	NA
NODE_202_length_77808_cov_6.557445_fragment_1	48603	Yes	NA	NA	NA
NODE_151_length_96812_cov_197.645896	96812	No	Unassigned	uncultured~crAssphage	uncultured~crAssphage
NODE_132_length_102332_cov_155.057491_fragment_1_1	60672	Yes	NA	NA	NA
NODE_22_length_229501_cov_36.161214_fragment_1	39030	Yes	NA	NA	NA
NODE_495_length_33995_cov_13.419623	33995	No	NA	NA	NA
NODE_13_length_269175_cov_35.606049_fragment_3_1	43012	Yes	NA	NA	NA
NODE_434_length_39185_cov_10.139356	39185	No	NA	NA	NA
NODE_17_length_244300_cov_101.781727_fragment_1	35110	Yes	NA	NA	NA
NODE_69_length_108351_cov_7.887844_fragment_1	52818	Yes	NA	NA	NA
NODE_53_length_119863_cov_23.660832_fragment_2_1	58474	Yes	Siphoviridae	Streptomyces	Streptomycetaceae
NODE_157_length_73125_cov_22.604489_fragment_1_1	47526	Yes	Myoviridae	Vibrio	Vibrionaceae
NODE_18_length_191858_cov_24.519908_fragment_2	35849	Yes	Siphoviridae	Paenibacillus	Paenibacillaceae
NODE_235_length_59720_cov_10.955770	59720	No	NA	NA	NA

NODE_251_length_57312_cov_6.957210_1	53064	Yes	NA	Parabacteroides	Tannerellaceae
NODE_17_length_193818_cov_187.865057_fragment_2	76029	Yes	NA	NA	NA
NODE_751_length_34660_cov_8.277388	34660	No	NA	NA	NA
NODE_720_length_36339_cov_9.224865	36339	No	NA	NA	NA
NODE_314_length_66981_cov_14.405493	66981	No	NA	NA	NA
NODE_114_length_125972_cov_8.815243_fragment_3	33508	Yes	Siphoviridae	Clostridium	Clostridiaceae
NODE_333_length_63434_cov_8.272646_1	58021	Yes	Siphoviridae	Streptomyces	Streptomycetaceae
NODE_48_length_186349_cov_10.450100_fragment_1_1	35997	Yes	NA	NA	NA
NODE_568_length_44362_cov_7.137653	44362	No	Myoviridae	Vibrio	Vibrionaceae
NODE_8_length_420352_cov_31.496803_fragment_4	37214	Yes	NA	NA	NA
NODE_641_length_40826_cov_6.918324	40826	No	NA	Akkermansia	Akkermansiaceae
NODE_356_length_60343_cov_50.281698	60343	No	Myoviridae	Vibrio	Vibrionaceae
NODE_193_length_95353_cov_15.542236	95353	No	Unassigned	uncultured~crAssphage	uncultured~crAssphage
NODE_169_length_100773_cov_14.257730	100773	No	Unassigned	Cellulophaga	Flavobacteriaceae
NODE_460_length_42766_cov_7.851280	42766	No	NA	NA	NA
NODE_85_length_122985_cov_8.468966_fragment_1_1	42636	Yes	NA	NA	NA
NODE_4_length_492147_cov_58.946526_fragment_3_1	37485	Yes	NA	NA	NA
NODE_162_length_86356_cov_13.572867_fragment_1	34161	Yes	NA	NA	NA
NODE_502_length_38910_cov_59.741989_1	30835	Yes	NA	Faecalibacterium	Ruminococcaceae
NODE_56_length_146955_cov_16.925228_fragment_2	41509	Yes	NA	Bifidobacterium	Bifidobacteriaceae
NODE_400_length_47089_cov_10.202662	47089	No	Myoviridae	Vibrio	Vibrionaceae
NODE_7_length_344950_cov_50.876870_fragment_1_1	59372	Yes	Siphoviridae	Streptomyces	Streptomycetaceae
NODE_5823_length_5217_cov_4.980628	5217	No	Microviridae	NA	NA
NODE_7_length_545448_cov_98.446660_fragment_2	40204	Yes	NA	NA	NA
NODE_609_length_38632_cov_10.843534	38632	No	Siphoviridae	Veillonella,Bacillus	Veillonellaceae,Bacillaceae

NODE_52_length_244973_cov_200.701741_fragment_3	46280	Yes	NA	Bacteroides	Bacteroidaceae
NODE_8_length_472479_cov_87.017565_fragment_7	47853	Yes	Myoviridae	Vibrio	Vibrionaceae
NODE_73_length_204507_cov_32.715234_fragment_1_1	34037	Yes	NA	NA	NA
NODE_370_length_60991_cov_19.570943_fragment_1	35474	Yes	NA	NA	NA
NODE_35_length_290450_cov_25.004618_fragment_1	65342	Yes	NA	NA	NA
NODE_554_length_59605_cov_69.070193	59605	No	NA	NA	NA
NODE_94_length_150498_cov_33.756027_fragment_2	41229	Yes	Siphoviridae	Bacillus	Bacillaceae
NODE_494_length_64282_cov_35.415044_fragment_1_1	29855	Yes	NA	NA	NA
NODE_6_length_374713_cov_22.072023_fragment_2	40300	Yes	Siphoviridae	Clostridium	Clostridiaceae
NODE_18_length_310600_cov_23.407355_fragment_3	54666	Yes	NA	Parabacteroides	Tannerellaceae
NODE_39_length_203789_cov_29.999975_fragment_3	39685	Yes	Siphoviridae	Erysipelothrix	Erysipelotrichaceae
NODE_843_length_43741_cov_28.426590_1	35300	Yes	NA	Bifidobacterium	Bifidobacteriaceae
NODE_984_length_39159_cov_197.692538	39159	No	NA	NA	NA
NODE_761_length_47879_cov_15.525155	47879	No	NA	NA	NA
NODE_78_length_161341_cov_193.105756_fragment_1	126100	Yes	NA	NA	NA
NODE_20_length_305595_cov_58.532621_fragment_3	41632	Yes	NA	Bifidobacterium	Bifidobacteriaceae
NODE_5_length_411242_cov_16.851306_fragment_3_1	42018	Yes	Siphoviridae	Erysipelothrix	Erysipelotrichaceae
NODE_582_length_57863_cov_8.992959	57863	No	NA	Roseburia	Lachnospiraceae
NODE_1114_length_35177_cov_5.919595	35177	No	NA	NA	NA
NODE_574_length_58234_cov_22.757369	58234	No	NA	NA	NA
NODE_396_length_73730_cov_13.211021_fragment_1_1	56376	Yes	Siphoviridae	Streptomyces	Streptomycetaceae
NODE_3_length_423041_cov_20.715976_fragment_1_1	63403	Yes	NA	Parabacteroides	Tannerellaceae
NODE_444_length_68664_cov_230.856098_fragment_1_1	38682	Yes	Siphoviridae	Clostridium	Clostridiaceae
NODE_200_length_105355_cov_71.470665_fragment_1	53921	Yes	NA	Bifidobacterium	Bifidobacteriaceae
NODE_432_length_69693_cov_33.370674	69693	No	NA	NA	NA

NODE_596_length_57178_cov_195.202318	57178	No	NA	NA	NA
NODE_678_length_52441_cov_22.492345	52441	No	NA	NA	NA
NODE_3363_length_12409_cov_292.185284	12409	No	Siphoviridae	Paenibacillus	Paenibacillaceae
NODE_856_length_38879_cov_12.096512	38879	No	NA	NA	NA
NODE_33_length_223128_cov_109.284499_fragment_3_1	67006	Yes	NA	NA	NA
NODE_517_length_58399_cov_12.518477	58399	No	Siphoviridae	Streptomyces	Streptomycetaceae
NODE_153_length_120481_cov_29.311735	120481	No	NA	NA	NA
NODE_9_length_317500_cov_43.346170_fragment_1_1	60553	Yes	NA	NA	NA
NODE_823_length_40413_cov_14.193146	40413	No	NA	NA	NA
NODE_209_length_102756_cov_15.336832_fragment_1	40003	Yes	NA	Acidaminococcus	Acidaminococcaceae
NODE_566_length_54469_cov_10.704947	54469	No	NA	NA	NA
NODE_690_length_46374_cov_51.971675_fragment_1	33898	Yes	NA	NA	NA
NODE_262_length_93040_cov_33.101339_fragment_1	70895	Yes	NA	NA	NA
NODE_682_length_35893_cov_9.698532	35893	No	NA	NA	NA
NODE_384_length_53740_cov_11.247648_1	50342	Yes	Siphoviridae	Longibaculum,Clostridium,Streptococcus	Erysipelotrichaceae,Clostridiaceae,Streptococcaceae
NODE_35_length_198405_cov_31.971485_fragment_1	59209	Yes	NA	Faecalibacterium	Ruminococcaceae
NODE_556_length_41750_cov_103.956302	41750	No	NA	NA	NA
NODE_329_length_59459_cov_9.632011_fragment_1	34702	Yes	NA	NA	NA
NODE_139_length_99402_cov_6.527172	99402	No	Unassigned	Cellulophaga	Flavobacteriaceae
NODE_227_length_74550_cov_8.146533_1	65403	Yes	NA	NA	NA
NODE_312_length_42733_cov_7.076316_fragment_1	37144	Yes	NA	NA	NA
NODE_15_length_191903_cov_49.822782_fragment_2_1	55111	Yes	NA	NA	NA
NODE_278_length_46208_cov_5.966459	46208	No	NA	NA	NA
NODE_12_length_217731_cov_551.127001_fragment_1	52361	Yes	Siphoviridae	Riemerella	Weeksellaceae

NODE_52_length_121670_cov_5.951577_fragment_1	33352	Yes	NA	NA	NA
NODE_97_length_129925_cov_18.453122_fragment_1	47852	Yes	NA	NA	NA
NODE_264_length_78618_cov_59.717526_fragment_2	46806	Yes	NA	NA	NA
NODE_361_length_63936_cov_6.708458	63936	No	NA	NA	NA
NODE_764_length_34945_cov_15.628833	34945	No	NA	NA	NA
NODE_163_length_106149_cov_28.447301_fragment_1	70412	Yes	NA	NA	NA
NODE_499_length_50776_cov_16.208257	50776	No	Myoviridae	Faecalibacterium,Vibrio	Ruminococcaceae,Vibrionaceae
NODE_261_length_78902_cov_11.721600	78902	No	NA	NA	NA
NODE_36_length_180841_cov_136.548737_fragment_1	53301	Yes	NA	Parabacteroides	Tannerellaceae
NODE_383_length_62362_cov_29.926702_fragment_1_1	35918	Yes	Siphoviridae	Clostridium	Clostridiaceae
NODE_495_length_66151_cov_9.764721	66151	No	NA	NA	NA
NODE_335_length_82289_cov_143.642167_fragment_1	60624	Yes	NA	NA	NA
NODE_620_length_56242_cov_149.157795	56242	No	NA	Faecalibacterium	Ruminococcaceae
NODE_1043_length_38387_cov_15.348664	38387	No	NA	NA	NA
NODE_7071_length_5611_cov_36.957703	5611	No	Microviridae	NA	NA
NODE_205_length_104244_cov_8.084903_fragment_1	40334	Yes	NA	NA	NA
NODE_115_length_135744_cov_79.815932_fragment_1	53487	Yes	Myoviridae	Vibrio	Vibrionaceae
NODE_398_length_74414_cov_12.354846_fragment_1	30335	Yes	NA	NA	NA
NODE_637_length_55169_cov_16.861070_fragment_1	38727	Yes	NA	NA	NA
NODE_179_length_110675_cov_39.459004_fragment_2_1	63107	Yes	NA	NA	NA
NODE_878_length_43412_cov_20.286113	43412	No	NA	NA	NA
NODE_200_length_105288_cov_67.465871_fragment_1_1	56779	Yes	Siphoviridae	Streptomyces	Streptomycetaceae
NODE_48_length_193268_cov_16.916470_fragment_3	38847	Yes	NA	NA	NA
NODE_632_length_55490_cov_147.411040	55490	No	NA	NA	NA
NODE_723_length_50108_cov_10.526042	50108	No	Myoviridae	Roseburia,Vibrio	Lachnospiraceae,Vibrionaceae

NODE_574_length_59570_cov_19.600134_fragment_1	40379	Yes	NA	NA	NA
NODE_49_length_185379_cov_9.012524	185379	No	Unassigned	uncultured~crAssphage	uncultured~crAssphage
NODE_301_length_86202_cov_159.335520	86202	No	NA	NA	NA
NODE_122_length_88304_cov_21.343460_fragment_1_1	59749	Yes	NA	NA	NA
NODE_435_length_40943_cov_9.563319	40943	No	NA	NA	NA
NODE_363_length_46425_cov_13.290554	46425	No	Myoviridae	Vibrio	Vibrionaceae
NODE_34_length_156135_cov_9.999731_fragment_1	42261	Yes	NA	NA	NA
NODE_280_length_64177_cov_21.606375	64177	No	NA	NA	NA
NODE_239_length_71027_cov_7.905794_1	62760	Yes	NA	NA	NA
NODE_195_length_81272_cov_7.699361_fragment_1	50606	Yes	Myoviridae	Vibrio	Vibrionaceae
NODE_126_length_99127_cov_13.835837	99127	No	Unassigned	uncultured~crAssphage	uncultured~crAssphage
NODE_45_length_145463_cov_13.867160_fragment_1_1	46602	Yes	Myoviridae	Vibrio	Vibrionaceae
NODE_103_length_107693_cov_8.203209_fragment_2	64744	Yes	Siphoviridae	Streptomyces	Streptomycetaceae
NODE_327_length_58487_cov_9.169548	58487	No	NA	NA	NA
NODE_535_length_41652_cov_21.860158_1	33081	Yes	NA	NA	NA
NODE_36_length_160068_cov_13.798360_fragment_1	50238	Yes	NA	NA	NA
NODE_523_length_42560_cov_9.954100	42560	No	NA	NA	NA
NODE_35_length_162363_cov_19.641022_fragment_1	42399	Yes	NA	NA	NA
NODE_272_length_65995_cov_7.787701_fragment_1_1	57975	Yes	NA	NA	NA
NODE_66_length_125749_cov_10.742000_fragment_1	109729	Yes	NA	NA	NA
NODE_146_length_92349_cov_15.414837_fragment_2	47398	Yes	Myoviridae	Eubacterium,Vibrio	Eubacteriaceae,Vibrionaceae
NODE_504_length_37331_cov_12.205494	37331	No	Siphoviridae	Clostridium	Clostridiaceae
NODE_448_length_40199_cov_132.519804	40199	No	NA	NA	NA
NODE_297_length_56438_cov_8.123530_fragment_1	43389	Yes	NA	NA	NA
NODE_67_length_133469_cov_21.072706	133469	No	NA	NA	NA

NODE_325_length_52615_cov_6.364441_fragment_1	41746	Yes	Myoviridae	Vibrio	Vibrionaceae
NODE_54_length_145031_cov_11.915027_fragment_2	37501	Yes	Siphoviridae	Erysipelothrix	Erysipelotrichaceae
NODE_366_length_47119_cov_13.281680	47119	No	NA	NA	NA
NODE_14_length_207116_cov_76.725574_fragment_1_1	159829	Yes	NA	Roseburia	Lachnospiraceae
NODE_55_length_144871_cov_89.029348_fragment_2_1	85842	Yes	NA	NA	NA
NODE_4373_length_5070_cov_65.294915	5070	No	Microviridae	Phascolarctobacterium,Bdellovibrio,Chlamydia	Acidaminococcaceae,Bdellovibrionaceae,Chlamydiaceae
NODE_17_length_194657_cov_19.834236_fragment_1_1	66853	Yes	NA	NA	NA
NODE_376_length_46613_cov_7.230422	46613	No	Myoviridae	Vibrio	Vibrionaceae
NODE_311_length_54219_cov_29.350750	54219	No	Myoviridae	Vibrio	Vibrionaceae
NODE_560_length_36629_cov_12.871767	36629	No	NA	NA	NA
NODE_465_length_44099_cov_9.663632_1	35456	Yes	NA	Veillonella	Veillonellaceae
NODE_485_length_41698_cov_9.796965	41698	No	NA	NA	NA
NODE_292_length_65851_cov_9.764530_fragment_1_1	38353	Yes	NA	NA	NA
NODE_1_length_692657_cov_53.348528_fragment_1	151367	Yes	NA	NA	NA
NODE_304_length_37256_cov_7.332867	37256	No	Siphoviridae	Arthrobacter	Micrococcaceae
NODE_36_length_153980_cov_97.741303_fragment_2	35075	Yes	NA	NA	NA
NODE_160_length_59728_cov_9.952843	59728	No	NA	NA	NA
NODE_294_length_66591_cov_22.943685	66591	No	NA	NA	NA
NODE_534_length_44240_cov_14.672649_fragment_1	38445	Yes	NA	NA	NA
NODE_912_length_28569_cov_19.425125	28569	No	NA	Bifidobacterium	Bifidobacteriaceae
NODE_239_length_76500_cov_8.998378_fragment_1_1	44621	Yes	NA	NA	NA
NODE_531_length_44297_cov_89.864699	44297	No	NA	NA	NA
NODE_647_length_37924_cov_7.975679	37924	No	NA	NA	NA
NODE_368_length_55913_cov_42.146031	55913	No	NA	Faecalibacterium	Ruminococcaceae

NODE_67_length_149733_cov_12.029343_fragment_2_1	40738	Yes	NA	NA	NA
NODE_168_length_92964_cov_8.329376	92964	No	Unassigned	uncultured~crAssphage	uncultured~crAssphage
NODE_308_length_64747_cov_37.868268_fragment_1	46483	Yes	Myoviridae	Vibrio	Vibrionaceae
NODE_104_length_123587_cov_36.455939_fragment_1	95106	Yes	NA	NA	NA
NODE_670_length_36566_cov_9.281970	36566	No	Unassigned	Bacillus	Bacillaceae
NODE_593_length_40787_cov_44.597417	40787	No	NA	NA	NA
NODE_456_length_48662_cov_29.504948_1	43154	Yes	NA	NA	NA
NODE_91_length_128770_cov_89.184967_fragment_2_1	58473	Yes	NA	NA	NA
NODE_6293_length_5441_cov_862.167843	5441	No	Microviridae	Enterobacteria,Escherichia	Enterobacteriaceae
NODE_1_length_637477_cov_23.037423_fragment_2_1	57485	Yes	NA	NA	NA
NODE_374_length_55296_cov_6.386199_fragment_1	44503	Yes	NA	NA	NA
NODE_399_length_53027_cov_6.608888	53027	No	Myoviridae	Vibrio	Vibrionaceae
NODE_57_length_159620_cov_36.311353_fragment_1_1	62748	Yes	NA	Eubacterium	Eubacteriaceae
NODE_139_length_106014_cov_9.634236_fragment_2	50023	Yes	NA	NA	NA
NODE_7_length_281358_cov_18.651099_fragment_5_1	58498	Yes	NA	NA	NA
NODE_313_length_56073_cov_40.008265	56073	No	NA	Faecalibacterium	Ruminococcaceae
NODE_136_length_100464_cov_31.325190	100464	No	Unassigned	Cellulophaga	Flavobacteriaceae
NODE_455_length_43432_cov_19.633769	43432	No	NA	Faecalibacterium,Acidipropionibacterium	Ruminococcaceae,Propionibacteriaceae
NODE_29_length_198871_cov_16.002978_fragment_1_1	140533	Yes	NA	NA	NA
NODE_276_length_60494_cov_16.563444	60494	No	NA	Faecalibacterium	Ruminococcaceae
NODE_307_length_56420_cov_51.180662	56420	No	NA	Faecalibacterium	Ruminococcaceae
NODE_2274_length_10639_cov_12.374339	10639	No	NA	NA	NA
NODE_59_length_141838_cov_14.836927_fragment_2	45508	Yes	NA	NA	NA
NODE_373_length_49595_cov_10.054219	49595	No	NA	NA	NA
NODE_453_length_45847_cov_10.183766_fragment_1	35375	Yes	NA	NA	NA

NODE_77_length_125857_cov_11.999610_fragment_1	83698	Yes	NA	NA	NA
NODE_74_length_126823_cov_244.795966_fragment_1	70186	Yes	NA	NA	NA
NODE_3671_length_6644_cov_15.182122	6644	No	Microviridae	Parabacteroides	Tannerellaceae
NODE_356_length_52808_cov_6.537676_fragment_1	40105	Yes	NA	NA	NA
NODE_211_length_72075_cov_326.995598_fragment_1	41669	Yes	NA	NA	NA
NODE_334_length_54464_cov_19.115238	54464	No	Myoviridae	Vibrio	Vibrionaceae
NODE_629_length_53273_cov_20.167819_1	48705	Yes	NA	NA	NA
NODE_348_length_78483_cov_10.864755_fragment_1	57371	Yes	NA	NA	NA
NODE_882_length_39858_cov_11.643821	39858	No	Unassigned	Bacillus	Bacillaceae
NODE_341_length_79809_cov_14.453958_fragment_1	34615	Yes	NA	NA	NA
NODE_596_length_55629_cov_12.280761	55629	No	Myoviridae	Faecalibacterium,Vibrio	Ruminococcaceae,Vibrionaceae
NODE_418_length_71107_cov_10.915977_fragment_1	44563	Yes	NA	NA	NA
NODE_5963_length_5046_cov_18.341014	5046	No	Microviridae	Bdellovibrio,Chlamydia	Bdellovibrionaceae,Chlamydiaceae
NODE_28_length_233050_cov_11.857881_fragment_2_1	47068	Yes	NA	NA	NA
NODE_272_length_74167_cov_18.491796	74167	No	NA	NA	NA
NODE_454_length_51575_cov_26.673913	51575	No	Siphoviridae	Bacteroides,Croceibacter	Bacteroidaceae,Flavobacteriaceae
NODE_247_length_79853_cov_41.365899	79853	No	NA	NA	NA
NODE_198_length_91835_cov_19.770691	91835	No	NA	NA	NA
NODE_191_length_94170_cov_20.162216_fragment_1	66181	Yes	Siphoviridae	Riemerella	Weeksellaceae
NODE_60_length_180405_cov_21.397643_fragment_4_1	35594	Yes	NA	NA	NA
NODE_750_length_36236_cov_10.990133	36236	No	NA	NA	NA
NODE_6409_length_5091_cov_37.843725	5091	No	Microviridae	Bdellovibrio,Chlamydia	Bdellovibrionaceae,Chlamydiaceae
NODE_46_length_201566_cov_9.990343_fragment_2_1	53389	Yes	NA	NA	NA
NODE_547_length_45542_cov_16.847583	45542	No	NA	NA	NA

NODE_565_length_44183_cov_8.335841	44183	No	Siphoviridae	Odoribacter,Bacteroides,Croceibacter	Marinifilaceae,Bacteroidaceae,Flavobacteriaceae
NODE_186_length_95604_cov_37.782823	95604	No	NA	NA	NA
NODE_105_length_129331_cov_14.545747_fragment_1	109506	Yes	NA	NA	NA
NODE_387_length_54876_cov_17.838365_1	45262	Yes	NA	NA	NA
NODE_187_length_84151_cov_110.781963_fragment_1	49124	Yes	Myoviridae	Vibrio	Vibrionaceae
NODE_231_length_73951_cov_32.896503_fragment_2_1	33514	Yes	NA	NA	NA
NODE_250_length_71046_cov_14.847319_fragment_1	41840	Yes	Siphoviridae	Clostridium	Clostridiaceae
NODE_347_length_57840_cov_6.241412	57840	No	Siphoviridae	Riemerella	Weeksellaceae
NODE_399_length_54168_cov_32.431246	54168	No	Myoviridae	Vibrio	Vibrionaceae
NODE_721_length_34268_cov_15.353170	34268	No	Siphoviridae	Erysipelothrix	Erysipelotrichaceae
NODE_57_length_148513_cov_8.933436_fragment_1_1	57753	Yes	Siphoviridae	Streptomyces	Streptomycetaceae
NODE_302_length_63485_cov_13.160807	63485	No	NA	NA	NA
NODE_723_length_34245_cov_10.529044	34245	No	Siphoviridae	Lactococcus	Streptococcaceae
NODE_617_length_38323_cov_883.285251	38323	No	NA	NA	NA
NODE_222_length_92646_cov_48.389746_fragment_2	36053	Yes	NA	Eubacterium	Eubacteriaceae
NODE_97_length_136774_cov_12.770924_fragment_2_1	65599	Yes	NA	NA	NA
NODE_216_length_94225_cov_8.855453_fragment_2	39605	Yes	NA	NA	NA
NODE_122_length_120503_cov_55.953988_fragment_1_1	39134	Yes	NA	NA	NA
NODE_545_length_51506_cov_9.026705	51506	No	NA	NA	NA
NODE_93_length_139069_cov_18.810429_fragment_2	106086	Yes	NA	NA	NA
NODE_26_length_254232_cov_16.341128	254232	No	NA	NA	NA
NODE_3_length_495834_cov_23.184082_fragment_1	242864	Yes	NA	NA	NA
NODE_175_length_102585_cov_10.363893	102585	No	NA	NA	NA
NODE_17_length_299026_cov_9.534390_fragment_1_1	41004	Yes	NA	NA	NA

NODE_252_length_85848_cov_14.697959_fragment_1_1	39810	Yes	NA	NA	NA

Supplementary Table 3. Phage genome annotation information in oral phage ensemble

Phage ID	Genome length	Provirus	Taxonomy	Host bacteria (genus)	Host bacteria (family)
NODE_285_length_51925_cov_20.707634_fragment_1_1	40171	Yes	NA	NA	NA
NODE_125_length_85116_cov_22.698546_fragment_1	51309	Yes	Myoviridae	Fusobacterium,Vibrio	Fusobacteriaceae,Vibrionaceae
NODE_339_length_46752_cov_20.722798	46752	No	Podoviridae	Aquamicrobium,Burkholderia,Caulobacter,Escherichia,Pseudomonas,Ralstonia,Xanthomonas,Xylella	Phyllobacteriaceae,Burkholderiaceae,Caulobacteraceae,Enterobacteriaceae,Pseudomonadaceae,Xanthomonadaceae
NODE_5131_length_5441_cov_728.279057	5441	No	Microviridae	Enterobacteria,Escherichia	Enterobacteriaceae
NODE_2_length_690353_cov_38.535267_fragment_6	45330	Yes	Siphoviridae	Riemerella	Weeksellaceae
NODE_318_length_48373_cov_25.118300_fragment_1	34085	Yes	Myoviridae	Neisseria,Burkholderia,Enterobacteria,Erwinia,Escherichia,Mannheimia,Pseudomonas,Ralstonia,Salmonella,Stenotrophomonas,Yersinia	Neisseriaceae,Burkholderiaceae,Enterobacteriaceae,Erwiniaceae,Pasteurellaceae,Pseudomonadaceae,Xanthomonadaceae,Yersiniaceae
NODE_36_length_173795_cov_29.134828_fragment_2	39592	Yes	NA	NA	NA
NODE_370_length_43809_cov_183.284568	43809	No	NA	NA	NA
NODE_121_length_48237_cov_64.960836	48237	No	Myoviridae	Vibrio	Vibrionaceae
NODE_7_length_232865_cov_8.176848_fragment_3	59378	Yes	Podoviridae	Ottowia,Pseudomonas	Comamonadaceae,Pseudomonadaceae
NODE_2935_length_5441_cov_334.768288	5441	No	Microviridae	Enterobacteria,Escherichia	Enterobacteriaceae
NODE_197_length_35154_cov_5.638052	35154	No	NA	NA	NA
NODE_158_length_39335_cov_12.267133	39335	No	NA	NA	NA
NODE_233_length_36131_cov_10.691041	36131	No	NA	NA	NA

NODE_156_length_47628_cov_16.942131	47628	No	Unassigned,Siphoviridae,Myoviridae	Geobacillus,Lactobacillus,Listeria	Bacillaceae,Lactobacillaceae,Listeriaceae
NODE_6_length_347594_cov_14.422994_fragment_2	36652	Yes	NA	NA	NA
NODE_180_length_44485_cov_7.389939_1	36325	Yes	NA	NA	NA
NODE_2_length_641090_cov_148.288608_fragment_11	41248	Yes	NA	Corynebacterium	Corynebacteriaceae
NODE_77_length_85878_cov_11.914079_fragment_2	37609	Yes	NA	Leptotrichia	Leptotrichiaceae
NODE_3_length_273354_cov_25.075983_fragment_3_1	36270	Yes	NA	Streptococcus	Streptococcaceae
NODE_324_length_36514_cov_11.748155	36514	No	NA	NA	NA
NODE_392_length_31730_cov_14.505667	31730	No	NA	NA	NA
NODE_308_length_38463_cov_12.100890	38463	No	Myoviridae	Neisseria,Haemophilus,Mannheimia,Vibrio	Neisseriaceae,Pasteurellaceae,Vibrionaceae
NODE_291_length_39984_cov_9.487540_1	29239	Yes	NA	NA	NA
NODE_126_length_34395_cov_26.120093	34395	No	Myoviridae	Haemophilus,Mannheimia,Vibrio	Pasteurellaceae,Vibrionaceae
NODE_112_length_36474_cov_7.834839	36474	No	Siphoviridae	Streptococcus	Streptococcaceae
NODE_45_length_62254_cov_8.581119	62254	No	NA	NA	NA
NODE_15_length_121961_cov_10.608018	121961	No	NA	NA	NA
NODE_106_length_37327_cov_24.921281	37327	No	NA	Haemophilus	Pasteurellaceae
NODE_144_length_66824_cov_26.501969_fragment_1	38386	Yes	Siphoviridae	Cellulophaga	Flavobacteriaceae
NODE_89_length_91521_cov_103.954311_fragment_1	44155	Yes	NA	NA	NA
NODE_213_length_51420_cov_17.751290	51420	No	Siphoviridae	Riemerella	Weeksellaceae
NODE_3659_length_5441_cov_495.210917	5441	No	Microviridae	Enterobacteria,Escherichia	Enterobacteriaceae
NODE_41_length_138732_cov_15.648622_fragment_4_1	38838	Yes	NA	NA	NA
NODE_87_length_93593_cov_6.941553_fragment_1	40083	Yes	Myoviridae	Vibrio	Vibrionaceae
NODE_232_length_40352_cov_134.567685	40352	No	Siphoviridae	Corynebacterium	Corynebacteriaceae

NODE_845_length_17222_cov_49.703326	17222	No	Podoviridae	Actinomyces	Actinomycetaceae
NODE_147_length_57366_cov_20.689013	57366	No	Siphoviridae	Clavibacter,Microbacterium	Microbacteriaceae
NODE_4486_length_5441_cov_1511.654846	5441	No	Microviridae	Enterobacteria,Escherichia	Enterobacteriaceae
NODE_233_length_40222_cov_26.770807	40222	No	NA	NA	NA
NODE_199_length_44352_cov_46.652572_1	41203	Yes	NA	NA	NA
NODE_73_length_98700_cov_15.724629_fragment_1	38297	Yes	NA	Veillonella	Veillonellaceae
NODE_299_length_34667_cov_8.772911	34667	No	NA	NA	NA
NODE_342_length_40004_cov_60.867907	40004	No	NA	NA	NA
NODE_373_length_38207_cov_16.921341	38207	No	NA	NA	NA
NODE_327_length_41077_cov_89.287821	41077	No	NA	Corynebacterium	Corynebacteriaceae
NODE_88_length_86512_cov_15.253490_fragment_1_1	39390	Yes	NA	Fusobacterium	Fusobacteriaceae
NODE_241_length_49089_cov_14.758249	49089	No	NA	NA	NA
NODE_309_length_42398_cov_10.367971	42398	No	NA	Leptotrichia	Leptotrichiaceae
NODE_91_length_86266_cov_80.877382_fragment_1	36426	Yes	NA	Neisseria	Neisseriaceae
NODE_181_length_56676_cov_79.970523	56676	No	Siphoviridae	Arthrobacter	Micrococcaceae
NODE_10_length_251168_cov_60.662057_fragment_1_1	38028	Yes	NA	Neisseria	Neisseriaceae
NODE_84_length_87828_cov_10.880476_fragment_2	35955	Yes	Myoviridae	Burkholderia,Enterobacteria,Erwinia,Escherichia,Mannheimia,Pseudomonas,Ralstonia,Salmonella,Stenotrophomonas,Yersinia	Burkholderiaceae,Enterobacteriaceae,Erwiniaceae,Pasteurellaceae,Pseudomonadaceae,Xanthomonadaceae,Yersiniaceae
NODE_4652_length_5441_cov_31.201263	5441	No	Microviridae	Enterobacteria,Escherichia	Enterobacteriaceae
NODE_159_length_41277_cov_25.601936	41277	No	NA	NA	NA
NODE_36_length_135185_cov_7.136446_fragment_2_1	35824	Yes	NA	NA	NA
NODE_135_length_46825_cov_6.684071_fragment_1	36383	Yes	Siphoviridae	Streptococcus	Streptococcaceae
NODE_2601_length_5441_cov_17.492388	5441	No	Microviridae	Enterobacteria,Escherichia	Enterobacteriaceae

NODE_136_length_46575_cov_9.624291_1	40479	Yes	NA	NA	NA
NODE_35_length_139437_cov_10.586719_fragment_1_1	32378	Yes	NA	NA	NA
NODE_206_length_53121_cov_9.915125	53121	No	NA	NA	NA
NODE_192_length_55135_cov_7.417611	55135	No	NA	NA	NA
NODE_50_length_117078_cov_6.142784_fragment_1	37632	Yes	Siphoviridae	Pseudopropionibacterium,Arthrobacter	Propionibacteriaceae,Micrococcaceae
NODE_444_length_31341_cov_139.137921	31341	No	NA	Pseudopropionibacterium	Propionibacteriaceae
NODE_106_length_77553_cov_25.259091_fragment_1	36389	Yes	NA	NA	NA
NODE_174_length_57263_cov_37.005943_fragment_1	46876	Yes	NA	Leptotrichia	Leptotrichiaceae
NODE_220_length_51025_cov_11.295586	51025	No	NA	NA	NA
NODE_404_length_33901_cov_16.692637	33901	No	NA	Streptococcus	Streptococcaceae
NODE_333_length_39355_cov_4.923181	39355	No	NA	NA	NA
NODE_373_length_35769_cov_8.412163	35769	No	Siphoviridae	Streptococcus	Streptococcaceae
NODE_109_length_76216_cov_10.569675_fragment_1	41405	Yes	NA	Leptotrichia	Leptotrichiaceae
NODE_332_length_39374_cov_14.429741	39374	No	Myoviridae	Neisseria,Haemophilus,Mannheimia,Vibrio	Neisseriaceae,Pasteurellaceae,Vibrionaceae
NODE_4_length_445210_cov_39.864771_fragment_1	41863	Yes	Siphoviridae	Corynebacterium	Corynebacteriaceae
NODE_3138_length_5441_cov_542.283141	5441	No	Microviridae	Enterobacteria,Escherichia	Enterobacteriaceae
NODE_20_length_202067_cov_75.657149_fragment_1	42155	Yes	NA	Neisseria	Neisseriaceae
NODE_265_length_34208_cov_24.735924	34208	No	NA	NA	NA
NODE_113_length_68047_cov_27.130427	68047	No	NA	NA	NA
NODE_203_length_43033_cov_8.804063_fragment_1	38446	Yes	NA	Pseudopropionibacterium	Propionibacteriaceae
NODE_180_length_56686_cov_14.444121	56686	No	NA	NA	NA
NODE_47_length_95562_cov_48.142586_1	76815	Yes	NA	NA	NA
NODE_98_length_73595_cov_15.506949_fragment_1_1	32282	Yes	NA	NA	NA

NODE_327_length_40678_cov_9.074834	40678	No	NA	NA	NA
NODE_121_length_68508_cov_9.505529_fragment_1	46688	Yes	NA	NA	NA
NODE_346_length_39347_cov_18.238420_1	35982	Yes	NA	NA	NA
NODE_260_length_45677_cov_8.600237_1	39051	Yes	NA	NA	NA
NODE_5_length_209385_cov_21.420862_fragment_3	39665	Yes	NA	Streptococcus	Streptococcaceae
NODE_4_length_333726_cov_31.033569_fragment_1	40399	Yes	NA	Selenomonas	Selenomonadaceae
NODE_180_length_43747_cov_7.757301	43747	No	NA	Selenomonas	Selenomonadaceae
NODE_113_length_54559_cov_13.161731	54559	No	NA	NA	NA
NODE_199_length_67719_cov_8.881872	67719	No	NA	NA	NA
NODE_358_length_39523_cov_221.041831	39523	No	NA	Streptococcus	Streptococcaceae
NODE_245_length_57190_cov_7.700026	57190	No	NA	NA	NA
NODE_104_length_102435_cov_21.189764_fragment_1	42427	Yes	Siphoviridae	Bacillus,Enterococcus,Lactococcus	Bacillaceae,Enterococcaceae,Streptococcaceae
NODE_351_length_40398_cov_14.776244	40398	No	Siphoviridae	Corynebacterium	Corynebacteriaceae
NODE_74_length_123413_cov_15.220869_fragment_2	49218	Yes	Siphoviridae	Capnocytophaga,Riemerella	Flavobacteriaceae,Weeksellaceae
NODE_289_length_49982_cov_14.995473	49982	No	NA	NA	NA
NODE_5_length_525488_cov_55.067129_fragment_6_1	34803	Yes	NA	NA	NA
NODE_3572_length_5441_cov_1658.027850	5441	No	Microviridae	Enterobacteria,Escherichia	Enterobacteriaceae
NODE_389_length_37074_cov_27.840920	37074	No	NA	NA	NA
NODE_89_length_111054_cov_41.020739_fragment_1_1	41766	Yes	NA	Veillonella	Veillonellaceae
NODE_13_length_316449_cov_36.813543_fragment_2	32387	Yes	NA	NA	NA
NODE_237_length_59247_cov_11.249662_fragment_1	30262	Yes	Myoviridae	Pasteurella,Aeromonas,Haemophilus,Pseudomonas,Vibrio	Pasteurellaceae,Aeromonadaceae,Pseudomonadaceae,Vibrionaceae

NODE_207_length_42663_cov_9.025113	42663	No	NA	NA	NA
NODE_301_length_31898_cov_29.351663	31898	No	NA	NA	NA
NODE_247_length_36906_cov_7.044476	36906	No	Myoviridae	Haemophilus,Mannheimia,Vibrio	Pasteurellaceae,Vibrionaceae
NODE_117_length_65103_cov_22.632456	65103	No	Siphoviridae	Lactococcus	Streptococcaceae
NODE_141_length_57046_cov_62.313383_fragment_1_1	35085	Yes	NA	Neisseria	Neisseriaceae
NODE_46_length_122118_cov_56.900543_fragment_2_1	46231	Yes	NA	NA	NA
NODE_1709_length_5441_cov_149.728927	5441	No	Microviridae	Enterobacteria,Escherichia	Enterobacteriaceae
NODE_272_length_44620_cov_14.118120	44620	No	NA	NA	NA
NODE_309_length_40640_cov_7.898854_fragment_1	37099	Yes	Siphoviridae	Clostridium	Clostridiaceae
NODE_1071_length_14859_cov_58.353215	14859	No	Siphoviridae	Arthrobacter,Gordonia,Rhodococcus	Micrococcaceae,Gordoniaceae,Nocardiaceae
NODE_236_length_48188_cov_8.564914	48188	No	Myoviridae	Fusobacterium,Vibrio	Fusobacteriaceae,Vibrionaceae
NODE_46_length_133831_cov_21.720615_fragment_2	47955	Yes	Siphoviridae	Riemerella	Weeksellaceae
NODE_3381_length_5441_cov_835.097289	5441	No	Microviridae	Enterobacteria,Escherichia	Enterobacteriaceae
NODE_319_length_40614_cov_14.479943_1	37014	Yes	Myoviridae	Neisseria,Burkholderia,Enterobacteria,Erwinia,Escherichia,Mannheimia,Pseudomonas,Ralstonia,Salmonella,Stenotrophomonas,Yersinia	Neisseriaceae,Burkholderiaceae,Enterobacteriaceae,Erwiniaceae,Pasteurellaceae,Pseudomonadaceae,Xanthomonadaceae,Yersiniaceae
NODE_169_length_59035_cov_19.177755	59035	No	NA	NA	NA
NODE_100_length_77856_cov_108.844025_fragment_1	37915	Yes	Myoviridae	Neisseria,Haemophilus,Mannheimia,Vibrio	Neisseriaceae,Pasteurellaceae,Vibrionaceae
NODE_205_length_52609_cov_41.212867_fragment_1	37437	Yes	Siphoviridae	Streptococcus	Streptococcaceae
NODE_145_length_65237_cov_29.554524	65237	No	NA	NA	NA
NODE_380_length_37578_cov_20.086933	37578	No	NA	Leptotrichia	Leptotrichiaceae

NODE_111_length_75516_cov_117.274804_fragment_2	41064	Yes	NA	Neisseria	Neisseriaceae
NODE_322_length_40560_cov_17.789211	40560	No	NA	NA	NA
NODE_297_length_42715_cov_9.259752	42715	No	Myoviridae	Fusobacterium,Vibrio	Fusobacteriaceae,Vibrionaceae
NODE_476_length_32177_cov_11.950439	32177	No	Myoviridae	Pasteurella,Burkholderia,Enterobacteria,Erwinia,Escherichia,Mannheimia,Pseudomonas,Ralstonia,Salmonella,Stenotrophomonas,Yersinia	Pasteurellaceae,Burkholderiaceae,Enterobacteriaceae,Erwiniaceae,Pseudomonadaceae,Xanthomonadaceae,Yersiniaceae
NODE_309_length_41440_cov_53.240812	41440	No	NA	NA	NA
NODE_329_length_40147_cov_12.061184_1	33772	Yes	NA	NA	NA
NODE_518_length_29061_cov_4.418189	29061	No	NA	NA	NA
NODE_324_length_38320_cov_21.573657	38320	No	NA	NA	NA
NODE_194_length_51630_cov_6.447911	51630	No	Siphoviridae	Riemerella	Weeksellaceae
NODE_149_length_59955_cov_11.197479	59955	No	NA	NA	NA
NODE_46_length_168321_cov_6.799550	168321	No	NA	NA	NA
NODE_482_length_42751_cov_13.669149	42751	No	NA	Veillonella	Veillonellaceae
NODE_444_length_44971_cov_29.867753	44971	No	Siphoviridae	Streptococcus	Streptococcaceae
NODE_319_length_57450_cov_42.282899_1	42793	Yes	NA	Fusobacterium	Fusobacteriaceae
NODE_507_length_41255_cov_15.148568	41255	No	NA	NA	NA
NODE_209_length_74575_cov_29.893492_fragment_1	43849	Yes	NA	Neisseria	Neisseriaceae
NODE_51_length_162654_cov_67.765558_fragment_1	38492	Yes	NA	Veillonella	Veillonellaceae
NODE_512_length_41065_cov_15.295026_1	37883	Yes	NA	NA	NA
NODE_257_length_65740_cov_10.929162_fragment_1	35675	Yes	Myoviridae	Neisseria,Burkholderia,Enterobacteria,Erwinia,Escherichia,Mannheimia,Pseudomonas,Ralstonia,Salmonella,Stenotrophomonas,Yersinia	Neisseriaceae,Burkholderiaceae,Enterobacteriaceae,Erwiniaceae,Pasteurellaceae,Pseudomonadac

					eae,Xanthomonadaceae,Yersiniaceae
NODE_285_length_62393_cov_797.556258	62393	No	NA	NA	NA
NODE_190_length_39352_cov_24.587577	39352	No	NA	NA	NA
NODE_560_length_19333_cov_48.548071	19333	No	NA	NA	NA
NODE_124_length_51194_cov_12.101879_fragment_1	45657	Yes	NA	Neisseria	Neisseriaceae
NODE_125_length_51026_cov_19.241529	51026	No	NA	NA	NA
NODE_718_length_16395_cov_19.079437	16395	No	Podoviridae	Streptococcus,Enterococcus,Lactococcus	Streptococcaceae,Enterococcaceae
NODE_139_length_48251_cov_18.369512_fragment_1	41939	Yes	Siphoviridae	Streptococcus	Streptococcaceae
NODE_290_length_31341_cov_119.452311	31341	No	NA	Pseudopropionibacterium	Propionibacteriaceae
NODE_189_length_39404_cov_14.932730	39404	No	NA	NA	NA
NODE_381_length_37914_cov_18.110357	37914	No	NA	NA	NA
NODE_352_length_39602_cov_140.710218	39602	No	NA	NA	NA
NODE_901_length_19073_cov_12.352886	19073	No	Podoviridae	Actinomyces	Actinomycetaceae
NODE_399_length_36213_cov_22.159882	36213	No	NA	NA	NA
NODE_341_length_40940_cov_15.625339	40940	No	NA	Leptotrichia	Leptotrichiaceae
NODE_251_length_48597_cov_36.257282	48597	No	NA	NA	NA
NODE_432_length_33807_cov_32.909457	33807	No	Siphoviridae	Streptococcus	Streptococcaceae
NODE_204_length_55605_cov_32.080954	55605	No	NA	Neisseria	Neisseriaceae
NODE_15_length_227862_cov_21.629581	227862	No	Myoviridae	Halocynthia	Pyuridae
NODE_372_length_38364_cov_46.858023	38364	No	NA	NA	NA
NODE_283_length_37887_cov_8.682385	37887	No	Siphoviridae	Streptococcus	Streptococcaceae
NODE_214_length_44154_cov_23.156081_1	33731	Yes	Siphoviridae	Streptococcus	Streptococcaceae
NODE_5689_length_5441_cov_410.868177	5441	No	Microviridae	Enterobacteria,Escherichia	Enterobacteriaceae

NODE_176_length_49792_cov_10.446529_fragment_1	37563	Yes	NA	Neisseria	Neisseriaceae
NODE_244_length_40750_cov_11.225851_1	38955	Yes	Siphoviridae	Streptococcus	Streptococcaceae
NODE_110_length_66408_cov_14.166021	66408	No	NA	NA	NA
NODE_424_length_28396_cov_8.160545	28396	No	Myoviridae	Shewanella,Pasteurella,Aeromonas,Haemophilus,Pseudomonas,Vibrio	Shewanellaceae,Pasteurellaceae,Aeromonadaceae,Pseudomonadaceae,Vibrionaceae
NODE_252_length_39955_cov_11.209098	39955	No	NA	NA	NA
NODE_3563_length_7299_cov_5.139012	7299	No	Inoviridae	Pseudomonas	Pseudomonadaceae
NODE_175_length_45816_cov_141.124189	45816	No	Siphoviridae	Clavibacter,Microbacterium	Microbacteriaceae
NODE_32_length_128829_cov_7.805427_fragment_1	39835	Yes	Myoviridae	Pasteurella,Burkholderia,Enterobacteria,Erwinia,Escherichia,Mannheimia,Pseudomonas,Ralstonia,Salmonella,Stenotrophomonas,Yersinia	Pasteurellaceae,Burkholderiaceae,Enterobacteriaceae,Erwiniaceae,Pseudomonadaceae,Xanthomonadaceae,Yersiniaceae
NODE_118_length_57668_cov_7.029993_fragment_1	34871	Yes	NA	NA	NA
NODE_6_length_260214_cov_10.021506	260214	No	NA	NA	NA
NODE_170_length_46338_cov_10.682497	46338	No	NA	Fusobacterium	Fusobacteriaceae
NODE_192_length_42886_cov_20.894235	42886	No	Siphoviridae	Corynebacterium,Gordonia	Corynebacteriaceae,Gordoniaceae
NODE_158_length_48403_cov_32.550985	48403	No	Siphoviridae	Clavibacter,Microbacterium	Microbacteriaceae
NODE_145_length_51005_cov_60.787556	51005	No	Myoviridae	Leptotrichia,Vibrio	Leptotrichiaceae,Vibrionaceae
NODE_238_length_41683_cov_14.265302	41683	No	NA	Veillonella	Veillonellaceae
NODE_260_length_39116_cov_13.010420	39116	No	Myoviridae	Burkholderia,Enterobacteria,Erwinia,Escherichia,Mannheimia,Pseudomonas,Ralstonia,Salmonella,Stenotrophomonas,Yersinia	Burkholderiaceae,Enterobacteriaceae,Erwiniaceae,Pasteurellaceae,Pseudomonadaceae,Xanthomonadaceae,Yersiniaceae
NODE_286_length_36966_cov_12.832272	36966	No	NA	Neisseria	Neisseriaceae

NODE_89_length_82075_cov_105.638174_fragment_1_1	36605	Yes	Myoviridae	Haemophilus,Mannheimia	Pasteurellaceae
NODE_292_length_36393_cov_11.393995	36393	No	NA	Pseudopropionibacterium	Propionibacteriaceae
NODE_57_length_112911_cov_45.377003_fragment_1	57474	Yes	Siphoviridae	Riemerella	Weeksellaceae
NODE_1783_length_19209_cov_28.166440_1	12322	Yes	NA	NA	NA
NODE_608_length_38756_cov_229.950053	38756	No	NA	NA	NA
NODE_341_length_53638_cov_7.316518_1	48474	Yes	NA	NA	NA
NODE_58_length_114417_cov_30.683024_fragment_2_1	42461	Yes	NA	NA	NA
NODE_1040_length_27945_cov_27.997598	27945	No	NA	NA	NA
NODE_407_length_48233_cov_172.621902	48233	No	Siphoviridae	Clavibacter,Microbacterium	Microbacteriaceae
NODE_376_length_50408_cov_33.913928_1	45685	Yes	NA	NA	NA
NODE_440_length_46332_cov_6.428485_fragment_1	39423	Yes	NA	NA	NA
NODE_304_length_56250_cov_58.832672	56250	No	NA	NA	NA
NODE_15_length_194310_cov_70.064287_fragment_2	43722	Yes	NA	Leptotrichia	Leptotrichiaceae
NODE_319_length_55642_cov_35.982370	55642	No	NA	NA	NA
NODE_207_length_68309_cov_18.414672	68309	No	NA	NA	NA
NODE_514_length_43105_cov_22.140929	43105	No	NA	NA	NA
NODE_626_length_37778_cov_9.695703	37778	No	NA	NA	NA
NODE_1941_length_17904_cov_16.244000	17904	No	Podoviridae	Actinomyces	Actinomycetaceae
NODE_327_length_55095_cov_6.179924	55095	No	NA	NA	NA
NODE_115_length_92157_cov_7.470359_fragment_2	48186	Yes	NA	Prevotella	Bacteroidaceae
NODE_103_length_72395_cov_7.566243_fragment_1_1	39906	Yes	NA	NA	NA
NODE_326_length_41833_cov_9.021495	41833	No	NA	NA	NA
NODE_145_length_61116_cov_6.102258_fragment_2_1	7301	Yes	Microviridae	NA	NA
NODE_27_length_139417_cov_22.757229_fragment_1	90207	Yes	NA	NA	NA

NODE_415_length_35167_cov_25.911341	35167	No	Myoviridae	Neisseria,Haemophilus,Mannheimia,Vibrio	Neisseriaceae,Pasteurellaceae,Vibrionaceae
NODE_1699_length_12809_cov_5.690293	12809	No	NA	NA	NA
NODE_19_length_177112_cov_36.930633_fragment_1	48791	Yes	Siphoviridae	Riemerella	Weeksellaceae
NODE_277_length_43985_cov_9.668154	43985	No	NA	NA	NA
NODE_435_length_33550_cov_11.236752	33550	No	NA	NA	NA
NODE_290_length_42565_cov_12.468008	42565	No	NA	NA	NA
NODE_993_length_18992_cov_23.619520	18992	No	Podoviridae	Actinomyces	Actinomycetaceae
NODE_129_length_65583_cov_9.762956	65583	No	NA	NA	NA
NODE_1114_length_17532_cov_26.299937	17532	No	Podoviridae	Actinomyces	Actinomycetaceae
NODE_238_length_47610_cov_22.924971	47610	No	Siphoviridae	Riemerella	Weeksellaceae
NODE_155_length_72600_cov_11.550252_fragment_1	37559	Yes	Myoviridae	Burkholderia,Enterobacteria,Erwinia,Escherichia,Mannheimia,Pseudomonas,Ralstonia,Salmonella,Stenotrophomonas,Yersinia	Burkholderiaceae,Enterobacteriaceae,Erwiniaceae,Pasteurellaceae,Pseudomonadaceae,Xanthomonadaceae,Yersiniaceae
NODE_311_length_45925_cov_5.339416	45925	No	NA	NA	NA
NODE_131_length_80478_cov_11.086766_fragment_1	36876	Yes	Myoviridae	Haemophilus,Mannheimia,Vibrio	Pasteurellaceae,Vibrionaceae
NODE_443_length_34676_cov_81.665434	34676	No	NA	NA	NA
NODE_88_length_101901_cov_25.497918_fragment_1_1	44301	Yes	NA	Selenomonas	Selenomonadaceae
NODE_253_length_52896_cov_11.938760_1	43519	Yes	Podoviridae,Unassigned	Cardiobacterium,Bordetella,Burkholderia,Pseudomonas,Xanthomonas	Cardiobacteriaceae,Alcaligenaceae,Burkholderiaceae,Pseudomonadaceae,Xanthomonadaceae
NODE_264_length_51326_cov_8.554387_1	47016	Yes	Siphoviridae	Riemerella	Weeksellaceae
NODE_271_length_50219_cov_12.343613_1	45875	Yes	NA	NA	NA
NODE_323_length_44013_cov_25.626757	44013	No	NA	Selenomonas	Selenomonadaceae

NODE_136_length_78986_cov_13.619642_fragment_1	48221	Yes	Siphoviridae	Riemerella	Weeksellaceae
NODE_230_length_48166_cov_6.406539_fragment_1_1	36767	Yes	Siphoviridae	Streptococcus	Streptococcaceae
NODE_1127_length_17671_cov_16.242864	17671	No	Podoviridae	Actinomyces	Actinomycetaceae
NODE_206_length_51967_cov_42.864945_fragment_1	42078	Yes	NA	Fusobacterium	Fusobacteriaceae
NODE_286_length_42788_cov_15.372850_1	38751	Yes	Siphoviridae	Streptococcus	Streptococcaceae
NODE_260_length_45387_cov_10.652056	45387	No	NA	Neisseria	Neisseriaceae
NODE_359_length_37924_cov_26.639124	37924	No	NA	NA	NA
NODE_221_length_50179_cov_12.042993	50179	No	NA	NA	NA
NODE_243_length_46771_cov_23.999015	46771	No	NA	NA	NA
NODE_404_length_34954_cov_50.116106	34954	No	NA	NA	NA
NODE_353_length_38299_cov_11.918262	38299	No	NA	NA	NA
NODE_377_length_37023_cov_6.434917	37023	No	Siphoviridae	Streptococcus	Streptococcaceae
NODE_328_length_40828_cov_10.847988_fragment_1	36802	Yes	Myoviridae	Haemophilus,Mannheimia	Pasteurellaceae
NODE_288_length_43536_cov_11.628274	43536	No	NA	NA	NA
NODE_1258_length_17919_cov_60.977634	17919	No	Podoviridae	Actinomyces	Actinomycetaceae
NODE_70_length_95356_cov_32.563488	95356	No	NA	NA	NA
NODE_136_length_65639_cov_344.642184	65639	No	NA	NA	NA
NODE_343_length_40054_cov_22.252731	40054	No	NA	NA	NA
NODE_179_length_55195_cov_7.719859_fragment_1_1	6429	Yes	Microviridae	Parabacteroides	Tannerellaceae
NODE_320_length_41234_cov_11.669079	41234	No	NA	NA	NA
NODE_312_length_41781_cov_33.241840	41781	No	NA	NA	NA
NODE_1266_length_17829_cov_18.101376	17829	No	NA	NA	NA
NODE_476_length_33628_cov_29.263783	33628	No	NA	NA	NA
NODE_6_length_281831_cov_12.402969	281831	No	Myoviridae	Yersinia	Yersiniaceae
NODE_315_length_41493_cov_12.998842	41493	No	NA	NA	NA

NODE_15_length_201216_cov_14.831757_fragment_1	34666	Yes	NA	NA	NA
NODE_255_length_44602_cov_9.557770	44602	No	Podoviridae	Bordetella,Pseudomonas,Xanthomonas	Alcaligenaceae,Pseudomonadaceae,Xanthomonadaceae
NODE_29_length_142914_cov_35.467076_fragment_1_1	40166	Yes	Siphoviridae	Cellulophaga	Flavobacteriaceae
NODE_152_length_59891_cov_55.639080	59891	No	Podoviridae	Pseudomonas	Pseudomonadaceae
NODE_115_length_71127_cov_24.977502_fragment_1	38556	Yes	NA	NA	NA
NODE_124_length_66866_cov_7.170586_fragment_1_1	42821	Yes	NA	NA	NA
NODE_161_length_58291_cov_34.090648_fragment_1_1	38128	Yes	Myoviridae	Neisseria,Haemophilus,Mannheimia	Neisseriaceae,Pasteurellaceae
NODE_169_length_56930_cov_25.946796	56930	No	Siphoviridae	Clavibacter,Microbacterium	Microbacteriaceae
NODE_59_length_61862_cov_65.232708	61862	No	NA	NA	NA
NODE_173_length_40302_cov_37.726713	40302	No	NA	Actinomyces	Actinomycetaceae
NODE_194_length_38001_cov_10.054683	38001	No	Siphoviridae	Corynebacterium,Gordonia	Corynebacteriaceae,Gordoniaceae
NODE_47_length_67522_cov_48.407785	67522	No	NA	NA	NA
NODE_188_length_38691_cov_9.390853	38691	No	Siphoviridae	Corynebacterium	Corynebacteriaceae
NODE_72_length_56111_cov_11.155951_fragment_1	44750	Yes	NA	Neisseria	Neisseriaceae
NODE_49_length_66935_cov_12.366193	66935	No	Podoviridae	Alteromonas	Alteromonadaceae
NODE_418_length_48215_cov_15.282226	48215	No	NA	NA	NA
NODE_159_length_94931_cov_10.876523_fragment_1	39921	Yes	NA	NA	NA
NODE_86_length_132062_cov_8.564970_fragment_1	34463	Yes	NA	Pseudopropionibacterium	Propionibacteriaceae
NODE_150_length_96487_cov_10.799859	96487	No	NA	NA	NA
NODE_519_length_41821_cov_11.002083_1	38671	Yes	NA	Streptococcus	Streptococcaceae
NODE_368_length_52156_cov_82.400184_1	44968	Yes	NA	Fusobacterium	Fusobacteriaceae
NODE_527_length_41207_cov_9.271287_fragment_1	36719	Yes	NA	Streptococcus	Streptococcaceae
NODE_490_length_43877_cov_6.797225_fragment_1	35577	Yes	NA	NA	NA

NODE_157_length_95080_cov_106.320758_fragment_1_1	34584	Yes	Myoviridae	Neisseria,Haemophilus,Mannheimia	Neisseriaceae,Pasteurellaceae
NODE_423_length_47905_cov_142.236426	47905	No	Siphoviridae	Clavibacter,Microbacterium	Microbacteriaceae
NODE_337_length_55803_cov_10.873377_1	46246	Yes	NA	NA	NA
NODE_467_length_45375_cov_9.799735	45375	No	NA	NA	NA
NODE_181_length_53901_cov_24.360101	53901	No	NA	NA	NA
NODE_6_length_255783_cov_14.935756	255783	No	Myoviridae	Neisseria,Halocynthia	Neisseriaceae,Pyuridae
NODE_320_length_35490_cov_13.120474	35490	No	NA	NA	NA
NODE_253_length_41050_cov_60.441078	41050	No	NA	NA	NA
NODE_165_length_56820_cov_103.413265	56820	No	NA	NA	NA
NODE_163_length_57268_cov_67.079825	57268	No	NA	NA	NA
NODE_191_length_50526_cov_27.734224	50526	No	NA	NA	NA
NODE_244_length_42138_cov_110.490982_1	40446	Yes	Myoviridae	Neisseria,Burkholderia	Neisseriaceae,Burkholderiaceae
NODE_345_length_33991_cov_178.541696	33991	No	NA	NA	NA
NODE_251_length_41092_cov_36.655652_1	36861	Yes	NA	NA	NA
NODE_7_length_252440_cov_59.165133	252440	No	Myoviridae	Halocynthia	Pyuridae
NODE_281_length_37820_cov_22.005852	37820	No	NA	Streptococcus	Streptococcaceae
NODE_121_length_67416_cov_10.546102	67416	No	NA	Cardiobacterium	Cardiobacteriaceae
NODE_16_length_242573_cov_233.413652_fragment_1_1	36224	Yes	Myoviridae	Neisseria,Haemophilus,Mannheimia	Neisseriaceae,Pasteurellaceae
NODE_19_length_235687_cov_13.636433	235687	No	NA	NA	NA
NODE_331_length_54584_cov_30.267656_fragment_1_1	38356	Yes	NA	Leptotrichia	Leptotrichiaceae
NODE_95_length_112510_cov_37.065253_fragment_3	43538	Yes	NA	NA	NA
NODE_407_length_48198_cov_80.562740	48198	No	Siphoviridae	Clavibacter,Microbacterium	Microbacteriaceae
NODE_62_length_143471_cov_6.398526_fragment_1_1	34748	Yes	NA	NA	NA
NODE_418_length_47079_cov_7.459744	47079	No	NA	NA	NA
NODE_555_length_39048_cov_7.641474	39048	No	NA	Leptotrichia	Leptotrichiaceae

NODE_544_length_39450_cov_28.069197	39450	No	NA	NA	NA
NODE_1536_length_17986_cov_19.270717	17986	No	NA	NA	NA
NODE_105_length_104526_cov_10.167597_fragment_2	32168	Yes	NA	NA	NA
NODE_8_length_345597_cov_89.928512_fragment_5	43153	Yes	Podoviridae	Bordetella,Burkholderia	Alcaligenaceae,Burkholderiaceae
NODE_129_length_91691_cov_33.618916_fragment_1	46221	Yes	NA	NA	NA
NODE_545_length_39445_cov_36.429094_1	37684	Yes	Myoviridae	Haemophilus,Mannheimia	Pasteurellaceae
NODE_434_length_45791_cov_23.294604_1	41199	Yes	NA	Fusobacterium	Fusobacteriaceae
NODE_88_length_91520_cov_7.043886	91520	No	NA	NA	NA
NODE_337_length_35262_cov_9.426677	35262	No	Siphoviridae	Streptococcus	Streptococcaceae
NODE_276_length_40802_cov_7.998577	40802	No	NA	NA	NA
NODE_251_length_43696_cov_11.302949_1	37867	Yes	NA	Neisseria	Neisseriaceae
NODE_181_length_55225_cov_9.161138	55225	No	Myoviridae	Neisseria,Burkholderia,Enterobacteria,Erwinia,Escherichia,Mannheimia,Pseudomonas,Ralstonia,Salmonella,Stenotrophomonas,Yersinia	Neisseriaceae,Burkholderiaceae,Enterobacteriaceae,Erwiniaceae,Pasteurellaceae,Pseudomonadaceae,Xanthomonadaceae,Yersiniaceae
NODE_257_length_43064_cov_10.273780	43064	No	NA	NA	NA
NODE_326_length_36186_cov_13.473084	36186	No	NA	NA	NA
NODE_262_length_42207_cov_15.724473	42207	No	NA	NA	NA
NODE_139_length_65930_cov_22.906019	65930	No	NA	NA	NA
NODE_479_length_39537_cov_13.237425	39537	No	NA	Streptococcus	Streptococcaceae
NODE_135_length_91309_cov_12.661966	91309	No	NA	NA	NA
NODE_507_length_38141_cov_11.986767	38141	No	NA	NA	NA
NODE_1329_length_18725_cov_62.123110	18725	No	Podoviridae	Streptococcus	Streptococcaceae

NODE_499_length_38727_cov_14.693758_1	33899	Yes	NA	NA	NA
NODE_410_length_43854_cov_25.510560_fragment_1	37708	Yes	NA	Leptotrichia	Leptotrichiaceae
NODE_576_length_34596_cov_10.544773	34596	No	Myoviridae	Aggregatibacter,Haemophilus,Mannheimia	Pasteurellaceae
NODE_450_length_41172_cov_10.445728	41172	No	Myoviridae	Haemophilus,Aggregatibacter	Pasteurellaceae
NODE_483_length_39343_cov_58.153787	39343	No	NA	Pseudopropionibacterium	Propionibacteriaceae
NODE_452_length_41109_cov_16.603863	41109	No	NA	NA	NA
NODE_470_length_40234_cov_69.698176	40234	No	NA	Neisseria	Neisseriaceae
NODE_383_length_44006_cov_10.163341_1	37847	Yes	Siphoviridae	Streptococcus	Streptococcaceae
NODE_412_length_41288_cov_23.602454	41288	No	NA	NA	NA
NODE_323_length_48297_cov_18.929874	48297	No	Siphoviridae	Clavibacter,Microbacterium	Microbacteriaceae
NODE_385_length_43782_cov_84.393029	43782	No	Myoviridae	Haemophilus,Mannheimia,Vibrio	Pasteurellaceae,Vibrionaceae
NODE_428_length_40339_cov_11.730712_1	35094	Yes	Myoviridae	Neisseria,Burkholderia,Enterobacteria,Erwinia,Escherichia,Mannheimia,Pseudomonas,Ralstonia,Salmonella,Stenotrophomonas,Yersinia	Neisseriaceae,Burkholderiaceae,Enterobacteriaceae,Erwiniaceae,Pasteurellaceae,Pseudomonadaceae,Xanthomonadaceae,Yersiniaceae
NODE_478_length_37213_cov_13.380564_1	34348	Yes	Myoviridae	Aggregatibacter,Haemophilus,Mannheimia,Vibrio	Pasteurellaceae,Vibrionaceae
NODE_1275_length_19348_cov_65.506829	19348	No	Podoviridae	Actinomyces	Actinomycetaceae
NODE_457_length_38880_cov_10.493368_1	33950	Yes	Myoviridae	Aggregatibacter,Haemophilus,Mannheimia,Vibrio	Pasteurellaceae,Vibrionaceae
NODE_548_length_33977_cov_16.382259	33977	No	NA	Fusobacterium	Fusobacteriaceae
NODE_461_length_38233_cov_66.904343	38233	No	Siphoviridae	Corynebacterium,Gordonia	Corynebacteriaceae,Gordoniaceae
NODE_522_length_35038_cov_13.568076_1	30426	Yes	NA	NA	NA
NODE_263_length_56046_cov_6.702024_fragment_1_1	45930	Yes	Siphoviridae	Riemerella	Weeksellaceae

NODE_284_length_53014_cov_22.734908_1	43026	Yes	Podoviridae	Bordetella,Pseudomonas,Xanthomonas	Alcaligenaceae,Pseudomonadaceae,Xanthomonadaceae
NODE_380_length_44302_cov_20.020905_fragment_1	36752	Yes	NA	NA	NA
NODE_4_length_367028_cov_9.960730_fragment_1	33819	Yes	NA	NA	NA
NODE_456_length_30981_cov_28.204165	30981	No	NA	Pseudopropionibacterium	Propionibacteriaceae
NODE_215_length_54921_cov_11.230780	54921	No	NA	NA	NA
NODE_208_length_55392_cov_41.643710	55392	No	NA	NA	NA
NODE_291_length_42934_cov_8.470370_fragment_1	37757	Yes	NA	NA	NA
NODE_292_length_42619_cov_21.377714	42619	No	NA	Neisseria	Neisseriaceae
NODE_391_length_34664_cov_7.298766	34664	No	Myoviridae	Shewanella,Aeromonas,Haemophilus,Pasteurella,Pseudomonas,Vibrio	Shewanellaceae,Aeromonadaceae,Pasteurellaceae,Pseudomonadaceae,Vibrionaceae
NODE_310_length_41332_cov_8.260048	41332	No	NA	NA	NA
NODE_330_length_39976_cov_28.669998	39976	No	Siphoviridae	Rhodococcus,Streptomyces	Nocardiaceae,Streptomycetaceae
NODE_200_length_56785_cov_26.176274_fragment_1	39774	Yes	NA	NA	NA
NODE_316_length_40660_cov_7.841054	40660	No	NA	NA	NA
NODE_51_length_136339_cov_31.045082_fragment_3_1	38924	Yes	NA	NA	NA
NODE_450_length_31436_cov_6.923744	31436	No	NA	Fusobacterium	Fusobacteriaceae
NODE_91_length_98822_cov_8.033959_fragment_1	41208	Yes	NA	NA	NA
NODE_187_length_62357_cov_55.548185	62357	No	NA	NA	NA
NODE_1175_length_19070_cov_15.337255	19070	No	NA	NA	NA
NODE_333_length_42587_cov_7.335935	42587	No	NA	NA	NA
NODE_275_length_47868_cov_11.325999	47868	No	NA	NA	NA
NODE_82_length_97251_cov_19.616486	97251	No	NA	NA	NA

NODE_99_length_88750_cov_29.613169_fragment_1_1	35716	Yes	NA	NA	NA
NODE_241_length_53134_cov_15.517493_1	42602	Yes	NA	NA	NA
NODE_1305_length_17505_cov_53.469305	17505	No	Podoviridae	Actinomyces	Actinomycetaceae
NODE_194_length_60668_cov_12.035058	60668	No	NA	NA	NA
NODE_96_length_89874_cov_11.036852	89874	No	Unassigned	Cellulophaga	Flavobacteriaceae
NODE_361_length_40630_cov_11.412495	40630	No	NA	NA	NA
NODE_11_length_202686_cov_8.870938	202686	No	NA	NA	NA
NODE_237_length_40882_cov_9.475886	40882	No	NA	NA	NA
NODE_115_length_71832_cov_29.743191_fragment_1	52928	Yes	NA	NA	NA
NODE_10_length_334020_cov_138.241498_fragment_2	42289	Yes	Myoviridae	Neisseria,Haemophilus,Mannheimia	Neisseriaceae,Pasteurellaceae
NODE_653_length_17534_cov_10.852351	17534	No	Podoviridae	Actinomyces	Actinomycetaceae
NODE_235_length_40906_cov_90.275220	40906	No	NA	NA	NA
NODE_29_length_177132_cov_9.222372_fragment_1	54825	Yes	NA	NA	NA
NODE_15_length_223415_cov_80.481160_fragment_2	46309	Yes	Myoviridae	Vibrio	Vibrionaceae
NODE_22_length_192697_cov_81.569071_fragment_1	37553	Yes	NA	NA	NA
NODE_452_length_41458_cov_14.752192_fragment_1	31185	Yes	Myoviridae	Pasteurella,Aeromonas,Haemophilus,Pseudomonas,Vibrio	Pasteurellaceae,Aeromonadaceae,Pseudomonadaceae,Vibrionaceae
NODE_104_length_91672_cov_24.432147_fragment_1_1	36780	Yes	Siphoviridae	Streptococcus	Streptococcaceae
NODE_90_length_98236_cov_30.927216	98236	No	NA	NA	NA
NODE_443_length_42074_cov_13.669673	42074	No	Siphoviridae	Bacillus,Croceibacter,Enterococcus,Lactococcus	Bacillaceae,Flavobacteriaceae,Enterococcaceae,Streptococcaceae
NODE_329_length_49391_cov_7.528701	49391	No	NA	NA	NA
NODE_701_length_30523_cov_18.954017	30523	No	Siphoviridae	Streptococcus	Streptococcaceae

NODE_426_length_42694_cov_13.926218	42694	No	NA	NA	NA
NODE_455_length_41279_cov_17.348171_fragment_1	33597	Yes	NA	NA	NA
NODE_152_length_75050_cov_7.478179_fragment_1_1	44329	Yes	NA	Campylobacter	Campylobacteraceae
NODE_323_length_49777_cov_10.125377_fragment_1	41058	Yes	Myoviridae	Burkholderia,Enterobacteria,Erwinia,Escherichia,Mannheimia,Pseudomonas,Ralstonia,Salmonella,Stenotrophomonas,Yersinia	Burkholderiaceae,Enterobacteriaceae,Erwiniaceae,Pasteurellaceae,Pseudomonadaceae,Xanthomonadaceae,Yersiniaceae
NODE_36_length_152114_cov_163.488139	152114	No	NA	NA	NA
NODE_432_length_42270_cov_8.581334	42270	No	NA	NA	NA
NODE_250_length_56061_cov_14.749491	56061	No	Siphoviridae	Arthrobacter	Micrococcaceae
NODE_437_length_42184_cov_8.468846	42184	No	NA	NA	NA
NODE_277_length_53699_cov_25.725337_1	47272	Yes	NA	NA	NA
NODE_615_length_33590_cov_4.541822	33590	No	NA	NA	NA
NODE_390_length_44563_cov_10.526557_fragment_1_1	33829	Yes	NA	NA	NA
NODE_5_length_499443_cov_75.328598_fragment_4	34307	Yes	NA	Haemophilus	Pasteurellaceae
NODE_1783_length_15471_cov_17.451155	15471	No	Siphoviridae	Arthrobacter,Gordonia,Rhodococcus	Micrococcaceae,Gordoniaceae,Nocardiaceae
NODE_73_length_109893_cov_25.790537_fragment_1	36461	Yes	Myoviridae	Haemophilus,Mannheimia,Vibrio	Pasteurellaceae,Vibrionaceae
NODE_225_length_59936_cov_488.577629_fragment_1	38905	Yes	NA	Neisseria	Neisseriaceae
NODE_556_length_36329_cov_8.750924	36329	No	NA	NA	NA
NODE_594_length_34620_cov_24.053812_fragment_1	29968	Yes	Myoviridae	Mannheimia,Pasteurella,Aeromonas,Haemophilus,Pseudomonas,Vibrio	Pasteurellaceae,Aeromonadaceae,Pseudomonadaceae,Vibrionaceae
NODE_112_length_87554_cov_42.403456	87554	No	Siphoviridae	Cellulophaga	Flavobacteriaceae
NODE_97_length_94834_cov_35.603857_fragment_2	49570	Yes	Siphoviridae	Riemerella	Weeksellaceae

NODE_368_length_46279_cov_8.642134_1	44541	Yes	NA	Neisseria	Neisseriaceae
NODE_510_length_38692_cov_5.890778	38692	No	Siphoviridae	Pseudomonas	Pseudomonadaceae
NODE_205_length_63340_cov_10.525433_fragment_1	42478	Yes	NA	Selenomonas	Selenomonadaceae
NODE_487_length_39369_cov_18.708831	39369	No	NA	NA	NA
NODE_333_length_49274_cov_14.941649_1	41857	Yes	NA	NA	NA
NODE_1323_length_19344_cov_35.489917_1	13404	Yes	NA	NA	NA
NODE_576_length_35619_cov_7.477758	35619	No	NA	Neisseria	Neisseriaceae
NODE_469_length_40504_cov_27.721501_fragment_1	35562	Yes	NA	NA	NA
NODE_291_length_54157_cov_36.176056_fragment_1	43603	Yes	NA	Leptotrichia	Leptotrichiaceae
NODE_199_length_68937_cov_108.746320	68937	No	NA	NA	NA
NODE_641_length_31542_cov_31.681202	31542	No	Myoviridae	Burkholderia,Enterobacteria,Erwinia,Escherichia,Mannheimia,Pseudomonas,Ralstonia,Salmonella,Stenotrophomonas,Yersinia	Burkholderiaceae,Enterobacteriaceae,Erwiniaceae,Pasteurellaceae,Pseudomonadaceae,Xanthomonadaceae,Yersiniaceae
NODE_52_length_155770_cov_8.270815_fragment_3	34956	Yes	Siphoviridae	Bacillus,Lactobacillus,Streptococcus	Bacillaceae,Lactobacillaceae,Streptococcaceae
NODE_560_length_34787_cov_14.425515	34787	No	NA	Neisseria	Neisseriaceae
NODE_428_length_41428_cov_199.873275	41428	No	NA	NA	NA
NODE_184_length_72224_cov_13.060705_fragment_1_1	35296	Yes	Myoviridae	Pasteurella,Burkholderia,Enterobacteria,Erwinia,Escherichia,Mannheimia,Pseudomonas,Ralstonia,Salmonella,Stenotrophomonas,Yersinia	Pasteurellaceae,Burkholderiaceae,Enterobacteriaceae,Erwiniaceae,Pseudomonadaceae,Xanthomonadaceae,Yersiniaceae
NODE_513_length_36679_cov_10.906537	36679	No	NA	Neisseria	Neisseriaceae
NODE_399_length_43783_cov_32.804862	43783	No	NA	NA	NA
NODE_483_length_46000_cov_9.185831_1	42414	Yes	NA	NA	NA

NODE_564_length_40808_cov_15.168797_1	34872	Yes	NA	NA	NA
NODE_208_length_77813_cov_13.708905	77813	No	Myoviridae	Bacillus	Bacillaceae
NODE_1402_length_19288_cov_421.875614	19288	No	Podoviridae	Actinomyces	Actinomycetaceae
NODE_458_length_47853_cov_12.149358	47853	No	Myoviridae	Leptotrichia,Vibrio	Leptotrichiaceae,Vibrionaceae
NODE_11_length_422586_cov_33.461848_fragment_1_1	41314	Yes	Myoviridae	Haemophilus,Aggregatibacter	Pasteurellaceae
NODE_298_length_63060_cov_14.762225_fragment_1_1	39463	Yes	NA	Leptotrichia	Leptotrichiaceae
NODE_634_length_37289_cov_19.191223	37289	No	NA	Veillonella	Veillonellaceae
NODE_685_length_35640_cov_26.712660	35640	No	Siphoviridae	Clostridium	Clostridiaceae
NODE_498_length_44541_cov_12.450569	44541	No	NA	NA	NA
NODE_519_length_43356_cov_19.019746	43356	No	NA	NA	NA
NODE_745_length_33486_cov_20.619754	33486	No	Myoviridae	Neisseria,Burkholderia,Enterobacteria,Erwinia,Escherichia,Mannheimia,Pseudomonas,Ralstonia,Salmonella,Stenotrophomonas,Yersinia	Neisseriaceae,Burkholderiaceae,Enterobacteriaceae,Erwiniaceae,Pasteurellaceae,Pseudomonadaceae,Xanthomonadaceae,Yersiniaceae
NODE_473_length_46503_cov_20.567495_1	40802	Yes	NA	NA	NA
NODE_644_length_36905_cov_86.862605	36905	No	NA	Neisseria	Neisseriaceae
NODE_565_length_40756_cov_8.943146_1	37121	Yes	NA	NA	NA
NODE_227_length_46776_cov_16.046339	46776	No	NA	NA	NA
NODE_10_length_259928_cov_95.780104	259928	No	NA	NA	NA
NODE_308_length_36994_cov_9.232356	36994	No	NA	NA	NA
NODE_337_length_34040_cov_20.954892	34040	No	NA	NA	NA
NODE_178_length_56923_cov_93.534589_fragment_1	40109	Yes	NA	NA	NA
NODE_262_length_42872_cov_54.000257	42872	No	NA	NA	NA

NODE_572_length_41697_cov_7.441309	41697	No	Podoviridae	Campylobacter,Edwardsiella,Pseudoalteromonas,Vibrio	Campylobacteraceae,Enterobacteriaceae,Pseudoalteromonadaceae,Vibrionaceae
NODE_621_length_39377_cov_272.890392	39377	No	Siphoviridae	Corynebacterium	Corynebacteriaceae
NODE_38_length_197681_cov_23.702094_fragment_2	47401	Yes	Siphoviridae	Capnocytophaga,Riemerella	Flavobacteriaceae,Weeksellaceae
NODE_438_length_49742_cov_10.897337	49742	No	NA	NA	NA
NODE_2791_length_7506_cov_117.160918	7506	No	Inoviridae	Pseudomonas	Pseudomonadaceae
NODE_67_length_145830_cov_36.864895_fragment_2	41918	Yes	NA	NA	NA
NODE_103_length_121323_cov_205.831530_fragment_1_1	35969	Yes	Myoviridae	Neisseria,Aggregatibacter,Haemophilus,Mannheimia	Neisseriaceae,Pasteurellaceae
NODE_143_length_105773_cov_8.996245_fragment_1	36264	Yes	NA	NA	NA
NODE_424_length_50548_cov_50.542372_1	41729	Yes	Myoviridae	Burkholderia,Enterobacteria,Erwinia,Escherichia,Mannheimia,Pseudomonas,Ralstonia,Salmonella,Stenotrophomonas,Yersinia	Burkholderiaceae,Enterobacteriaceae,Erwiniaceae,Pasteurellaceae,Pseudomonadaceae,Xanthomonadaceae,Yersiniaceae
NODE_417_length_51237_cov_26.358544_fragment_1	43461	Yes	NA	Neisseria	Neisseriaceae
NODE_171_length_92038_cov_49.420610_fragment_1	37157	Yes	NA	NA	NA
NODE_49_length_167623_cov_36.401962_fragment_1	43689	Yes	Myoviridae	Leptotrichia,Clostridium	Leptotrichiaceae,Clostridiaceae
NODE_114_length_69864_cov_33.770230_fragment_1	35810	Yes	Myoviridae	Burkholderia,Enterobacteria,Erwinia,Escherichia,Mannheimia,Pseudomonas,Ralstonia,Salmonella,Stenotrophomonas,Yersinia	Burkholderiaceae,Enterobacteriaceae,Erwiniaceae,Pasteurellaceae,Pseudomonadaceae,Xanthomonadaceae,Yersiniaceae
NODE_71_length_88569_cov_13.313329_fragment_1	40547	Yes	Myoviridae	Aggregatibacter,Haemophilus	Pasteurellaceae
NODE_342_length_41981_cov_12.291609	41981	No	NA	NA	NA
NODE_455_length_35506_cov_11.225974	35506	No	NA	NA	NA
NODE_1297_length_18084_cov_6.355483	18084	No	NA	NA	NA

NODE_426_length_37037_cov_8.300552	37037	No	NA	NA	NA
NODE_186_length_56175_cov_5.977833	56175	No	NA	NA	NA
NODE_64_length_95140_cov_6.618489_fragment_1_1	36164	Yes	NA	NA	NA
NODE_1329_length_17820_cov_52.436755	17820	No	NA	NA	NA
NODE_42_length_106102_cov_23.731544_fragment_1	67874	Yes	Siphoviridae	Vibrio	Vibrionaceae
NODE_284_length_46544_cov_27.412743_fragment_1	36198	Yes	NA	NA	NA
NODE_31_length_120227_cov_7.293588	120227	No	Siphoviridae	Selenomonas,Clostridium,Streptococcus	Selenomonadaceae,Clostridiaceae,Streptococcaceae
NODE_89_length_77610_cov_6.824447	77610	No	Myoviridae	Bacillus	Bacillaceae
NODE_268_length_47952_cov_31.391214	47952	No	NA	NA	NA
NODE_199_length_54438_cov_19.129084	54438	No	NA	NA	NA
NODE_9_length_185844_cov_15.980365	185844	No	NA	NA	NA
NODE_12_length_151086_cov_434.513199_fragment_3_1	35716	Yes	Myoviridae	Haemophilus,Mannheimia	Pasteurellaceae
NODE_157_length_35049_cov_20.128822	35049	No	NA	NA	NA
NODE_15_length_127752_cov_377.247985_fragment_1	31973	Yes	Myoviridae	Rhizobium	Rhizobiaceae
NODE_58_length_63499_cov_486.373306	63499	No	Siphoviridae	Lactococcus	Streptococcaceae
NODE_131_length_38306_cov_618.647983	38306	No	NA	NA	NA
NODE_124_length_39732_cov_859.951634	39732	No	Myoviridae	Neisseria,Rhizobium	Neisseriaceae,Rhizobiaceae
NODE_148_length_36551_cov_15.714626	36551	No	NA	NA	NA
NODE_165_length_33970_cov_21.830576	33970	No	Siphoviridae	Streptococcus	Streptococcaceae
NODE_66_length_60548_cov_1487.066851	60548	No	NA	NA	NA
NODE_44_length_71113_cov_167.850376_fragment_1	57602	Yes	NA	NA	NA
NODE_51_length_67245_cov_851.181307	67245	No	NA	Streptococcus	Streptococcaceae
NODE_503_length_35345_cov_30.689345	35345	No	NA	NA	NA
NODE_32_length_152269_cov_23.005505_fragment_2	56689	Yes	NA	Prevotella	Bacteroidaceae

NODE_291_length_49109_cov_53.934011_1	41991	Yes	NA	NA	NA
NODE_353_length_43779_cov_66.822706	43779	No	NA	NA	NA
NODE_1874_length_14050_cov_53.986924	14050	No	Siphoviridae	Arthrobacter,Gordonia,Rhodococcus	Micrococcaceae,Gordoniaceae, Nocardiaceae
NODE_72_length_104719_cov_6.301049_fragment_2_1	45360	Yes	NA	NA	NA
NODE_11_length_232803_cov_36.717978_fragment_1_1	39126	Yes	NA	Streptococcus	Streptococcaceae
NODE_235_length_55248_cov_28.355063_fragment_1	45851	Yes	NA	Fusobacterium	Fusobacteriaceae
NODE_436_length_38824_cov_9.768475	38824	No	NA	NA	NA
NODE_466_length_37272_cov_10.531263	37272	No	NA	Streptococcus	Streptococcaceae
NODE_205_length_60005_cov_19.105154_fragment_1	49874	Yes	Siphoviridae	Riemerella	Weeksellaceae
NODE_333_length_42412_cov_119.863423	42412	No	NA	Corynebacterium	Corynebacteriaceae
NODE_26_length_168502_cov_80.192144_fragment_1_1	36074	Yes	NA	NA	NA
NODE_547_length_31311_cov_10.792072	31311	No	NA	Pseudopropionibacterium	Propionibacteriaceae
NODE_211_length_56228_cov_125.504014	56228	No	NA	NA	NA
NODE_266_length_48730_cov_13.023770_fragment_1_1	36369	Yes	NA	Fusobacterium	Fusobacteriaceae
NODE_210_length_56495_cov_14.375354	56495	No	Siphoviridae	Streptococcus,Streptomyces	Streptococcaceae,Streptomyceta ceae
NODE_1918_length_13284_cov_14.755084	13284	No	NA	NA	NA
NODE_223_length_55231_cov_64.528998	55231	No	NA	NA	NA
NODE_406_length_37567_cov_7.333120_1	34457	Yes	NA	NA	NA
NODE_218_length_55389_cov_104.720461	55389	No	NA	NA	NA
NODE_431_length_36231_cov_27.391641	36231	No	Siphoviridae	Streptococcus	Streptococcaceae
NODE_1236_length_17921_cov_61.863874	17921	No	Podoviridae	Actinomyces	Actinomycetaceae
NODE_222_length_55241_cov_21.640470_fragment_1	37312	Yes	NA	NA	NA
NODE_351_length_41276_cov_7.159118	41276	No	NA	NA	NA

NODE_200_length_57679_cov_7.967514_fragment_1	45999	Yes	NA	NA	NA
NODE_93_length_97930_cov_23.218074_fragment_1	38205	Yes	Siphoviridae	Streptococcus	Streptococcaceae
NODE_93_length_97930_cov_23.218074_fragment_2	41705	Yes	Siphoviridae	Streptococcus	Streptococcaceae
NODE_224_length_54971_cov_16.342286	54971	No	Siphoviridae	Streptomyces	Streptomycetaceae
NODE_416_length_44993_cov_10.982465_fragment_1	35786	Yes	NA	NA	NA
NODE_473_length_39896_cov_17.092543	39896	No	NA	Veillonella	Veillonellaceae
NODE_474_length_39891_cov_10.179662	39891	No	NA	NA	NA
NODE_519_length_37028_cov_8.331972	37028	No	NA	Neisseria	Neisseriaceae
NODE_360_length_50024_cov_11.310152	50024	No	NA	NA	NA
NODE_374_length_48979_cov_13.184204	48979	No	Siphoviridae	Clavibacter,Microbacterium	Microbacteriaceae
NODE_502_length_37951_cov_9.979127	37951	No	NA	Listeria	Listeriaceae
NODE_122_length_97864_cov_82.725260_fragment_1_1	33086	Yes	NA	NA	NA
NODE_539_length_35996_cov_29.603851	35996	No	NA	NA	NA
NODE_450_length_42333_cov_8.856001_1	36177	Yes	Myoviridae	Burkholderia,Enterobacteria,Erwinia,Escherichia,Mannheimia,Pseudomonas,Ralstonia,Salmonella,Stenotrophomonas,Yersinia	Burkholderiaceae,Enterobacteriaceae,Erwiniaceae,Pasteurellaceae,Pseudomonadaceae,Xanthomonadaceae,Yersiniaceae
NODE_451_length_42209_cov_27.950799	42209	No	Siphoviridae	Cellulophaga	Flavobacteriaceae
NODE_514_length_37172_cov_5.794515_1	35061	Yes	NA	NA	NA
NODE_1383_length_16121_cov_5.070273	16121	No	Podoviridae	Actinomyces	Actinomycetaceae
NODE_483_length_39015_cov_30.086268	39015	No	Myoviridae	Haemophilus,Mannheimia	Pasteurellaceae
NODE_558_length_35218_cov_7.175355	35218	No	NA	NA	NA
NODE_1761_length_13133_cov_19.956109	13133	No	NA	NA	NA
NODE_434_length_43733_cov_499.535533	43733	No	NA	NA	NA
NODE_585_length_34009_cov_41.865524	34009	No	NA	Neisseria	Neisseriaceae

NODE_218_length_41641_cov_23.256312	41641	No	NA	NA	NA
NODE_241_length_38835_cov_198.392599	38835	No	NA	Corynebacterium	Corynebacteriaceae
NODE_156_length_52031_cov_21.665038	52031	No	NA	NA	NA
NODE_293_length_33663_cov_8.384998	33663	No	NA	NA	NA

Supplementary Table 4. Phages shared by both oral and gut microbiome

cluster	phage-genome	site	length	identity	host
1	NODE_292_length_65851_cov_9.764530_fragment_1_1	gut	38353nt	100.00%	unknown
	NODE_473_length_46503_cov_20.567495_1	oral	40802nt		
2	NODE_564_length_37071_cov_9.906014	gut	37071nt	99.82%	unknown
	NODE_327_length_40678_cov_9.074834	oral	40678nt		
3	NODE_465_length_44099_cov_9.663632_1	gut	35456nt	98.17%	Veillonella
	NODE_634_length_37289_cov_19.191223	oral	37289nt		
4	NODE_4413_length_5441_cov_750.485704	gut	5441nt	96.75%	Escherichia
	NODE_2935_length_5441_cov_334.768288	oral	5441nt		
5	NODE_4413_length_5441_cov_750.485704	gut	5441nt	98.22%	Escherichia
	NODE_3572_length_5441_cov_1658.027850	oral	5441nt		
6	NODE_3943_length_5441_cov_143.533049	gut	5441nt	97.19%	Escherichia
	NODE_1709_length_5441_cov_149.728927	oral	5441nt		
7	NODE_4508_length_5441_cov_1028.350910	gut	5441nt	96.54%	Escherichia
	NODE_3659_length_5441_cov_495.210917	oral	5441nt		
8	NODE_3887_length_5441_cov_866.652247	gut	5441nt	99.49%	Escherichia
	NODE_5689_length_5441_cov_410.868177	oral	5441nt		
9	NODE_4003_length_5441_cov_782.383773	gut	5441nt	99.96%	Escherichia
	NODE_3138_length_5441_cov_542.283141	oral	5441nt		
10	NODE_7842_length_5441_cov_846.019309	gut	5441nt	98.24%	Escherichia
	NODE_3381_length_5441_cov_835.097289	oral	5441nt		
11	NODE_6293_length_5441_cov_862.167843	gut	5441nt	98.79%	Escherichia
	NODE_5131_length_5441_cov_728.279057	oral	5441nt		

Supplementary Table 5. Differential phages in ASD and HC oral community identified by quasi-paired cohort approach using phage proportion (Wilcoxon signed-rank test)

Phage-host family	p.value	FDR	HC-mean	ASD-mean	HC-median	ASD-median	log2(FC)	Enriched in
Streptococcaceae	4.48E-06	3.25E-05	0.0272449	0.0473794	0.005980219	0.01629448	1.446113	ASD
Veillonellaceae	0.000367862	0.001524	0.0053295	0.0105053	0.001366971	0.003529867	1.368631	ASD
Corynebacteriaceae	0.005263972	0.013605	0.0257197	0.0401448	0.008417463	0.010129344	0.267083	ASD
Selenomonadaceae	0.019118898	0.034653	0.0081089	0.0043617	0.000885056	0.001122416	0.342767	ASD
Leptotrichiaceae	0.026386235	0.045012	0.0265402	0.0316505	0.014556576	0.021123883	0.537204	ASD
Pasteurellaceae	0.003214698	0.009323	0.0099427	0.0089571	0.003149653	0.00223365	-0.49579	HC
Fusobacteriaceae	0.011059569	0.022909	0.0244591	0.0158912	0.020986659	0.009467634	-1.1484	HC
Pseudomonadaceae	0.017478437	0.033792	0.004786988	0.002159998	0.001565342	0.001229037	-0.34895	HC

Supplementary Table 6. Differential phages in ASD and HC gut community identified by quasi-paired cohort approach using phage proportion (Wilcoxon signed-rank test)

Phage-host family	p.value	FDR	HC-mean	ASD-mean	HC-median	ASD-median	Enriched in
Bifidobacteriaceae	3.08E-05	0.00012326	0.0109368	0.018031713	0.003158275	0.007065124	ASD
Erysipelotrichaceae	0.0043776	0.01167354	0.0052857	0.011269972	0.003658617	0.003480755	ASD
Clostridiaceae	0.0133238	0.03197712	0.0251215	0.028536991	0.015145209	0.019071579	ASD
Vibrionaceae	0.021199	0.0462524	0.0561268	0.064208642	0.036296977	0.057388747	ASD
Bacillaceae	0.000013	0.0000625	0.0081845	0.004904921	0.005294616	0.003270495	HC
Streptococcaceae	0.6222991	0.67887179	2.51E-06	3.1E-09	0	0	no significant difference

Supplementary Table 7. Correlations between *Streptococcus* spp., Streptococcal phages, Streptococcal VFs and clinical manifestations of autism (Spearman’s rank correlation coefficient)

Feature	Rating Scale	rho	p-value
Streptococcus spp.	GI	0.023535	0.909143
Streptococcus spp.	CARS	-0.07968	0.704992
Streptococcus spp.	ABC	-0.34274	0.08652
Streptococcus spp.	ABC.S	-0.25223	0.21383
Streptococcus spp.	ABC.R	-0.14521	0.479096
Streptococcus spp.	ABC.B	-0.19715	0.334357
Streptococcus spp.	ABC.L	-0.10711	0.602518
Streptococcus spp.	ABC.S.1	-0.33208	0.097437
Streptococcus spp.	RBS-R	0.000342	0.998677
Streptococcus spp.	ADOS.LC	0.42499	0.01922
Streptococcus spp.	ADOS.RSI	0.168891	0.409499
Streptococcus spp.	ADOS.Play	0.016783	0.935144
Streptococcus spp.	ADOS.SBRI	0.334002	0.095394
Streptococcus spp.	ADOS	0.545302	0.003964
Streptococcal phages	GI	0.240544	0.236534
Streptococcal phages	CARS	-0.03772	0.857927
Streptococcal phages	ABC	-0.04755	0.817592
Streptococcal phages	ABC.S	0.088539	0.66712
Streptococcal phages	ABC.R	0.108562	0.59757
Streptococcal phages	ABC.B	-0.06515	0.751863
Streptococcal phages	ABC.L	-0.2585	0.202285
Streptococcal phages	ABC.S.1	0.13585	0.508156

Streptococcal phages	RBS-R	0.237427	0.242847
Streptococcal phages	ADOS.LC	0.611161	0.000315
Streptococcal phages	ADOS.RSI	0.045997	0.823439
Streptococcal phages	ADOS.Play	-0.0151	0.941619
Streptococcal phages	ADOS.SBRI	0.155914	0.446908
Streptococcal phages	ADOS	0.518192	0.006692
Streptococcal Vfs	GI	-0.21895	0.282541
Streptococcal Vfs	CARS	-0.25043	0.227272
Streptococcal Vfs	ABC	-0.36811	0.064271
Streptococcal Vfs	ABC.S	-0.07414	0.718895
Streptococcal Vfs	ABC.R	-0.2658	0.18938
Streptococcal Vfs	ABC.B	-0.38169	0.054344
Streptococcal Vfs	ABC.L	-0.23125	0.255693
Streptococcal Vfs	ABC.S.1	-0.158	0.440764
Streptococcal Vfs	RBS-R	-0.28486	0.158396
Streptococcal Vfs	ADOS.LC	0.448365	0.011364
Streptococcal Vfs	ADOS.RSI	-0.05075	0.805536
Streptococcal Vfs	ADOS.Play	-0.03945	0.848273
Streptococcal Vfs	ADOS.SBRI	0.045057	0.826993
Streptococcal Vfs	ADOS	0.376836	0.082582
